# Degree-of-polarization modulation for high-dimensional optical computing

**DOI:** 10.1038/s41586-026-10891-z

**Published:** 2026-08-12

**Authors:** Alessandro Petrini, Claudio Conti, Davide Pierangeli

**Affiliations:** 1 Department of Physics, Sapienza University of Rome, Rome, Italy; 2 Department of Basic and Applied Sciences for Engineering, Sapienza University of Rome, Rome, Italy; 3 Institute for Complex Systems, National Research Council, Rome, Italy

**Keywords:** Optical techniques, Applied physics, Applied optics

## Abstract

Spatial light modulation is a cornerstone of modern photonics. Crucial advances in photonic information processing^1–3^ rely on the spatial manipulation of the optical phase^4–12^ and state of polarization (SOP)^13–27^. The degree of polarization (DOP)^28–30^ is a fundamental property that can serve as an extra resource. However, no technology exists at present that can spatially modulate the DOP in a programmable manner^31–34^. Here we demonstrate spatial DOP modulation, thereby achieving control over a new degree of freedom of light. By engineering the polarization statistics at the micrometre scale with a phase-only spatial light modulator, we realize more than 1,024 spatial modes with fully programmable SOP and DOP. This structured light, sculpted in its polarization content to arbitrary shapes, enables direct encoding of information in a high-dimensional space. We encode colour images into a single-wavelength laser by using a one-to-one mapping between the red–green–blue space and the volume of the Poincaré sphere. Polarization colours expand the dimensionality of optical computing and encryption schemes, as demonstrated by (1) fully parallel photonic classification of colour images by a high-dimensional photonic neural network and (2) high-security multidimensional optical encryption. These approaches to optical processing of high-dimensional data highlight the opportunities enabled by DOP modulation in photonics, cryptography and computing.

## Abstract (web summary)

By engineering the statistics of light at the micrometre scale using phase-only spatial light modulators, both the state and degree of polarization become spatially programmable, enabling direct encoding of information in a high-dimensional space.

## Main

Spatial modulation of the phase, amplitude and polarization lies at the core of every modern optical system, driving advances in emergent fields such as neuromorphic photonics^1^, structured-light communication^2^ and optical cryptography^3^. Programming the phase profile enables large-scale photonic implementations of deep neural networks (DNNs)^4–7^, reservoir computing^8–10^, Ising machines^11^ and generative models^12^. Manipulation of the SOP enhances this potential by offering a powerful multiplexing strategy and input–output operations inaccessible by scalar fields, unlocking polarization-based computing^13–15^ and encryption^16–19^. New methods for shaping^20–24^ and measuring^25,26^ the polarization across space are pivotal towards photonic technologies that make use of the high-dimensional nature of light^27^.

The DOP is a statistical degree of freedom that expands the space available for optical information encoding. Inherent in any optical system, the DOP quantifies the variation of the SOP in the temporal, spectral or spatial domain. Rephrasing the Feynman Lectures on Physics^28^, an electromagnetic wave is always polarized in nature and the DOP emerges from the measurement of an incoherent ensemble of waves. Light is partially polarized or unpolarized when the detection time is larger than the polarization fluctuation time^29^ or the detection area exceeds the coherence length^30^. DOP tuning has been realized with designed metasurfaces^31^ and by engineering the temporal^32^ or spatial^33^ integration performed by the detector. Although these methods are effective for a homogeneous laser beam, DOP modulation within the wavefront remains challenging, as it requires a photonic unit able to control locally SOP fluctuations. Static metasurfaces lack pixel-by-pixel programmability^31^, whereas attempts using time-switching liquid crystals have been hindered by their low operating frequency^34^. So far, no technique exists for arbitrarily modulating both the DOP and the SOP across space.

We address this gap by introducing a statistical approach to control the DOP. Using a spatial light modulator (SLM), we impose stochastic SOP variations at the micrometre scale and tailor their probability distribution function (PDF). The resulting spatial DOP modulator enables information encoding by means of the volume of the Poincaré sphere. The term ‘high dimensional’ refers to access this space. We exploit this capability to encode colour images in a single-wavelength beam. By decoupling colour from optical frequency, we introduce a scheme to encode red–green–blue (RGB) information in optical neural networks by means of high-dimensional channels. In contrast to previously reported photonic processors, we process RGB images by exploiting several degrees of freedom of the optical field, integrating a statistical quantity—the DOP—alongside deterministic properties. Notably, we observe a performance increase by increasing the number of optical layers despite their linearity, suggesting that an effective nonlinear behaviour emerges from the vectorial nature of the processing. The platform is widely accessible and scalable, as it uses mature SLM technology that supports millions of active pixels in a broad spectral range.

## Spatial DOP modulation via polarization statistics

Defined in terms of the Stokes parameters, the DOP $\rho =\sqrt{{S}_{1}^{2}+{S}_{2}^{2}+{S}_{3}^{2}}/{S}_{0}$ maps the optical field to a point at radial coordinate *ρ* within the unit Poincaré sphere (0 ≤ *ρ* ≤ 1). We generate light with controlled DOP from the spatial superposition of a set of microscopic spatial modes with different SOPs. The key to tuning *ρ* lies in the statistical properties of the ensemble. This approach enables spatial DOP modulation, as the polarization statistics can be independently tailored across distinct wavefront regions using SLMs.

**Fig. 1:**
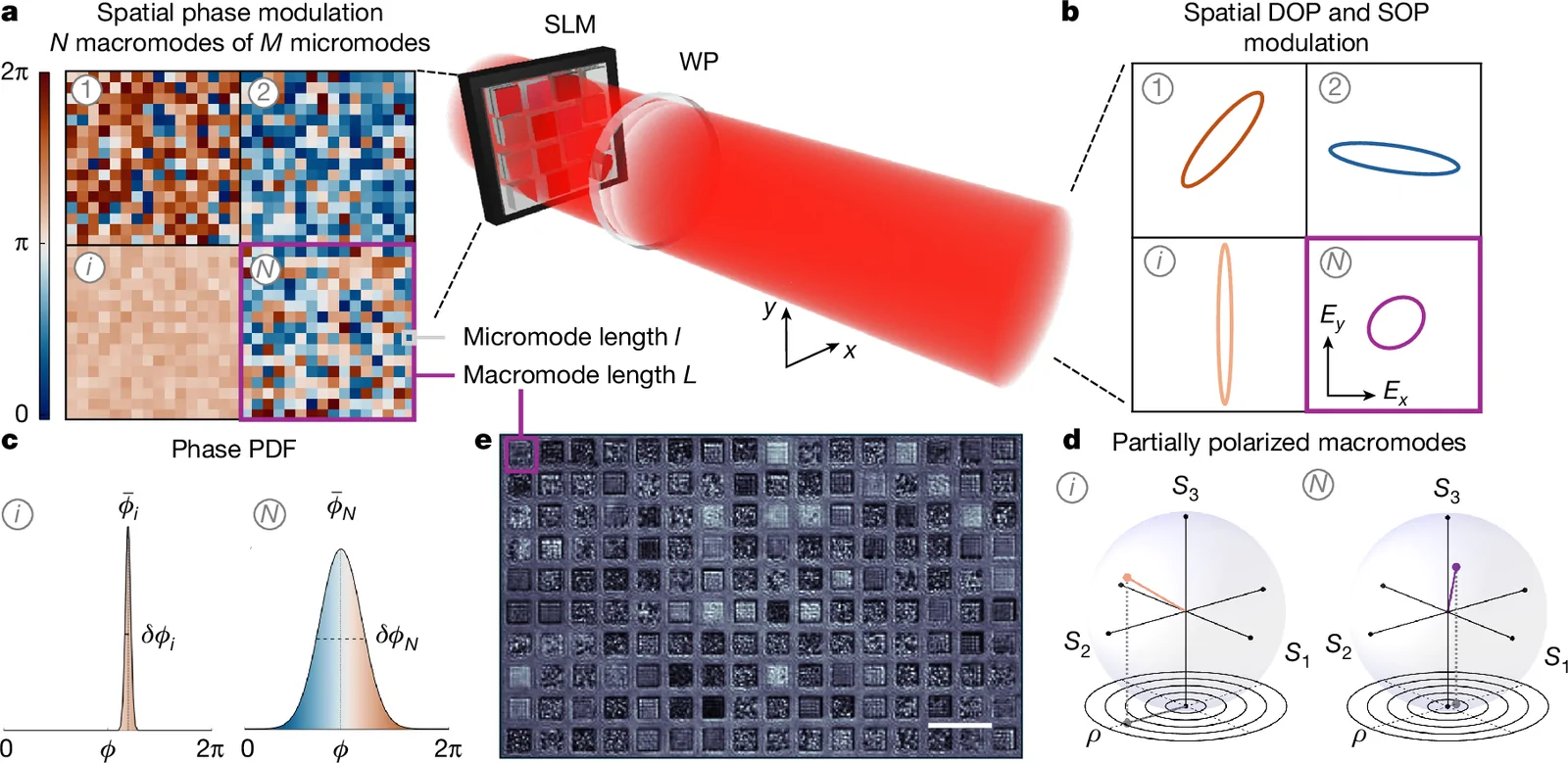
Spatial modulation of the DOP and the SOP. Light with spatially controlled DOP is synthesized by engineering the polarization statistics of an ensemble of spatially distributed waves. **a**, A phase-only SLM divides the wavefront of a coherent beam into macromodes (*i* = 1,…, *N*) composed of *M* micromodes with random phases. **b**, After a tunable WP, in the far field, the spatial phase modulation results in a beam structured in DOP and SOP, illustrated by different polarization ellipses. **c**, The phases in the *i*th macromode follow a Gaussian PDF with mean ${\bar{\phi }}_{i}$ and standard deviation *δϕ*_*i*_, which determine the *i*th point of the far-field beam inside the Poincaré sphere. **d**, The DOP is solely determined by *δϕ*_*i*_. **e**, Experimental image of part of the SLM plane viewed through a polarizer, showing hundreds of macromodes programmed through their microstructure. Scale bar, 0.5 mm.

Figure 1 illustrates the concept of the DOP modulator. The scheme makes use of phase modulation imparted by the SLM on a fully polarized laser. The beam is divided into *N* macromodes (Fig. 1a), with the *i*th macromode (*i* = 1,…, *N*) composed of *M* micromodes with random phases ${\phi }_{j}^{(i)}$ (*j* = 1,…, *M*). This phase mask generates a polarization-structured beam in the far-field *x*–*y* plane (Fig. 1b) by transmission through waveplates (WPs) (Methods and Supplementary Note 1). The set-up makes every SLM pixel act as a polarization modulation unit. Micromodes correspond to different SOPs on a micrometric scale. The SOPs of a macromode mix in the far field, yielding a single effective state within the Poincaré sphere determined by the micromode PDF. We develop two different approaches to engineer the position of the target beam, while avoiding crosstalk between macromodes. The first relies on free-space propagation over a distance larger than the diffraction length of the micromode but smaller than the diffraction length of the macromode. The second implements the modulation in the Fourier plane. We focus here on the first case and the Fourier-plane implementation is demonstrated using a microlens array in Supplementary Note 6. The DOP and SOP at the *i*th far-field position are controlled by the PDF *P*^(*i*)^(*ϕ*) that governs the phases of the constituent micromodes. We consider a Gaussian PDF ${\mathcal{N}}(\bar{\phi },\delta \phi )$ with mean phase $\bar{\phi }$ and standard deviation *δϕ*. The two programmable parameters ${\bar{\phi }}_{i}$ and *δϕ*_*i*_ control the state of the *i*th point, as illustrated in Fig. 1c,d. In both experiments and theory, we find that *ρ* is solely determined by *δϕ*. This establishes a general strategy for controlling the DOP using a phase-only modulation device. To achieve complete programmability of the SOP and the DOP in every macromode, we also implement the concept by replacing the WPs with an extra SLM that operates as a spatially tunable retarder. In this fully programmable configuration (Supplementary Note 4), all possible states in the volume of the Poincaré sphere can be generated within a single beam. Figure 1e shows part of the SLM plane, imaged through a polarizer, for a beam containing 1,024 macromodes of arbitrary DOP and SOP.

## On-demand states inside the Poincaré sphere

**Fig. 2:**
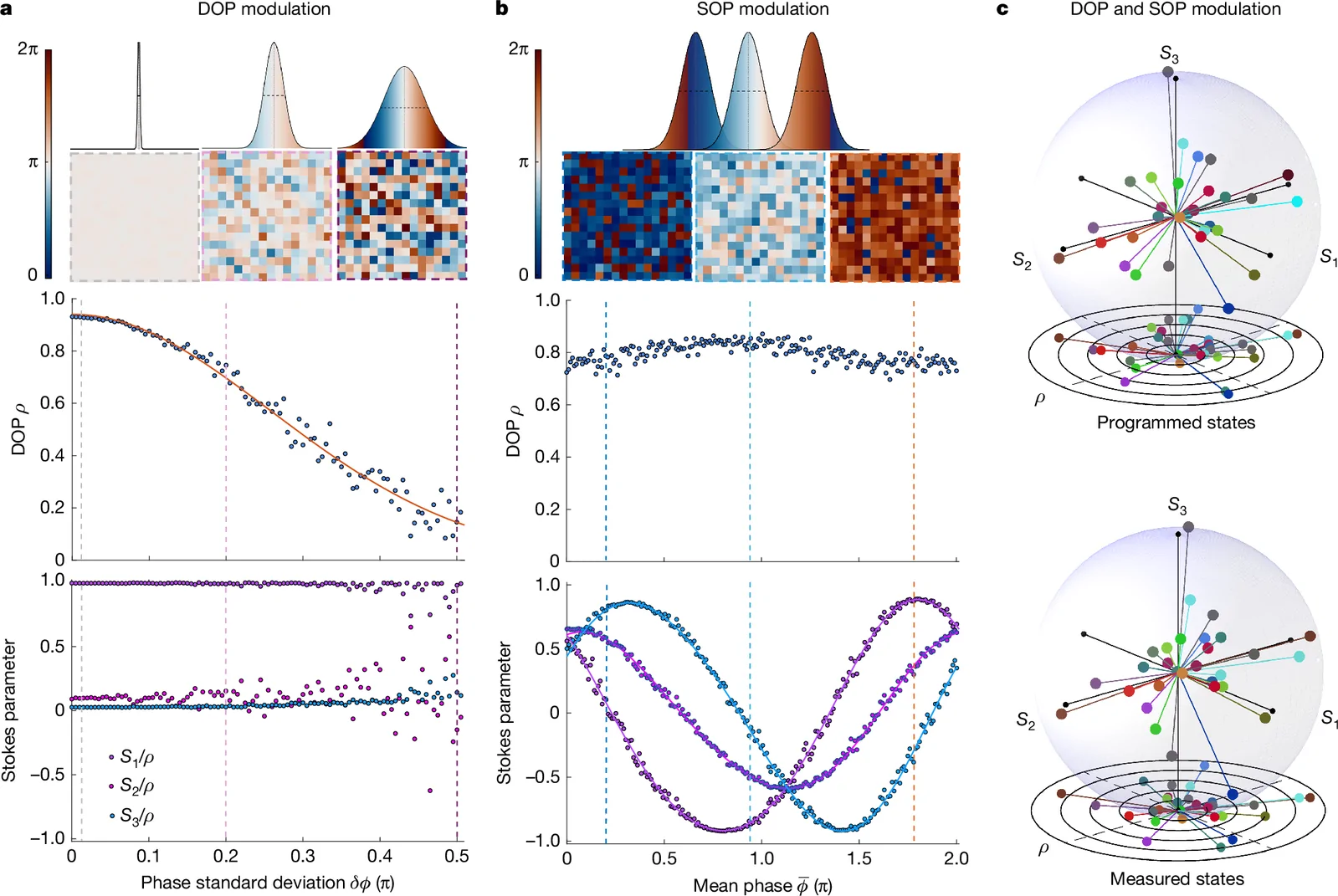
Control of DOP and SOP through the phase probability distribution. **a**, DOP-only modulation. Measured *ρ* (dots) with exponential fit (line) based on coherence theory, and Stokes parameters *S*_1_, *S*_2_ and *S*_3_ as a function of *δϕ* for $\bar{\phi }={\rm{\pi }}$. **b**, SOP-only modulation. Polarimetric measurements while scanning $\bar{\phi }$ at constant *δϕ* = 0.15π. Insets in **a** and **b** show phase masks (*M* = 256) for representative values of *δϕ* and $\bar{\phi }$ (dashed lines), together with the corresponding PDF. **c**, Controlled sampling of the volume of the Poincaré sphere. Generated states compared with programmed targets (same colours).

We first consider a single macromode and demonstrate control of both DOP and SOP by tuning the phase PDF. The experimental set-up (Methods and Supplementary Fig. 1) uses a continuous-wave laser at *λ* = 532 nm. The generated beams are analysed with a rotating-WP polarimeter that measures *S*_1_, *S*_2_, *S*_3_ and *ρ*. Results obtained from random masks sampled from ${\mathcal{N}}(\bar{\phi },\delta \phi )$ at a fixed $\bar{\phi }$ and varying *δϕ* (Fig. 2a) show a continuous and wide modulation of *ρ*, demonstrating the effectiveness of the statistical method. The DOP spans the entire interval [0, 1], whereas the SOP remains constant. Nearly depolarized light (*ρ* = 0.08) is achieved for a broad PDF (*δϕ* = 0.5). The experimental data are well described by the relation *ρ* = exp(−*δϕ*^2^/2), as predicted by our polarization matrix model (Supplementary Note 2) based on coherence theory^30^. Different mask instances drawn from the same PDF give the same measured *ρ*. SOP control at constant *ρ* is shown in Fig. 2b. The phase masks now correspond to PDFs with different $\bar{\phi }$ and constant *δϕ*. As indicated by the oscillations in *S*_1_, *S*_2_ and *S*_3_, the SOP traces an ellipse on a sphere of fixed radius (*ρ* = 0.8) as $\bar{\phi }$ is varied.

Figure 2a,b demonstrates independent tunability of the DOP and SOP. Combining both operations, we achieve on-demand generation of arbitrary states within the Poincaré sphere. We calibrate the modulator so that a target point [*S*_1_, *S*_2_, *S*_3_] directly maps to *δϕ* and $\bar{\phi }$. Figure 2c shows measured states compared with the corresponding targets. Inaccuracies mainly arise from WP rotations. This limitation is removed in the two-SLM set-up (Supplementary Note 4). We achieve errors of 4 ± 1%, 2 ± 1° and 2 ± 1° for *ρ*, azimuthal angle *θ* = arctan(*S*_2_/*S*_1_)/2 and ellipticity angle *χ* = arcsin(*S*_3_/*S*_0_)/2, respectively, demonstrating high modulation accuracy.

## Light structured in DOP and SOP

**Fig. 3:**
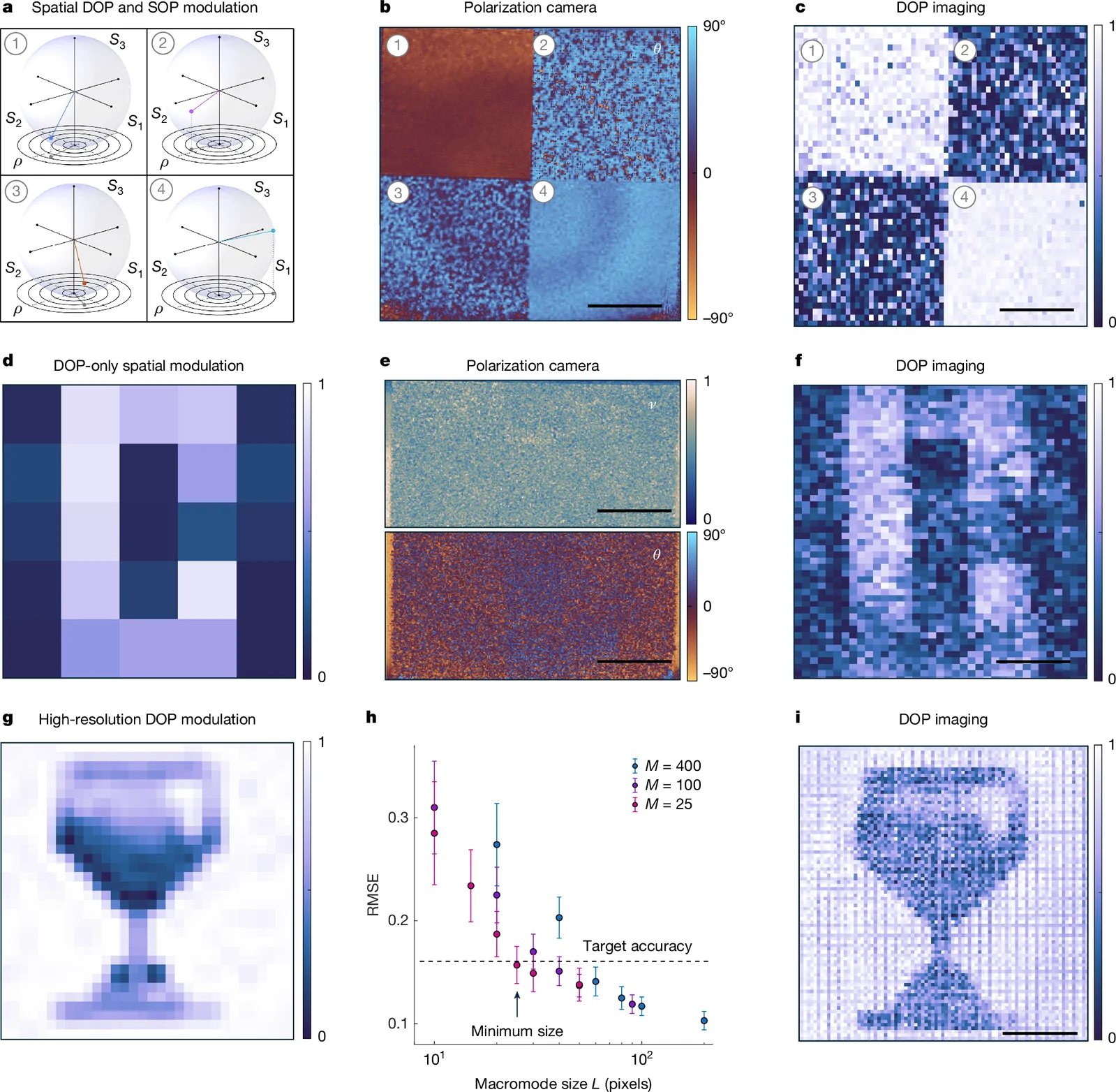
DOP-structured light. **a**, Programmed DOP and SOP for *N* = 4 macromodes. **b**, Azimuth image of the beam measured by the polarization camera (method 1). **c**, DOP image measured by full-Stokes imaging (method 2). **d**, DOP-only spatial modulation. The letter ‘C’ is written exclusively in the DOP profile. **e**, The pattern is invisible in both the linear polarization degree *ν* and azimuth *θ*. **f**, Measured DOP revealing the encoded letter. **g**, DOP encoding of a high-resolution greyscale image. **h**, Scaling analysis of the modulation accuracy as the macromode size *L* is reduced for different *M*. The modulation remains accurate down to a minimum size of *L* = 25 pixels. **i**, DOP image of the encoded glass picture (*N* = 1,024). Scale bars, 3 mm (**b**,**c**,**e**,**f**,**i**). Panel **d** is adapted from the MNIST handwritten digit database^51^. Panel **g** is adapted from the Pixel Art dataset^52^.

Spatial DOP modulation is demonstrated in Fig. 3. We generate a target beam with *N* = 4 (Fig. 3a) by setting the parameters (*δϕ*_*i*_, ${\bar{\phi }}_{i}$). The beam has a uniform intensity, being all information encoded in Stokes space. We first analyse the polarization profile using a non-full-Stokes polarization camera (method 1), which measures the azimuthal angle *θ* and linear polarization degree *ν*. The presence of a polarization pattern is evident in the azimuth image (Fig. 3b). However, the polarization camera is unable to measure *ρ*. We thus perform full-Stokes measurements with an intensity camera (method 2). The Stokes maps are reported in Supplementary Fig. 5. The measured DOP image (Fig. 3c) clearly resolves the four square macromodes with the programmed *ρ*. Figure 3d reports the handwritten letter ‘C’ that we sculpt in the sole DOP profile with a spatial resolution of *N* = 25. The presence of the pattern is not detectable even with the polarization camera. Maps of *θ* and *ν* (Fig. 3e) show no visual trace of the letter. The encoded greyscale image is successfully visualized (Fig. 3f) by DOP imaging (method 2).

To demonstrate the modulator scalability and the encoding of patterns at higher resolution, we analyse the modulation accuracy by full-Stokes imaging as the macromode length *L* is reduced. The root mean square error (RMSE) as a function of *L* and *M* (Fig. 3h) indicates accurate modulation even for small macromodes. Relevant deviations from the target states occur when the micromode size *l* becomes comparable with *L*, which invalidates the statistical approach. We define the minimum macromode size supported by the set-up by choosing a target accuracy of RMSE = 0.15, which yields *L* = 25 pixels at *l* = 5 pixels. This allows us to arbitrarily program up to *N* = 1,024 macromodes. To avoid macromode crosstalk, we introduce blank pixels between macromodes on the SLM. Figure 3i reports the DOP image of a glass picture written at this resolution (*N* = 32 × 32). The encoded image (Fig. 3g) is faithfully reproduced, with the buffer area visible as a polarized background grid.

## Polarization colours

**Fig. 4:**
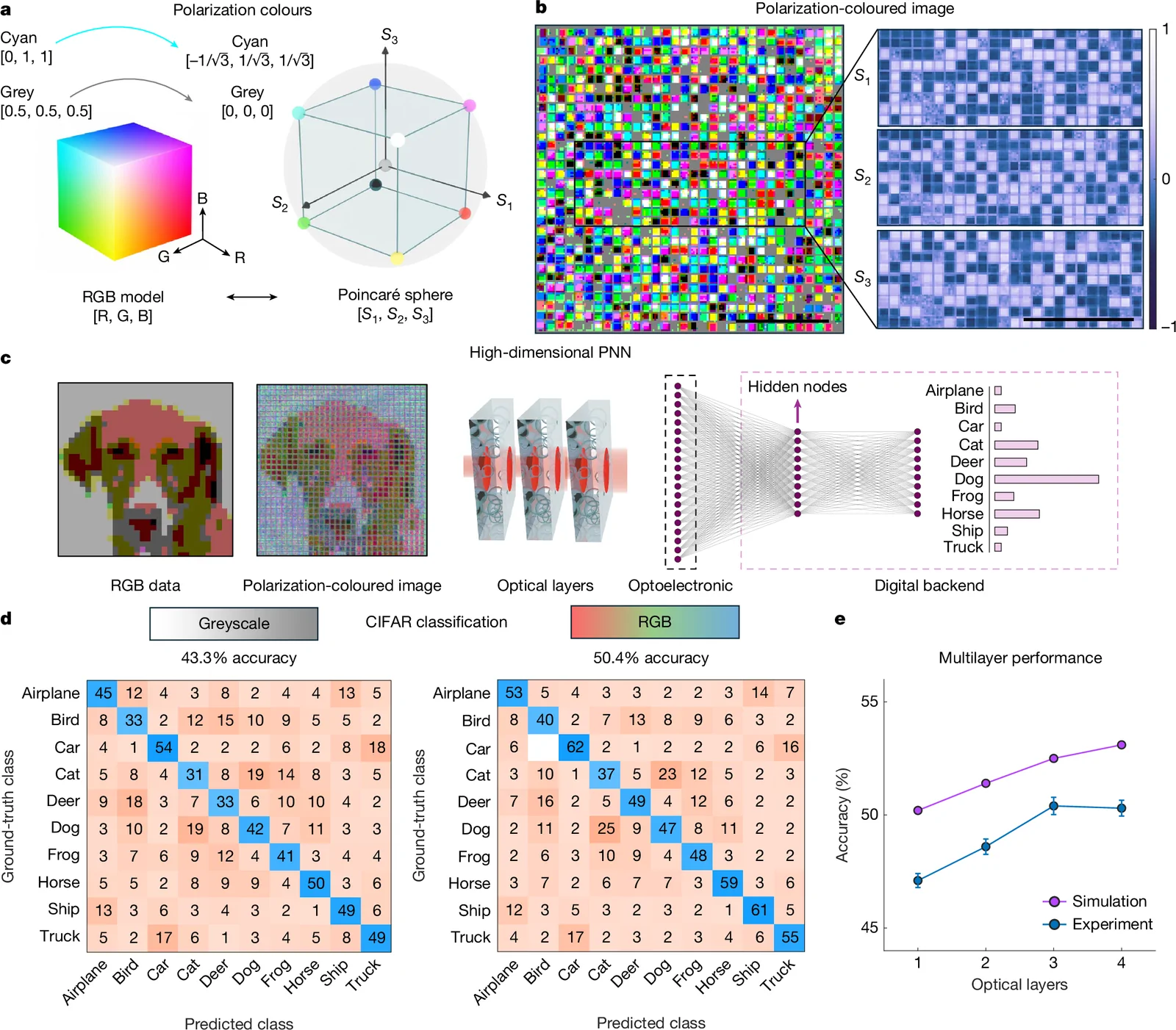
Polarization encoding of colour images and high-dimensional PNN. **a**, Polarization colours defined by mapping the RGB colour cube into the volume of the Poincaré sphere using a one-to-one correspondence with Stokes coordinates. **b**, Experimental demonstration of polarization-coloured images. A colour mosaic is generated by means of DOP and SOP modulation, with each of the 32 × 32 RGB pixels mapping to a macromode. Measured colours are reconstructed from the Stokes images (insets) using inverse mapping. **c**–**e**, PNN architecture and performance. **c**, Scheme of the free-space PNN for colour image classification. The RGB sample is polarization-encoded, as illustrated by the experimental image of a dog, and processed fully in parallel by a series of random optical layers made by ground-glass diffusers. A few-node digital backend is trained by means of supervised learning. **d**, Advantage of the high-dimensional optical computation. Confusion matrices (%) on the CIFAR-10 test set for *n* = 3 optical layers. RGB encoding yields higher accuracy than greyscale (phase-only) encoding (50.4% versus 43.3%). **e**, Classification accuracy versus the number of optical layers. Scale bars, 3 mm (**b**). Panel **c** is adapted from the CIFAR-10 dataset^53^.

The modulator enables optical encoding of three-dimensional information at every point in space. As a key example, we encode colour images in a single-wavelength beam profile. We establish a one-to-one mapping between the RGB colour space and the volume of the Poincaré sphere. The three colour channels [R, G, B] map onto the Stokes coordinates [*S*_1_, *S*_2_, *S*_3_], forming what we term polarization colours. This transformation (Methods) inscribes the RGB colour cube, which defines all possible colours in the RGB model, inside the Poincaré sphere (Fig. 4a). Genuine polarization colours cannot be achieved using the SOP alone. In the RGB model, any colour is expressed as a linear combination of the three primaries, which requires three independent quantities. The SOP alone cannot provide this property, as the corresponding Stokes parameters are not independent but constrained on the sphere surface. The DOP therefore provides the missing quantity required for colour-polarization encoding.

Through polarization colours, an RGB image is directly mapped onto a beam structured in DOP and SOP, in which each original RGB pixel corresponds to a macromode. We define a polarization-coloured image as an optical wavefront composed of spatially distributed polarization colours. Figure 4b shows an experimental realization of a polarization-coloured image. We encode a 32 × 32-pixel colour mosaic, which is reconstructed from the measured Stokes images (Fig. 4b, inset) by means of the inverse mapping. The modulator can encode any RGB image with 8-bit precision per colour channel.

## High-dimensional optical computing

Polarization encoding of colour images is particularly useful for optical neural networks, in which RGB data processing is typically avoided owing to the complex multiplexing required. Using our spatial DOP modulator, we realize a photonic neural network (PNN) that encodes RGB images into high-dimensional channels and processes them in a single-shot operation. Our architecture (Fig. 4c) belongs to the broader class of PNNs based on SLMs and complex media^35–37^, which feature large-scale operation and fast training, but generalizes these free-space PNNs by increasing their dimensionality. In our implementation, the field encodes a three-dimensional vector at each spatial mode and optical processing is partially coherent. This concept differs fundamentally from PNNs operating on individual colour channels.

The high-dimensional PNN consists of cascaded random optical layers implemented with ground-glass diffusers, an intensity camera that detects the transmitted speckle pattern and forms an optoelectronic nonlinear layer, and a trainable few-node digital backend (Methods). The RGB image is polarization encoded, optically processed and digitally classified, all within a single pass (Fig. 4c). Data encoding is inherently nonlinear, as the Stokes parameters are quadratic functions of the field. Figure 4d reports performance on the CIFAR-10 classification task. We achieve 50.4% accuracy on the 10,000-image test set across ten categories, a result that matches the state-of-the-art performance of multichannel PNNs^37^. The encoding of RGB images in polarization substantially improves the classification accuracy with respect to a phase-only encoding of greyscale images (43.3%). The performance enhancement arises from two mechanisms. The first is general: colours provide further information about the scene, helping to identify the class by their correlation with the object (for example, the sky is often blue). The second mechanism arises from the vector nature of the field through the operation performed by the optical layers. Specifically, the polarization-encoded input is processed by a complex matrix that has a larger rank than its scalar counterpart, as it accounts for the polarization response of the medium. The finding is confirmed by simulations of the PNN (Supplementary Fig. 13), in which the optical layers are modelled in the framework of the vector transmission matrix (VTM)^38^, as detailed in Supplementary Note 7. The enhanced accuracy persists even for a PNN made of ten digital hidden nodes (Supplementary Fig. 14), indicating that polarization encoding also facilitates computing with minimal digital resources. Notably, we observe an accuracy increase when more diffusers are cascaded (Fig. 4e), despite these being linear layers. This peculiar effect is a distinctive feature of the information processing based on polarization, as it originates from the properties of the VTMs that characterize the diffusers (Supplementary Note 7).

The DOP modulator can be exploited as a high-dimensional encoder in many other types of PNN. We realize a high-dimensional photonic extreme learning machine (PELM), a framework in reservoir computing. PELMs require only a linear digital layer with a simplified training by ridge regression^10^. Our implementation uses the cascaded diffusers as an optical reservoir. Results on the CIFAR-10 dataset (Supplementary Fig. 15) show an accuracy improvement of 6% over the same PELM based on phase encoding, which further validates the computing advantage enabled by the polarization.

## Secure encryption of multidimensional data

**Fig. 5:**
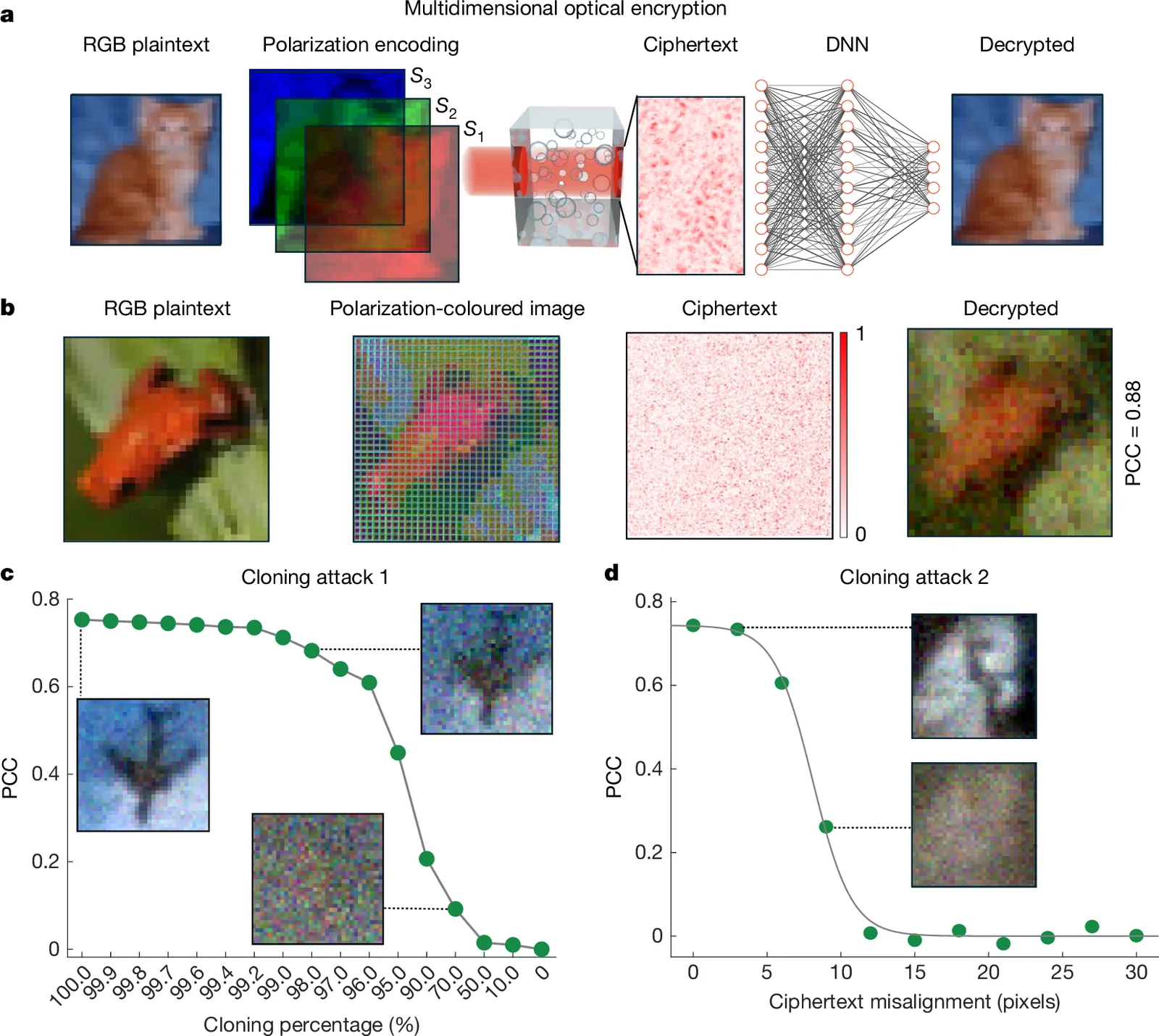
DOP-enabled multidimensional optical encryption. **a**, Speckle-based encryption and decryption system. The RGB image (plaintext) is polarization-encoded and encrypted into a speckle pattern (ciphertext) by a scattering medium. A DNN performs the decryption. **b**, Encryption and decryption of a 32 × 32 RGB image. Decryption fidelity is quantified by the PCC. **c**,**d**, Security against cloning attacks. **c**, PCC versus the fraction of cloned output weights. **d**, PCC versus ciphertext misalignment. Insets in **a** and **b** are adapted from the CIFAR-10 dataset^53^.

Encoding colour images within the beam wavefront is also highly relevant for optical encryption^39^. Our DOP modulator enables this functionality, extending the dimensionality of optical encryption schemes. We consider speckle-based optical encryption^40,41^, which has recently attracted attention for its high security^42^, but it is still limited to a single channel. This scheme encrypts a plaintext, such as a greyscale image, into an intensity speckle by using optical scattering as a physical key and a DNN for fast decryption. We encrypt RGB images as plaintexts encoded through SOP and DOP, thereby extending the scheme to multidimensional data (Fig. 5a). The encryption process exploits the VTM of the scattering medium as an unclonable key of huge size, which is learned and encoded in the network weights. Once trained, the network deciphers previously unseen ciphertexts by recovering the full polarization content of the input beam from a single-shot intensity measurement. Figure 5b shows the encryption and decryption of a 32 × 32 RGB plaintext. Both spatial and colour features are recovered with high fidelity, as quantified by the Pearson correlation coefficient (PCC) between the original and decrypted images. The robustness of the ciphertext against detection noise is verified in Supplementary Fig. 16. Compared with coherent speckle encryption^40–42^, the use of partially polarized light results in a speckle pattern with reduced contrast, smoothing the differences between diverse ciphertexts. The effect reinforces security by increasing the complexity of the ciphertext-to-plaintext mapping.

Strong security is guaranteed by the 12.2-Gbit decryption key embedded in the network (Methods). We analyse the protection against two high-level cloning attacks. In the first, the attacker achieves a perfect clone of the ciphertext but lacks a small fraction of the DNN parameters. Figure 5c shows the decryption accuracy as a function of the cloning percentage of the last fully connected layer. Missing only 1% of the output weights causes a poor decryption. In the second attack, the DNN is cloned perfectly but the ciphertext is intercepted with a minor systematic error. A misalignment of a few camera pixels is sufficient to prevent unauthorized decryption (Fig. 5d). Moreover, polarization encoding inherently provides an extra security key. Even if an attacker perfectly clones both the network and the ciphertext, the original image cannot be obtained without knowing the exact mapping [R, G, B] → [*S*_1_, *S*_2_, *S*_3_] (security key) that is chosen by the sender and shared securely with the receiver (Supplementary Fig. 17). These features establish a polarization-protected cryptosystem for multidimensional data.

## Discussion

Limited control of the DOP has so far hindered its use in photonic applications. We have demonstrated spatial DOP modulation as a new resource for optical computing and encryption. The method relies on tailoring the statistics of an ensemble of SOPs, a concept that can also be implemented in integrated systems such as active metasurfaces or in the time domain. Our scheme based on assembling SLM pixels provides an effective strategy to encode high-dimensional information while maintaining the massive parallelism of spatial optics. In SLM-based optical processors, pixels are always grouped into uniform spatial modes, as single-pixel addressing leads to poor signal-to-noise ratios and reduced accuracy. Our approach turns this constraint into an advantage: unavoidable pixel grouping becomes an extra degree of freedom, effectively turning unused pixels into a high-dimensional information channel. The number of macromodes scales linearly with the number of available pixels (Methods). The increasing resolution of liquid-crystal-on-silicon displays makes the approach highly scalable. Notably, the spatial DOP modulator can operate on top of any phase-modulation technology, including micro-electromechanical systems SLMs^43^ and emerging electro-optic SLMs^44–46^ with GHz modulation rates. This versatility enables integration into high-throughput PNNs for low-latency classification, regression and generative tasks, as well as into neuromorphic devices for imaging and sensing^26^. Encoding in the volume of the Poincaré sphere can extend the functionality of various PNNs, including diffractive^47^, on-chip^48^ and nonlinear-wave^49^ processors, representing a key tool for photonic computing, from generative artificial intelligence^12^ to probabilistic bits^50^.

In conclusion, by demonstrating spatial DOP modulation and its integration into optical neuromorphic systems to augment their dimensionality, our work establishes DOP as a powerful degree of freedom that can play a central role in emerging photonic technologies.

## Methods

### Experimental set-up

The set-up consists of the spatial DOP modulator interfaced separately with the high-dimensional PNN and the encryption–decryption system. These two blocks make a different use of scattering media and digital neural networks and are described hereafter in dedicated subsections.

The DOP modulator is composed of a phase-only liquid-crystal-on-silicon SLM (Hamamatsu X13138, 1,280 × 1,024 pixels, 12.5 μm pixel pitch, 60 Hz frame rate) sandwiched between an input half-waveplate (HWP) and an output pair composed of a quarter-waveplate (QWP) and a HWP. An expanded beam from a continuous-wave laser (*λ* = 532 nm, 250 mW), with diagonal (D) polarization set by the input HWP, illuminates the SLM. The output QWP and HWP are mounted on high-speed motorized rotation stages and oriented at angles *α* and *β*, which are programmed together with the SLM. The WPs convert the phase delay *ϕ* imparted by a SLM pixel into a SOP set by (*ϕ*, *α*, *β*), as detailed in Supplementary Note 1. The DOP and SOP of a macromode are controlled through the four parameters (*δϕ*, $\bar{\phi }$, *α*, *β*). In the two-SLM implementation (Supplementary Fig. 4), two identical SLMs (Hamamatsu X15213-16L, 1,280 × 1,024 pixels, 12.5 μm pixel pitch, 60 Hz frame rate) are cascaded pixel-to-pixel by means of a 4*f* lens system with an inserted HWP at a fixed angle *γ* = 22.5°. The modulator is calibrated using polarimetry measurements performed by a rotating-WP polarimeter (Thorlabs PAX1000VIS, 0.25° accuracy) that measures *S*_1_, *S*_2_, *S*_3_ and *ρ*.

Spatial modulation of the DOP and SOP is realized in two different configurations. In the first, the modulated beam is observed in a far-field plane located at a distance *z* from the SLM, whereas in the second, it is observed in the Fourier plane. We detail here the first configuration, as the experimental set-up is more versatile and does not require further optical components, and the Fourier implementation by means of a microlens array is detailed in Supplementary Note 6. The working distance *z* is set according to the micromode size *l*, which determines the diffraction length after which micromodes mix by propagation. At full resolution (*N* = 32 × 32), *z* is set to approximately 5 cm. For this *z*, the size of the macromode formed in the far field is comparable with its size *L* on the SLM.

The modulator is validated by a non-full-Stokes polarization camera (method 1) and a full-Stokes imaging system (method 2). The polarization camera (Thorlabs Kiralux, 2,448 × 2,048 pixels) acquires images (Fig. 3) of the linear polarization degree $\nu =\sqrt{{S}_{1}^{2}+{S}_{2}^{2}}/{S}_{0}$, azimuth *θ* = arctan(*S*_2_/*S*_1_)/2 and intensity *S*_0_(*x*, *y*). Full-Stokes and DOP imaging is performed by carrying out Stokes measurements with the camera in intensity mode, that is, sequentially acquiring intensity projections of the beam profile through a QWP and a polarizer at different orientations^54^. The accuracy of the spatial DOP and SOP modulation is evaluated by the ${\rm{RMSE}}=\frac{1}{N}{\sum }_{i}^{N}\sqrt{{\sum }_{k}|{S}_{k}^{{\rm{m}}}-{S}_{k}^{{\rm{p}}}{|}^{2}/3}$, in which superscripts ‘m’ and ‘p’ denote measured and programmed values, respectively.

### Programming the spatial DOP modulator

The SLM active area is divided into *N* square macromodes (blocks of pixels). A macromode is further divided into *M* square micromodes, each consisting of *l* × *l* pixels, with *l* properly set to fill the SLM active area for a target resolution *N*. For instance, we use *l* = 12 pixels for *M* = 256 and *N* = 25 (Fig. 3d), that is, the micromode size is 150 μm in this case. The minimum macromode size required for accurate spatial modulation of the DOP and SOP is *L* = 25 pixels, achieved by using *M* = 25 micromodes of length *l* = 5 pixels (Fig. 3h). For high-resolution modulation (Fig. 3i), a few blank pixels of constant polarization are used to separate the macromodes and avoid their overlap owing to diffraction. The phase mask is constructed by assigning to all the pixels of the *j*th micromode a constant phase *ϕ*_*j*_ in the interval [0, 2π]. The value *ϕ*_*j*_ is randomly extracted from a Gaussian PDF that characterizes the *i*th macromode, ${{\mathcal{N}}}^{(i)}(\phi )=(1/\sqrt{2{\rm{\pi }}\delta {\phi }_{i}^{2}})\exp [-{(\phi -{\bar{\phi }}_{i})}^{2}/2\delta {\phi }_{i}^{2}],$ with standard deviation *δϕ*_*i*_ in [0, π/2] and mean ${\bar{\phi }}_{i}$ in [0, 2π]. By varying $\bar{\phi }$, the SOP spans a trajectory on the Poincaré sphere that is tunable by the WP angles.

We calibrate the modulator by performing the analysis in Fig. 2 at different values of (*δϕ*, $\bar{\phi }$, *α*, *β*). In Fig. 2, each data point corresponds to a single-mask experiment. Note that, as *ρ* tends to zero, the polarized component becomes less definite and, consistently, the measurement error on the Stokes parameters is larger. Averaging over several statistically equivalent masks allows us to reduce the noise observed in single-mask experiments (Supplementary Fig. 2). The DOP is calibrated using the average modulation and the fitting function *ρ* = *a*exp(−*bδϕ*^2^) + *c*. The measured SOP (Fig. 2b) is in close agreement with the polarization matrix model (Supplementary Note 2). We then construct a mapping between (*S*_1_, *S*_2_, *S*_3_, *ρ*) and the four parameters (*δϕ*, $\bar{\phi }$, *α*, *β*) = *X*. A target beam, spatially modulated in DOP and SOP, is generated by setting the vectors *X*^(*i*)^ accordingly. We study the dependence on the number of micromodes *M* in Supplementary Fig. 3. In the two-SLM implementation, the WP angles *α* and *β* are replaced by a second tunable phase ${\phi }_{2}^{(i)}$, which is set independently for each macromode and remains constant within it. In this case, the modulator is programmed by the vectors ${X}^{(i)}=(\delta \phi ,\bar{\phi },{\phi }_{2})$. The calibration of the two-SLM modulator is reported in Supplementary Fig. 5. The modulator is programmed using custom MATLAB codes.

### Avoiding macromode crosstalk

To control the spatial modulation of the DOP and SOP, it is crucial that macromodes do not interact with each other. Any macromode crosstalk would degrade the modulation accuracy, as the state programmed on a macromode would affect its neighbours. To avoid macromode crosstalk, the far-field distance *z* must be chosen appropriately. As the interaction between two close micromodes and two close macromodes occurs at a distance on the order of their diffraction lengths *z*_*l*_ = π*l*^2^/*λ* and *z*_*L*_ = π*L*^2^/*λ*, respectively, the working distance must satisfy *z*_*l*_ ≪ *z* ≪ *z*_*L*_. This condition is achieved easily for large macromodes (*l* ≪ *L*). For instance, *L* = 2.4 mm and *l* = 150 μm, as in Fig. 3d–f, yield approximately 0.1 m < *z* < 10 m. In this case, macromode crosstalk has a negligible effect. It becomes relevant when *L* and *l* are closer in value, as occurs when reducing *M* to maximize the number of addressable macromodes. In this case, crosstalk is avoided by using a few blank pixels that spatially separate adjacent macromodes. The length of this buffer area is chosen so that micromodes at the edge of two adjacent macromodes have no spatial overlap on propagation. The residual crosstalk is experimentally quantified in Supplementary Note 9. In Fig. 3i, in which *l* = 60 μm and *z* = 5 cm, we use *d* = 6 blank pixels. In the Fourier-plane implementation, macromode crosstalk is avoided by design because each microlens operates on a single macromode. This configuration is preferable for applications that require focused DOP-modulated light.

### Encoding colours in polarization

To encode genuine RGB colours in polarization, SOP modulation alone is not sufficient. In fact, although we could associate some colours to different SOPs, such a mapping to the sphere surface does not preserve the essential property that gives any colour as a linear combination of the primaries. To overcome this limitation, DOP modulation is necessary. We use the map illustrated in Fig. 4a, given by ${S}_{1}=(2{\rm{R}}-1)/\sqrt{3}$, ${S}_{2}=(2{\rm{G}}-1)/\sqrt{3}$ and ${S}_{3}=(2{\rm{B}}-1)/\sqrt{3}$, with R, G, B ∈ [0, 1]. Note that many other maps are possible, including transformations that use a nonlinear relation or the spherical coordinates [*θ*, *χ*, *ρ*] on the Poincaré sphere. We can encode RGB colours with a precision of up to 8 bits per channel, determined by the SLM bit depth.

### High-dimensional PNN

The optical part of the PNN is composed of *n* optical random layers formed by a stack of *n* diffusers (Thorlabs N-BK7 Ground Glass Diffusers with 120-, 200-, 600- or 1,500-grit polishes) and an optoelectronic layer implemented by a complementary metal–oxide–semiconductor (CMOS) camera (Basler a2A1920-160umPRO, 1,920 × 1,200 pixels, 12-bit pixel depth) positioned 10 cm away from the stack of diffusers. A 4 × 4-pixel binning is performed directly on the CMOS sensor, which implements an average pooling layer directly in hardware. The 300 × 300 acquired intensity values (12-bit precision) form the input to a digital backend. The digital network is a few-node network made of two fully connected layers with 40 hidden nodes and ten output nodes (output classes). The class is assigned by the softmax operation on the output vector and training is performed by using the Adam optimizer.

We classify colour images from the CIFAR-10 dataset^53^, which consists of 60,000 32 × 32 RGB images of ten object classes with 6,000 images per class. These are divided into 50,000 training samples and 10,000 test samples. For comparison, we also classify the corresponding greyscale images obtained by converting the original RGB dataset. Images are polarization-encoded into *N* = 32 × 32 macromodes of size *L* = 25 pixels (Fig. 4c). The RGB to Stokes mapping in Fig. 4a is used. This performs a nonlinear operation on the input data. Note that phase encoding is also nonlinear^55^. Therefore, the input nonlinearity has a minor role in the observed performance enhancement. Classification accuracy is averaged over repeated training and testing runs.

We model the high-dimensional PNN in terms of cascaded VTMs and partially coherent propagation^56^, as detailed in Supplementary Note 7.

### Scalability

As a high-dimensional encoder, the spatial DOP modulator supports a resolution that scales linearly with the number of SLM pixels, *N* = *ξ*^−1^*n*_px_, with *ξ* = *L*^2^ = *M* × *l*^2^ a set-up-dependent constant factor (Supplementary Table 1 reports a comparison of spatial optical encoders in PNNs). In our implementation with 32 × 32 macromodes, *ξ* ≈ 6 × 10^2^ (Fig. 3h). According to this value, more than 10,000 macromodes can be generated with ultrahigh-definition SLMs (4K, *n*_px_ = 4,160 × 2,464). Therefore, a large-scale implementation is readily achievable with off-the-shelf components.

### Multidimensional optical encryption system

Speckle-based encryption of polarization-encoded RGB images is performed using a 120-grit ground-glass diffuser as a scattering medium positioned in the focal plane of a lens (250 mm focal length), with the transmitted speckle pattern (ciphertext) collected by the CMOS camera. The speckle intensity is directly related to the input SOPs through the transmission tensor^57^ of the diffuser. The acquired intensity images (1,200 × 1,200 pixels, 4,096 intensity levels) are downsampled to form a vector of size 1 × 90,000. The DNN consists of two fully connected layers, with *w* × *N* hidden nodes and 3 × *N* output nodes, connected by means of batch normalization and ReLU activation. The hyperparameter *w* sets the hidden-layer size and is tuned to optimize the decryption accuracy (*w* = 4 for the results in Fig. 5). The 3 × *N* output vector contains the values *S*_1_, *S*_2_ and *S*_3_ of the *N* macromodes. The decrypted image is obtained by inverse mapping to the RGB values with the chosen relation [R, G, B] ↔ [*S*_1_, *S*_2_, *S*_3_]. The error owing to an incorrect map (security key) is shown in Supplementary Fig. 17.

The decryption DNN used in Fig. 5 has nearly 3.8 × 10^8^ learnable parameters (12.2 Gbit at 32-bit precision). It is trained on a dataset of 20,000 plaintext–ciphertext pairs. The fidelity of the decrypted image is quantified by the PCC, an easily interpretable metric. We can encrypt any RGB image up to 32 × 32 pixels. In Fig. 5, we encrypt CIFAR-10 images to demonstrate operation at the maximum supported resolution.

## Online content

Any methods, additional references, Nature Portfolio reporting summaries, source data, extended data, supplementary information, acknowledgements, peer review information; details of author contributions and competing interests; and statements of data and code availability are available at https://doi.org/10.1038/s41586-026-10891-z.

## Supplementary information

Supplementary Information Supplementary Notes 1–10, including Supplementary Figs. 1–18, Supplementary Table 1 and Supplementary References.

Peer Review File

## Data Availability

The data that support the findings of this study are publicly available on Zenodo at https://doi.org/10.5281/zenodo.20775840 (ref. ^59^).

## Code availability

The codes used in this study are publicly available on Zenodo at https://doi.org/10.5281/zenodo.20835638 (ref. ^60^).
