# Supplementary material for: Degree-of-polarization modulation for high-dimensional optical computing
Source: Nature. 2026 Aug 12;657(8131):377–84. doi: 10.1038/s41586-026-10891-z (PMC13558076; doi:10.1038/s41586-026-10891-z)
Supplement: Supplementary file 1 [file 41586_2026_10891_MOESM1_ESM.pdf]

nature portfolio

https://doi.org/10.1038/s41586-026-10891-z

Supplementary information

Degree-of-polarization modulation for high-dimensional optical computing

In the format provided by the authors and unedited

Supplementary Information

Degree-of-polarization modulation for high-dimensional optical computing

Alessandro Petrini,[1,2] Claudio Conti,[1] and Davide Pierangeli[1,3,*]

[1]*Department of Physics, Sapienza University of Rome, 00185 Rome, Italy*

[2]*Department of Basic and Applied Sciences for Engineering, Sapienza University of Rome, 00185 Rome, Italy*

[3]*Institute for Complex Systems, National Research Council, 00185 Rome, Italy*

[*] davide.pierangeli@uniroma1.it

SUPPLEMENTARY NOTE 1. EXPERIMENTAL SETUP

The optical setup is illustrated in Supplementary Fig. 1 and comprises the spatial DOP and SOP modulator, the high-dimensional photonic neural network (PNN) used for color image classification (or, in alternative, the optical encryption system), a polarimeter, a non-full-Stokes polarization camera (or, in alternative, a full-Stokes polarization imaging setup). The laser source is a CW laser (LaserQuantum Ventus, 250 mW) at $\lambda = 532$ nm horizontally (H) polarized, i.e., along the x axis.

As sketched in Supplementary Fig. 1b, the spatial DOP and SOP modulator is composed of a phase-only spatial light modulator (SLM, Hamamatsu X13138, 1280×1024 pixels, 12.5 μm pixel pitch, 60 Hz frame rate) sandwiched between an half-waveplate (HWP) and two output waveplates, a quarter-waveplate (QWP) and HWP equipped with high-speed motorized rotation stages (25°/s rotation velocity), programmed together with the SLM. To operate as a spatial modulator of the DOP and SOP, the SLM is first divided into micromodes made of $l \times l$ pixels, where each pixel is made to operate as a polarization pixel. Specifically, the SLM introduces a phase delay ϕ between the x and y components of the field, which is converted into a polarization modulation through the output waveplates (WPs) set at angles α (HWP) and β (QWP). Considering the Jones matrix of the phase modulator as SLM $= [e^{i\phi}, 0; 0, 1]$, the transmission matrix of the setup $\mathbf{T}(\phi, \alpha, \beta) = \mathrm{HWP}(\alpha)\mathrm{QWP}(\beta)\mathrm{SLM}(\phi)$ at the single-pixel level writes as

$$\mathbf{T} = \begin{bmatrix} \frac{1}{2}(1+i)e^{i\phi}[\cos(2\alpha) - i\cos(2(\alpha-\beta))] & -\frac{1}{2}(1+i)[\sin(2\alpha) + i\sin(2(\alpha-\beta))] \\ -\frac{1}{2}(1+i)e^{i\phi}[\sin(2\alpha) - i\sin(2(\alpha-\beta)] & \frac{1}{2}(1-i)[\cos(2(\alpha-\beta)) - i\cos(2\alpha)] \end{bmatrix}. \quad (1)$$

The field outcoming from the modulator pixel is $\mathbf{E}^{\mathrm{out}} = \mathbf{T}\mathbf{E}^{\mathrm{in}}$, where the input Jones vector is $\mathbf{E}^{\mathrm{in}} = [1/\sqrt{2}; 1/\sqrt{2}]$ as we set the input polarization to diagonal (D) in all the experiments. By tuning the phase ϕ in the interval $[0,\, 2\pi]$ along with α and β, every SLM pixel programs a SOP that can be varied over the entire surface of the Poincaré sphere according to the transmission matrix in Eq. (1) (see Supplementary Note 2).

To control the DOP in space, the SLM active area is further divided into N square macromodes composed of M micromodes with random SOP. The phase of each micromode follows a Gaussian PDF with mean $\bar{\phi}$ in $[0, 2\pi]$ and standard deviation $\delta\phi$ that we keep in the interval $[0, \pi/2]$, as detailed in Methods. We control the state of a macromode within the Poincaré sphere by means of the four experimental parameters ($\bar{\phi}$, $\delta\phi$, α, β), which are

all programmed in real-time during the experiments by a custom code controlling the spatial modulator. DOP and SOP modulation is calibrated using a rotating-waveplate polarimeter (Thorlabs PAX1000VIS, 0.25° nominal accuracy on azimuth and ellipticity). Polarization imaging is performed by two different methods. A non-full-Stokes polarization camera (Thorlabs Kiralux, 2448 $\times$ 2048 pixels), in a separate optical arm, acquires images of the polarization-structured beam through a $4f$ lens system (0.3$\times$ magnification), thereby measuring maps of the linear polarization degree (ν) and the azimuth θ (method 1). Full-Stokes polarization imaging (method 2) is also performed in this optical arm by using the camera in intensity-only mode and inserting along the path a division-of-time analyzer consisting of a rotating polarizer (PL) and a QWP. We perform spatially-resolved Stokes measurements by sequentially acquiring the standard set of intensity projections [1]. These intensity images are processed in order to obtain maps of S_1, S_2, S_3, and the resulting ρ.

When used as a high-dimensional data encoder for optical computing, the DOP modulator is interfaced with the PNN sketched in Supplementary Fig. 1c. This random PNN, implemented in free space, is made by several ($n = 1, ..., 4$) diffusers (Thorlabs N-BK7 ground glass diffusers with 120, 200, 600, or 1500 grit polishes) stacked in series along the optical path and a CMOS intensity camera (Basler a2A1920 160μm PRO, 1920 $\times$ 1200 pixels, 12 bits intensity sensitivity) connected to the digital backend (few-node neural network) detailed in Methods.

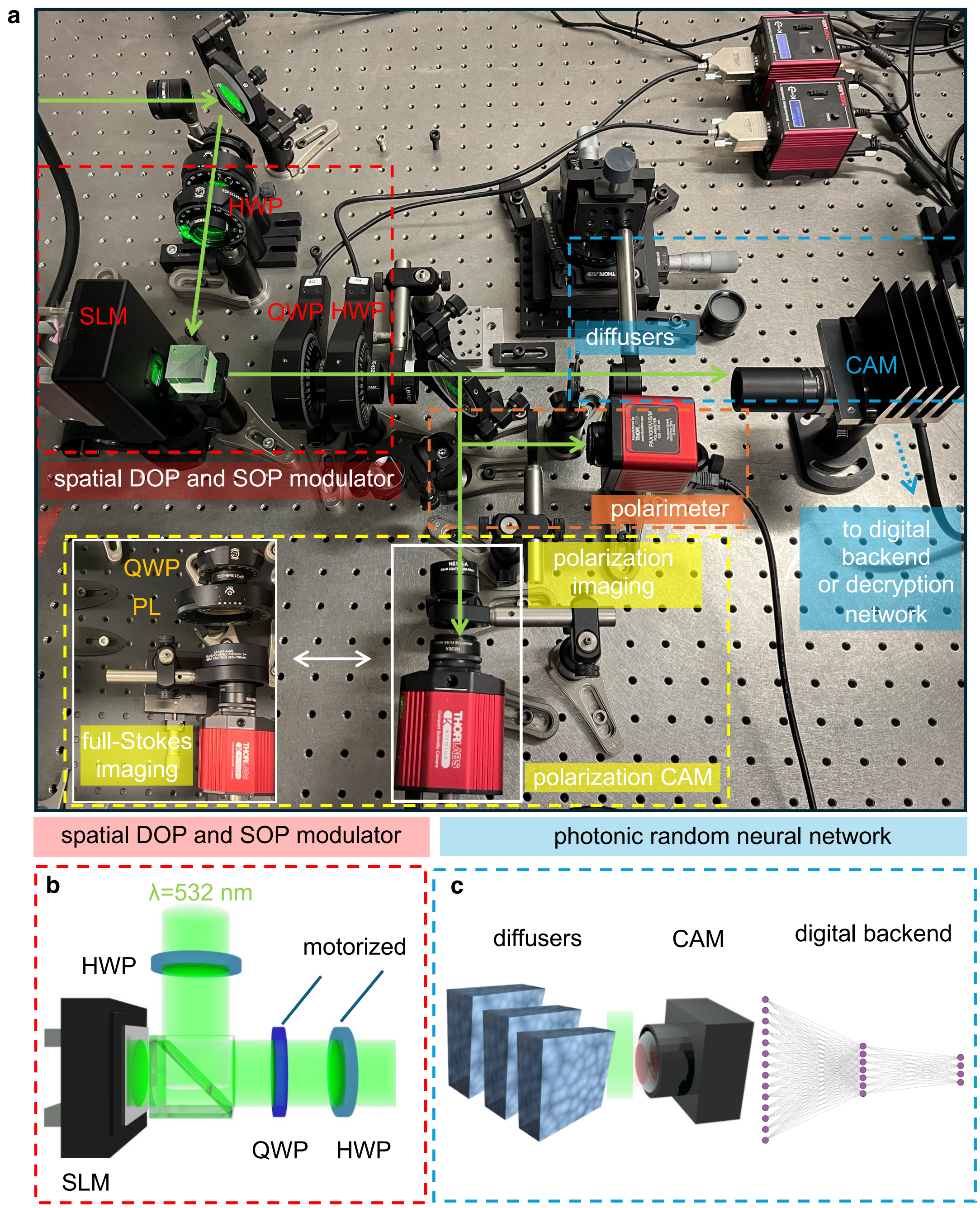

Supplementary Figure 1. **a** Photo of the experimental setup. Laser beam (green arrows), spatial SOP and DOP modulator (red box), polarimeter (orange box), non-full-Stokes polarization camera or full-Stokes imaging (yellow box), and optical neural network (blue box). **b** Illustration of the spatial DOP and SOP modulator. The expanded CW laser ($\lambda = 532$ nm) is diagonally (45°) polarized by an half-waveplate (HWP), reflected by the phase-only spatial light modulator (SLM), and transmitted through a motorized quarter-waveplate (QWP) and HWP. **c** Sketch of the high-dimensional PNN for parallel processing of RGB images encoded in polarization. The digital backend is a few-node fully-connected network (see Methods).

SUPPLEMENTARY NOTE 2. POLARIZATION MATRIX MODEL

The polarization properties of a quasi-monochromatic light beam are described within the coherence theory by the *polarization matrix* $\mathbf{J}$ [2]. Originally referred to as *coherence matrix*, the polarization matrix $\mathbf{J}$ expresses an optical field with arbitrary DOP in terms of the equal-time correlations between its orthogonal components E_x and E_y as

$$\mathbf{J} = \begin{bmatrix} \langle E_x E_x^* \rangle & \langle E_x E_y^* \rangle \\ \langle E_y E_x^* \rangle & \langle E_y E_y^* \rangle \end{bmatrix} = \begin{bmatrix} J_{xx} & J_{xy} \\ J_{yx} & J_{yy} \end{bmatrix}, \tag{2}$$

where the bracket $\langle\rangle$ denotes the time average or, assuming ergodicity, an ensemble average [2]. The matrix $\mathbf{J}$ is Hermitian, $J_{xy} = J_{yx}^*$, and also non-negative definite, which implies the inequality $|J_{xy}| \leq \sqrt{J_{xx}}\sqrt{J_{yy}}$.

To model our DOP modulator according to polarization matrix theory, we explicitly evaluate each term $J_{ij} = \langle E_i E_j^* \rangle$ $(i, j = x, y)$ for the spatial ensemble of polarized waves that composes the macromode and we compute the resulting Stokes vector $\mathbf{S}$ and degree of polarization ρ as a function of the modulator parameters. According to Eq. (1), we write the field outcoming from the modulator as

$$\begin{pmatrix} E_x \\ E_y \end{pmatrix} = \frac{1}{\sqrt{2}} \begin{pmatrix} (1+i)\left(e^{i\phi}[\cos(2\alpha) - i\cos(2(\alpha-\beta))] - \sin(2\alpha) - i\sin(2(\alpha-\beta))\right) \\ -(1+i)\left(\cos(2\alpha) + i\cos(2(\alpha-\beta)) + e^{i\phi}[\sin(2\alpha) - i\sin(2(\alpha-\beta))]\right) \end{pmatrix}, \tag{3}$$

and the four correlation products $E_i E_j^*$ as

$$\begin{aligned} E_x E_x^* &= [1 - \sin(\phi)\sin(4\alpha-2\beta) - \cos(\phi)\cos(4\alpha-2\beta)\sin(2\beta)]/2 \\ E_x E_y^* &= e^{-i\phi}\left[(\cos(2\alpha-2\beta) - i\cos(2\alpha))^2 + e^{2i\phi}(\sin(2\alpha) - i\sin(2\alpha-2\beta))^2\right]/4 \\ E_y E_x^* &= e^{-i\phi}\left[e^{2i\phi}(\cos(2\alpha-2\beta) + i\cos(2\alpha))^2 + (\sin(2\alpha) + i\sin(2\alpha-2\beta))^2\right]/4 \\ E_y E_y^* &= [4 - 2\cos(\phi+4\alpha-2\beta) + 2\cos(\phi-4\alpha+2\beta) - \sin(\phi-4\alpha) + \sin(\phi+4\alpha) \\ &\quad - \sin(\phi+4\alpha-4\beta) + \sin(\phi-4\alpha+4\beta)]/8. \end{aligned} \tag{4}$$

The phase ϕ follows a Gaussian PDF with mean $\bar{\phi}$ and variance $\delta\phi^2$,

$$\mathcal{N}(\phi) = \frac{1}{\sqrt{2\pi\delta\phi^2}} e^{-\frac{(\phi-\bar{\phi})^2}{2\delta\phi^2}}, \tag{5}$$

so that the polarization matrix elements are given by

$$\langle E_i E_j^* \rangle = \int_{-\infty}^{+\infty} E_i E_j^* \mathcal{N}(\phi) d\phi. \tag{6}$$

The integrals in Eq. (6) are analytically computed and give

$$
\begin{aligned}
J_{xx} =& e^{i\psi}\left[4e^{i\bar{\phi}+\delta\phi^2/2} + 2i(e^{2i\bar{\phi}}-1)\sin(4\alpha-2\beta) - 2(e^{2i\bar{\phi}}+1)\cos(4\alpha-2\beta)\sin(2\beta)\right]/8\\
J_{xy} =& e^{i\psi}\left[(\cos(2\alpha-2\beta) - i\cos(2\alpha))^2 + e^{2i\bar{\phi}}(\sin(2\alpha) - i\sin(2\alpha-2\beta))^2\right]/4\\
J_{yx} =& e^{i\psi}\left[e^{2i\bar{\phi}}(\cos(2\alpha-2\beta) + i\cos(2\alpha))^2 + (\sin(2\alpha) + i\sin(2\alpha-2\beta))^2\right]/4\\
J_{yy} =& e^{i\psi}\left[(e^{2i\bar{\phi}}+1)\sin(4\alpha) - (e^{2i\bar{\phi}}+1)\sin(4\alpha-4\beta) + 4e^{i\bar{\phi}}[e^{\delta\phi^2/2} + \sin(2\alpha-2\beta)\sin(\bar{\phi})\right]/8,
\end{aligned}
\tag{7}
$$

where we denote $\psi = -\bar{\phi} + i\delta\phi^2/2$. In terms of the polarization matrix $\mathbf{J}$, the DOP ρ is given by [2]

$$
\rho = \sqrt{1 - \frac{4\mathrm{Det}\mathbf{J}}{(\mathrm{Tr}\mathbf{J})^2}}. \tag{8}
$$

We calculate the determinant $\mathrm{Det}\mathbf{J}$ and trace $\mathrm{Tr}\mathbf{J}$ by using Eq. (7) and, from Eq. (8), we find the analytical solution:

$$
\rho = e^{-\delta\phi^2/2}. \tag{9}
$$

The analytical result reveals the key property enabling DOP control: the DOP does not depend on the mean phase $\bar{\phi}$, nor on the waveplates angles α and β, but only on the the standard deviation $\delta\phi$ of the phase fluctuations. Eq. (9) is experimentally demonstrated in Fig. 2a and Supplementary Fig. 2a. The measured ρ is well fitted by Eq. (9) for any set of parameters $(\bar{\phi}, \alpha, \beta)$.

We thus calculate the Stokes parameters at the single-pixel level as

$$
S_i = \mathrm{Tr}(\sigma_i \varrho), \tag{10}
$$

where $\varrho = EE^\star$ [Eq. (4)] and σ_i are the Pauli matrices. We obtain

$$
\begin{aligned}
S_1(\phi,\alpha,\beta) =& [\cos(\phi+4\alpha-2\beta) - \cos(\phi-4\alpha+2\beta) - 2\cos(\phi)\cos(4\alpha-2\beta)\sin(2\beta)]/2\\
S_2(\phi,\alpha,\beta) =& \cos(\phi)\sin(4\alpha-2\beta)\sin(2\beta) - \cos(4\alpha-2\beta)\sin(\phi)\\
S_3(\phi,\alpha,\beta) =& \cos(\phi)\cos(2\beta),
\end{aligned}
\tag{11}
$$

which describe the orbit traced on the Poincaré sphere when varying ϕ at fixed α and β. Eq. (11) also models the SOP of the macromode with $\phi = \bar{\phi}$. The Stokes parameters of the macromode are given by

$$
S_i = \int_{-\infty}^{+\infty} S_i(\phi,\alpha,\beta)\mathcal{N}(\phi)d\phi, \tag{12}
$$

whose solution yields

$$S_i = e^{-\delta\phi^2/2} S_i(\bar{\phi}, \alpha, \beta) = \rho S_i(\bar{\phi}, \alpha, \beta). \tag{13}$$

According to Eq. (13), the polarized component of the field in the macromode is independent of $\delta\phi$. Therefore, the SOP and DOP resulting from a spatial distribution of phases with Gaussian PDF are independent, as experimentally demonstrated in Fig. 2. The modulation of S_1, S_2, and S_3 measured as a function of $\bar{\phi}$ strictly follows Eq. (11), as shown in Fig. 2b.

SUPPLEMENTARY NOTE 3. CONTROLLING THE DOP

Figure 2 reports the measured ρ for a single random realization of the phase mask as a function of $\delta\phi$. Here, we investigate the dependence on the specific realization of the phase mask, demonstrating that our statistical method for controlling the DOP is independent of the specific instance drawn for the programmed PDF. We measure ρ for ten different phase masks extracted by using the same Gaussian PDF (i.e., the same $\delta\phi$ and $\bar{\phi}$) at every $\delta\phi$. Supplementary Fig. 2a reports the mean value of ρ varying $\delta\phi$ at constant $\bar{\phi} = \pi$. Supplementary Fig. 2b shows the different phase masks, composed of $M = 256$ micromodes, used for the data point at $\delta\phi = 0.23$. Supplementary Fig. 2c reports the measured state inside the Poincaré sphere for two realizations of the phase mask. This illustrates how different beam realizations correspond to the same state within the experimental uncertainty. Averaging the measured value of ρ over a set of instances, we observe a smooth dependence on $\delta\phi$, with strongly reduced noise compared to single-mask measurements (Fig. 2a). This allows us to calibrate accurately the experimental relation between ρ and $\delta\phi$ according to the fitting function $\rho = a\exp(-b\delta\phi^2) + c$, with $a = 0.86$, $b = 0.52$, $c = 0.14$, further improving the fidelity of the DOP modulator.

We study in detail the dependence of the DOP on the parameters of the phase mask. In particular, Supplementary Fig. 3a reports the mean ρ versus $\delta\phi$ measured by using phase masks with a different number of micromodes M. As shown in Supplementary Fig. 3b, the phase masks at every $\delta\phi$ share the same PDF $\mathcal{N}(\bar{\phi}, \delta\phi)$ but differ in M, and, consequently, in the physical size of the micromode (side length l). The side length L of the single macromode is fixed to $L = l \times \sqrt{M} \approx 10^3$ pixels (12.5 mm) in this analysis. The DOP weakly depends on M at small values of $\delta\phi$, while a more pronounced effect on the modulation curve is observed at large $\delta\phi$, with the generated beams that retain a significant polarized component. This

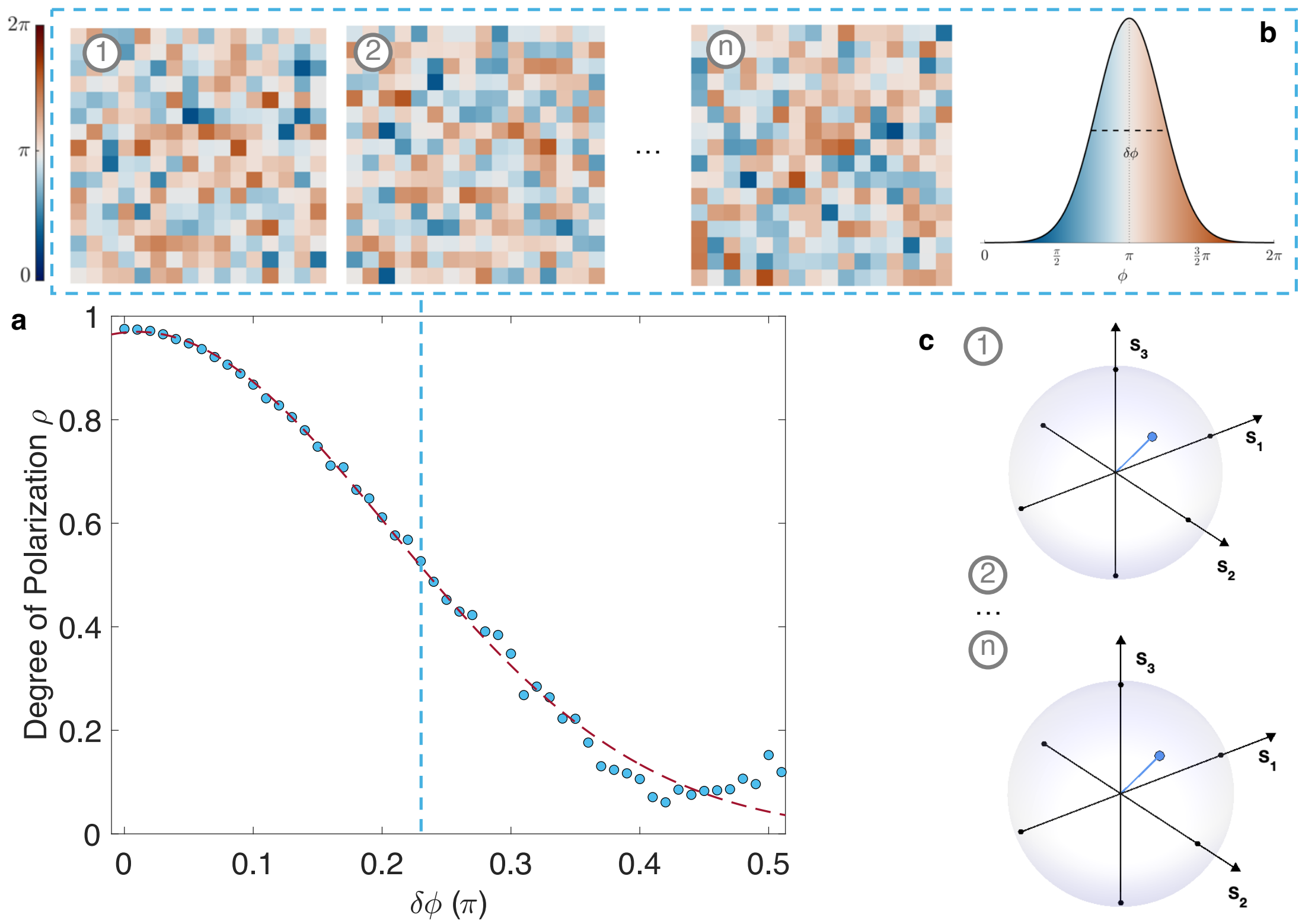

Supplementary Figure 2. DOP from replicated phase masks. **a** Measured mean value of ρ versus $\delta\phi$ fitted by the theoretical relation Eq. (9). Each data point is obtained by measuring the DOP of ten random instances. **b** Example of phase masks generated from the same PDF ($\delta\phi = 0.23$, $\bar{\phi} = \pi$). **c** Measured states inside the Poincaré sphere corresponding to the mask realizations in **b**.

indicates that, experimentally, the phase microstructure partially washes out for $M \approx 10^5$ due to the strong diffraction of the micromode immediately after the SLM plane, resulting in an average phase and making the method less effective under such a condition. On the other hand, the ρ value slightly smaller than 1 observed for $\delta\phi = 0$ (homogeneous mask), is due to a minor depolarization effect induced by the SLM. Due to these experimental effects, M plays a relevant role in practice and can be used to control the DOP modulation depth $\max(\rho) - \min(\rho)$ (Supplementary Fig. 3c). A maximum modulation depth of 0.93 is obtained around $M = 3600$. This optimal working point holds for a single macromode of large size ($L = 10^3$ pixels). However, the analysis in Supplementary Fig. 3 indicates that DOP modulation operates very accurately already at $M = 100$, with near-optimal performance for a broad range of M values, which indicates the robustness and scalability of our statistical method to control the DOP.

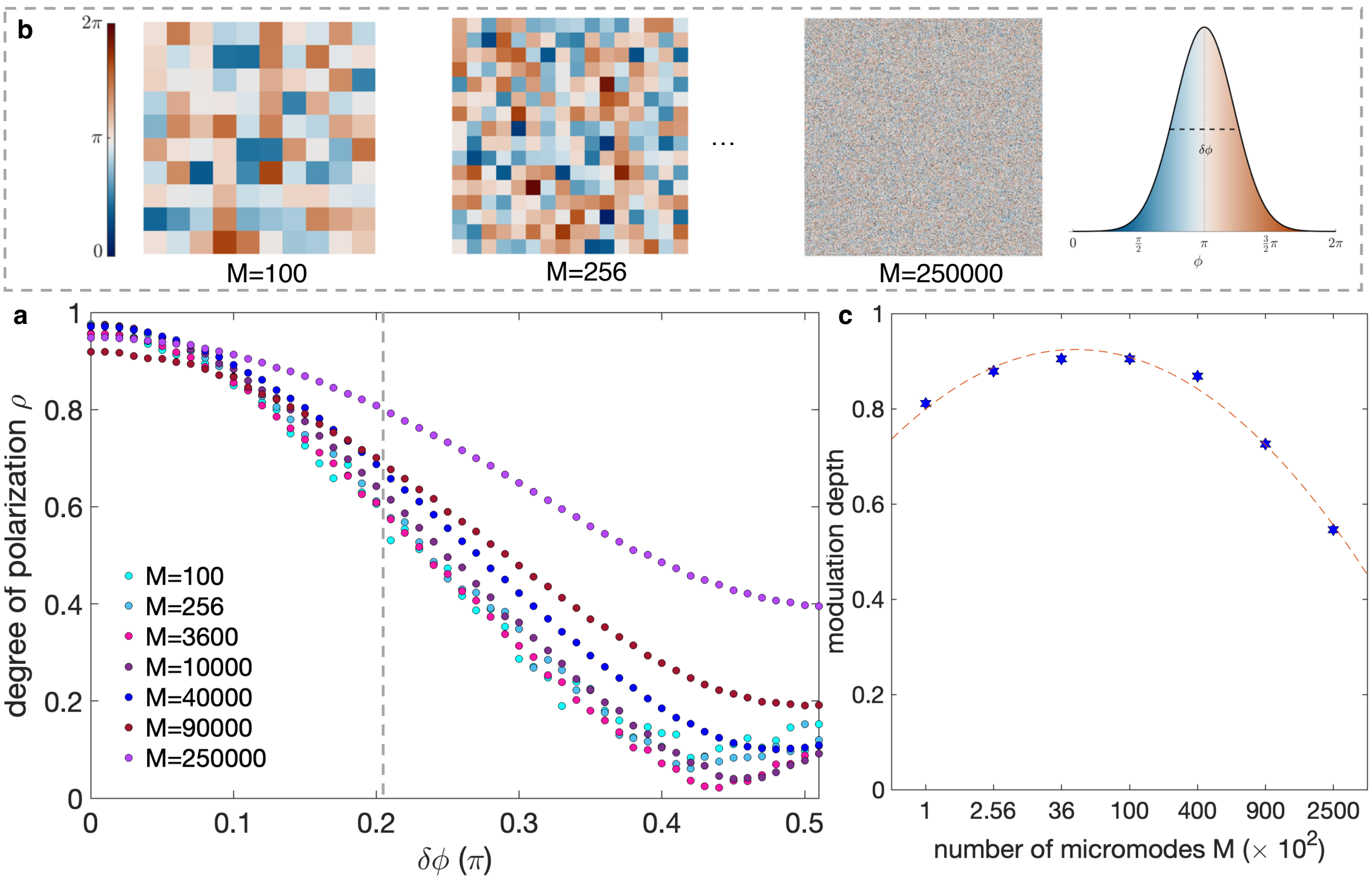

Supplementary Figure 3. Effect of the number of micromodes on the DOP. **a** Mean ρ as a function of $\delta\phi$ for phase masks with different M. **b** Example of phase masks with increasing M drawn from same PDF a fixed $\delta\phi$ (dashed line). **c** DOP modulation depth as a function of M.

SUPPLEMENTARY NOTE 4. TWO-SLM IMPLEMENTATION

In the setup made by a single SLM and WPs (Supplementary Fig. 1), the possible SOPs are constrained over an ellipse on the Poincaré sphere, which can be varied by the waveplate angles according to Eq. (11). This limits the possible SOPs achievable across multiple macromodes within a single structured beam at a fixed configuration of the WPs. To overcome this limitation, we also implement our method by using two precisely aligned SLMs and no rotating WPs. This improved configuration replaces the static rotation of the Poincaré sphere performed via the angles α and β with an additional spatially-tunable phase delay, making the spatial DOP and SOP modulator fully programmable. In this implementation, every macromode is programmed independently of the others and all possible polarization states can be spanned within a single modulated beam.

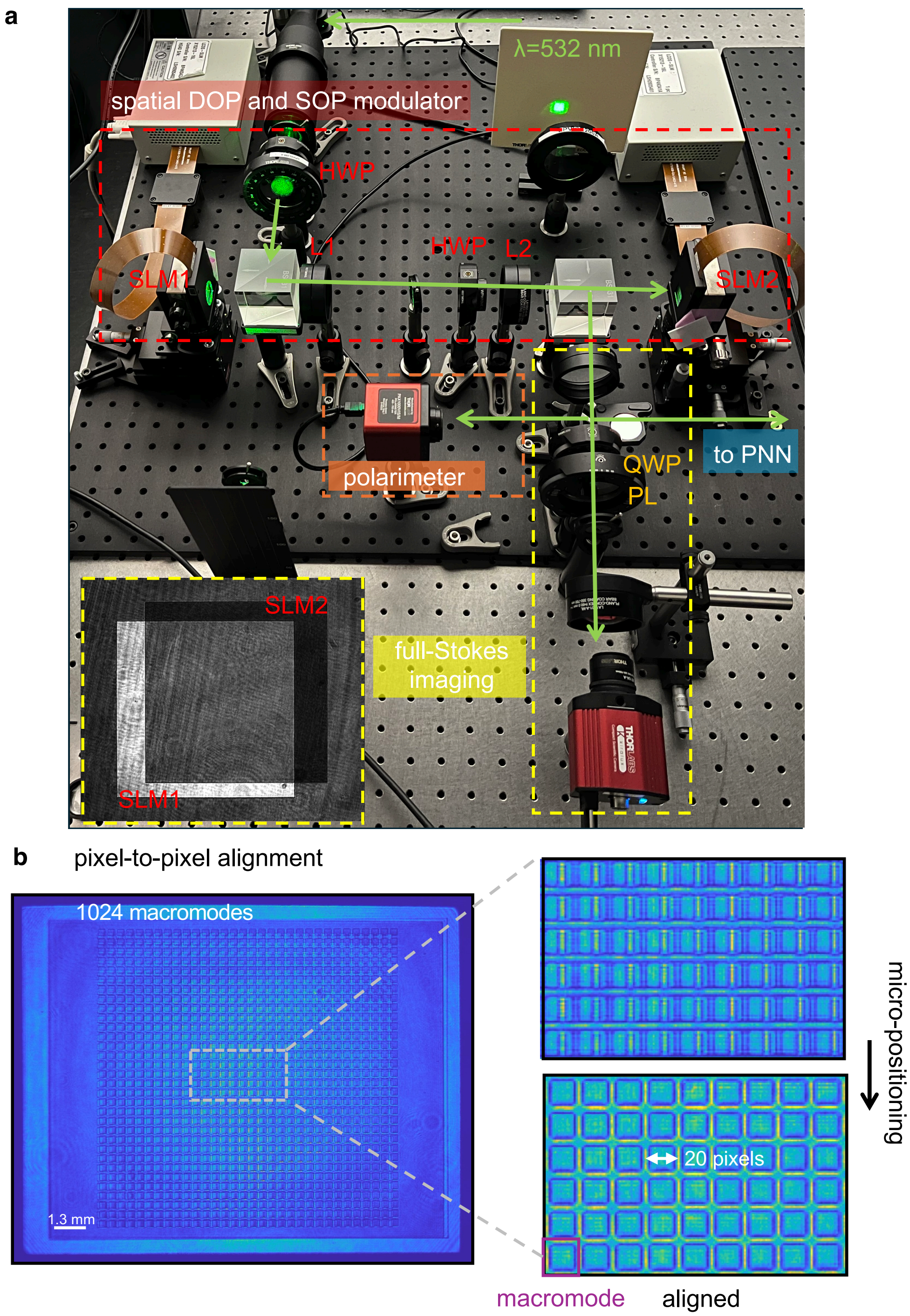

Supplementary Figure 4. **a** Photo of the two-SLM setup. Laser beam (green arrows), spatial DOP and SOP modulator (red box), polarimeter (orange box), and full-Stokes imaging (yellow box). The SLM1 is imaged on the second one (SLM2) through a $4f$ system (lenses L1 and L2) and a HWP. The inset shows the intensity through a polarizer of two square modes of different polarization modulated by the two SLMs as observed on the output optical plane for alignment purposes. **b** Pixel-to-pixel alignment. Patterns made by 32×32 well-separated square modes of side length 20 pixels are created on the two SLMs and are aligned at the single-pixel resolution (± 12.5 μm) by precision multi-axis stages.

The two-SLM setup is shown in Supplementary Fig. 4a. Two identical phase-only SLMs (Hamamatsu X15213-16L, 1272×1024 pixels, 12.5 μm pixel pitch, 15.9×12.8 mm effective area, 256 phase levels) operate in series on the expanded input beam (Coherent Sapphire SF NX 532) linearly polarized at 45°(diagonal, D). The optical plane of the first modulator (SLM1) is imaged with unit magnification on the plane of the second one (SLM2) by a $4f$ system made by two plano-convex lenses with focal length $f = 100$ mm (numerical aperture NA ≈ 0.25). A HWP at a fixed angle $\gamma = 22.5°$ is placed in-between the two SLMs in order to rotate the SOP produced by the SLM1 along the modulation axis of the SLM2. The two SLMs are aligned pixel-to-pixel with an accuracy of one pixel (± 12.5 μm). This high-precision alignment procedure is illustrated in Supplementary Fig. 4b. An alignment pattern consisting of 32×32 square modes of constant phase is displayed on both SLMs. These modes have a side length of 20 pixels and are separated by 6 blank pixels. The SLM2 plane is imaged on the camera through a polarizer and the intensity image is used to precisely tune the position of the two SLMs via precision 5-axis translation and rotation stages. This procedure ensures that macromodes on the two SLMs overlap almost perfectly (Supplementary Fig. 4b) even at the smallest macromode size that we employ.

The DOP and SOP modulator realized with two SLMs operates in close analogy with the scheme made by a single SLM and WPs. We denote ϕ_1 and ϕ_2 the phase on the first and second SLM, respectively. The two phases directly map two angles along two orthogonal directions on the Poincaré sphere, enabling complete and independent control of the SOP outcoming from each pixel. Specifically, the transmission matrix of the two-SLM setup $\mathbf{T}(\phi_1, \phi_2, \gamma) = \text{SLM2}(\phi_2)\text{HWP}(\gamma)\text{SLM1}(\phi_1)$ at the single-pixel level is

$$\mathbf{T} = \begin{bmatrix} e^{i(\phi_1+\phi_2)}\cos(2\gamma) & 2e^{i\phi_2}\cos(2\gamma)\sin(2\gamma) \\ 2e^{i\phi_1}\cos(2\gamma)\sin(2\gamma) & -\cos(2\gamma) \end{bmatrix}, \tag{14}$$

which gives the output field

$$\begin{pmatrix} E_x \\ E_y \end{pmatrix} = \frac{1}{\sqrt{2}} \begin{pmatrix} e^{i\phi_2}[e^{i\phi_1}\cos(2\gamma) + \sin(2\gamma)] \\ e^{i\phi_1}\sin(2\gamma) - \cos(2\gamma) \end{pmatrix}. \tag{15}$$

To spatially modulate the DOP and SOP, both SLMs are divided into N square macromodes made of $L \times L$ pixels. The division of each macromode into M micromodes ($l \times l$ pixels) with random phases from a Gaussian PDF $\mathcal{N}^{(i)}(\phi_1)$ is applied only on the SLM1. Macromodes on SLM2 are blocks of constant phase $\phi_2^{(i)}$. For the single macromode, the DOP is programmed

by the standard deviation $\delta\phi_1$ and the SOP by both the mean phase $\bar{\phi_1}$ and the constant phase ϕ_2. In fact, repeating the polarization matrix treatment in Supplementary Note 2 by starting from Eq. (15), we analytically find:

$$\rho = e^{-\delta\phi_1^2/2}. \tag{16}$$

Eq. (16) coincides with Eq. (9), demonstrating the equivalence of the two implementations for controlling the DOP via polarization statistics. Fixing the HWP angle at $\gamma = \pi/8$ as in the experimental setup, the Stokes parameters of the polarized component are

$$\begin{aligned} S_1(\bar{\phi_1}, \phi_2) &= \cos(\bar{\phi_1}) \\ S_2(\bar{\phi_1}, \phi_2) &= \sin(\bar{\phi_1}) \sin(\phi_2) \\ S_3(\bar{\phi_1}, \phi_2) &= \sin(\bar{\phi_1}) \cos(\phi_2). \end{aligned} \tag{17}$$

Therefore, the entire volume of the Poincaré sphere is now spanned by the three experimental parameters $(\delta\phi_1, \bar{\phi_1}, \phi_2)$ that are programmed independently in every macromode.

In Supplementary Fig. 5, we demonstrate the two-SLM modulator for controlling the DOP and SOP of a single spatial mode. As for Fig. 2, the generated partially-polarized beams are analyzed with the polarimeter. The ρ values are obtained by averaging over ten replicated phase masks with $M = 256$ micromodes of size $l = 64$ pixels. Supplementary Fig. 5a reports ρ at different $\delta\phi_1$ for fixed $\bar{\phi_1} = 0$, $\phi_2 = 0$ ($S_1/\rho = 1$). The DOP modulation is well fitted by the exponential function $\rho = a\exp(-b\delta\phi^2) + c$, with $a = 0.95$ and $b = 0.55$, in close agreement with theory. Control of the SOP at a constant ρ is reported in Supplementary Fig. 5b by scanning both the phases $\bar{\phi_1}$ and ϕ_2 in $[0, 2\pi]$. The measured surfaces in Supplementary Fig. 5b, which characterizes the Stokes parameters S_i as a function of $\bar{\phi_1}$ and ϕ_2, closely agree with Eq. (17). Based on these calibration curves, Supplementary Fig. 5c reports the generation of arbitrary states inside the Poincaré sphere. In comparison to the results in Fig. 2c, we achieve an improved accuracy since the experimental error associated with the mechanical movement of the WPs is eliminated in the two-SLM implementation. The mean errors of this improved realization of the modulator are $4 \pm 1\%$, $2 \pm 1^\circ$, and $2 \pm 1^\circ$, on ρ, θ, and χ, respectively.

The two-SLM implementation allows us to achieve arbitrary SOP control per pixel, overcoming the constraint in programming the SOP of multiple macromodes that occurs by using a single SLM. Supplementary Fig. 6 shows that all possible SOPs can be realized across the

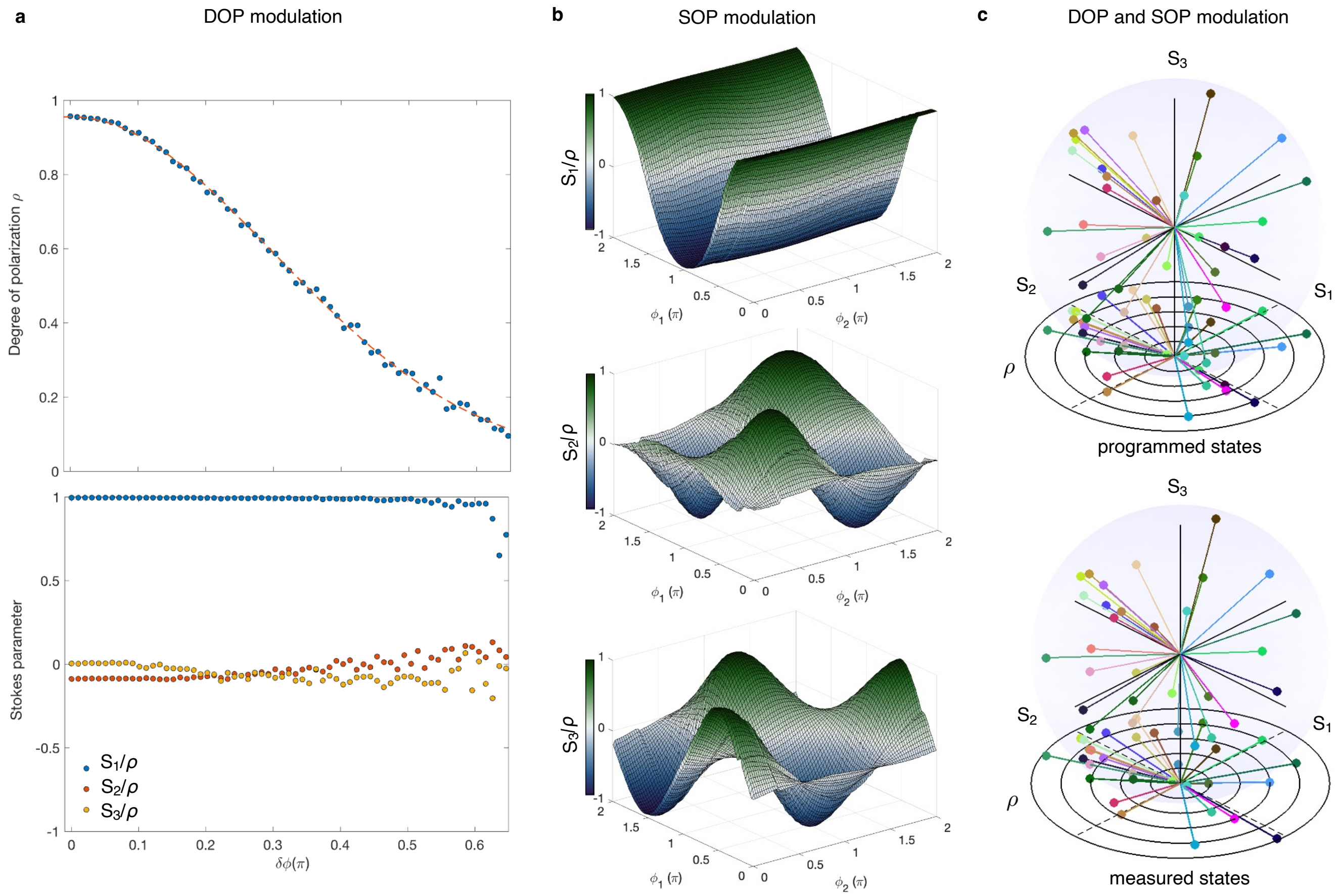

Supplementary Figure 5. DOP and SOP control with two phase-only SLMs. **a** DOP modulation. Measured ρ (dots) and exponential fit (line) according to Eq. (16) by varying $\delta\phi_1$ for $\bar{\phi}_1 = 0, \phi_2 = 0$. **b** SOP modulation. Experimental polarimetry results when scanning $\bar{\phi}_1$ and ϕ_2 at a constant $\delta\phi_1 = 0.1$. The S_1, S_2, and S_3 surfaces follow Eq. (17). **c** Controlled sampling of the solid Poincaré sphere. Measured states in comparison (same colors) with the states that are programmed on the two-SLM modulator.

macromodes of a single beam. As an example of a fully-programmable polarization pattern, we report full-Stokes imaging of a 16×16 mosaic with random SOPs that cover the entire surface of the Poincaré sphere.

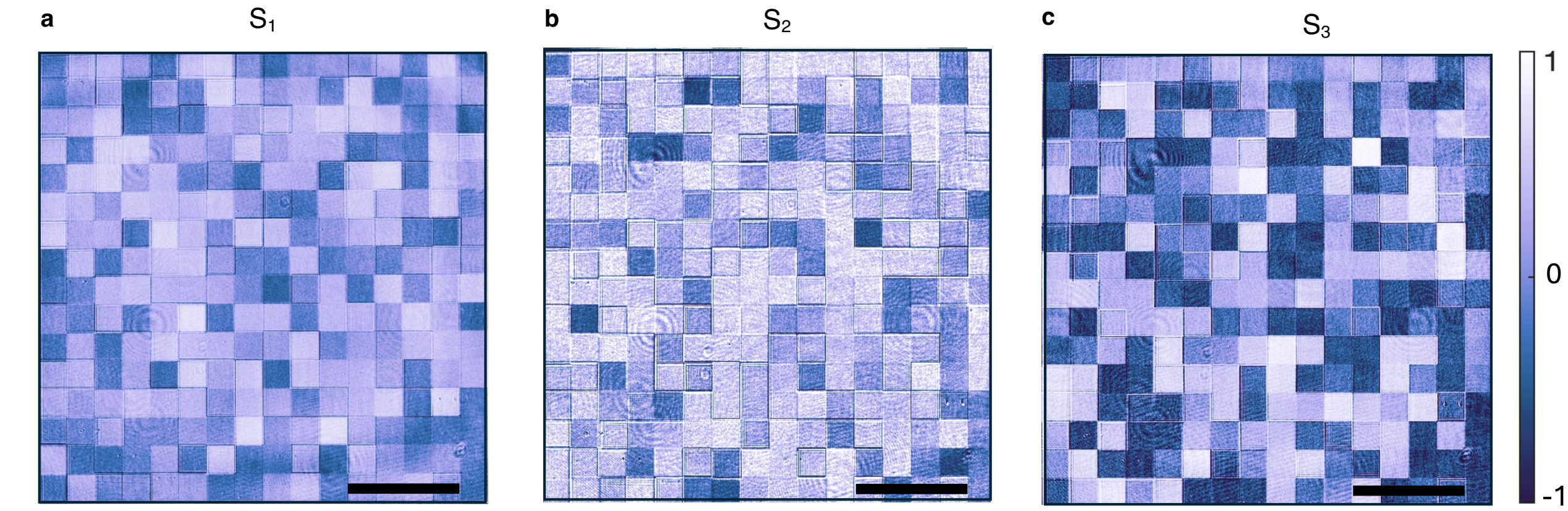

Supplementary Figure 6. Fully-programmable spatial SOP modulation. Measured Stokes maps **a** S_1, **b** S_2, and **c** S_3 for a SOP modulated beam with 16×16 macromodes of random SOPs that cover the surface of the Poincaré sphere with no constraint. Scale bars are 3 mm.

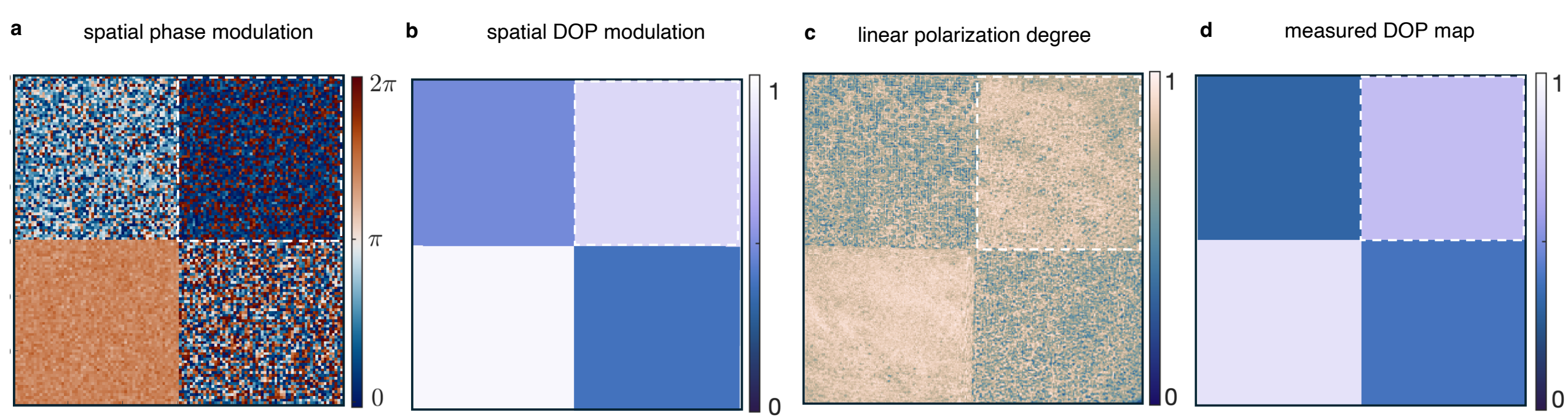

Supplementary Figure 7. Spatial DOP modulation measured by a non-full-Stokes polarization camera (method 1). **a** Phase mask used to generate a beam with $N = 4$ macromodes with different DOP and linear SOP, and **b** corresponding programmed DOP modulation. **c** Measured image of the linear polarization degree ν performed by the non-full-Stokes polarization camera and **d** corresponding spatially-averaged values of DOP.

SUPPLEMENTARY NOTE 5. DOP AND SOP IMAGING

To analyze beams with spatially modulated DOP, we use both non-full-Stokes (method 1) and full-Stokes (method 2) polarization imaging. A DOP image can be obtained also by the non-full-Stokes polarization camera but only when the polarized component of the pattern

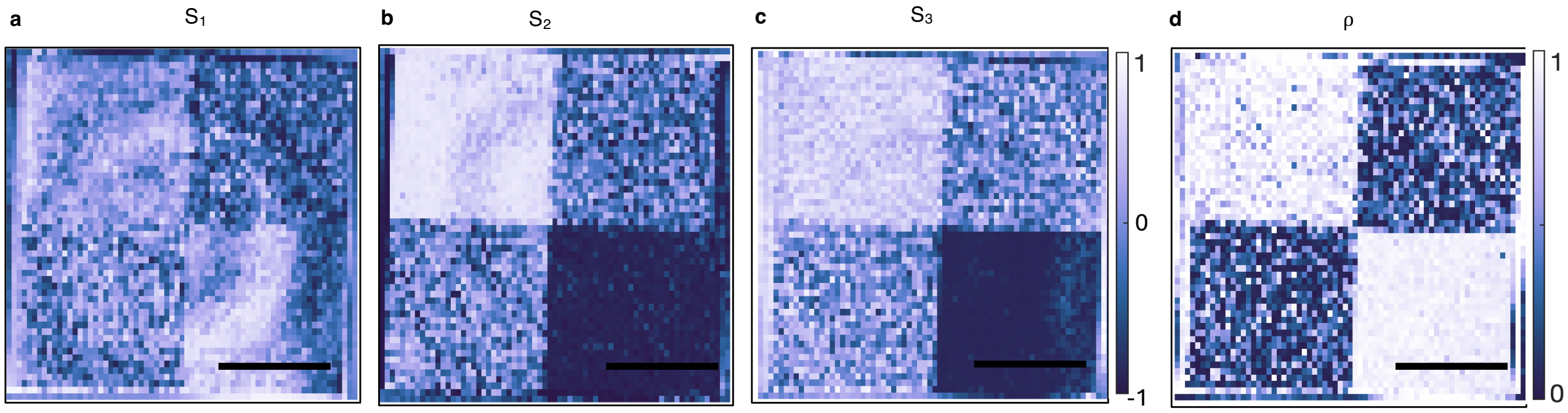

Supplementary Figure 8. Full-Stokes polarization imaging (method 2) of a beam with DOP and SOP modulated in space. Measured Stokes maps **a** S_1, **b** S_2, **c** S_3, and **d** ρ, for the beam in Fig. 3d. Scale bars are 3 mm.

is composed only of linearly polarized light. In fact, when $S_3 = 0$, the linear polarization degree $\nu = \sqrt{S_1^2 + S_2^2}/S_0$ measured by the camera coincides with ρ. This case is relevant since provides a validation of the programmed spatial DOP modulation through an off-the-shelf polarization instrument. We report an experimental measurement in this condition in Supplementary Fig. 7. We program a polarization structured beam with $N = 4$ and $M = 3600$. The employed phase mask is shown in Supplementary Fig. 7a and corresponds to the expected DOP pattern in Supplementary Fig. 7b. The measured image of the linear polarization degree ν in Supplementary Fig. 7c shows the DOP pattern. The microstructure appears as small-scale noise on macromodes of low DOP. The mean value of ρ measured in each block is shown in Supplementary Fig. 7d. The measured DOP is in good agreement with the programmed modulation, with a mean error $\Delta\rho = (1/N)\sum_N(\rho^{\text{meas}} - \rho^{\text{prog}}) = 0.05$. For completeness, Supplementary Fig. 8 reports full-Stokes imaging of the DOP-structured beam shown in Fig. 3c. The ρ map is obtained from the maps of S_1, S_2, and S_3. The result also validates the spatial modulation of the circular polarization component that cannot be detected by the polarization camera.

In Supplementary Fig. 9, we report full-Stokes imaging of a beam composed of $N = 32\times32$ macromodes programmed according to a 4-bit RGB image of 32×32 pixels colored randomly from a set of colors. The Stokes maps (encoding to the three color channels according to Fig. 4a) are recombined via the inverse transformation to give the polarization-colored image in Supplementary Fig. 9b, which is in good agreement with the programmed RGB colors.

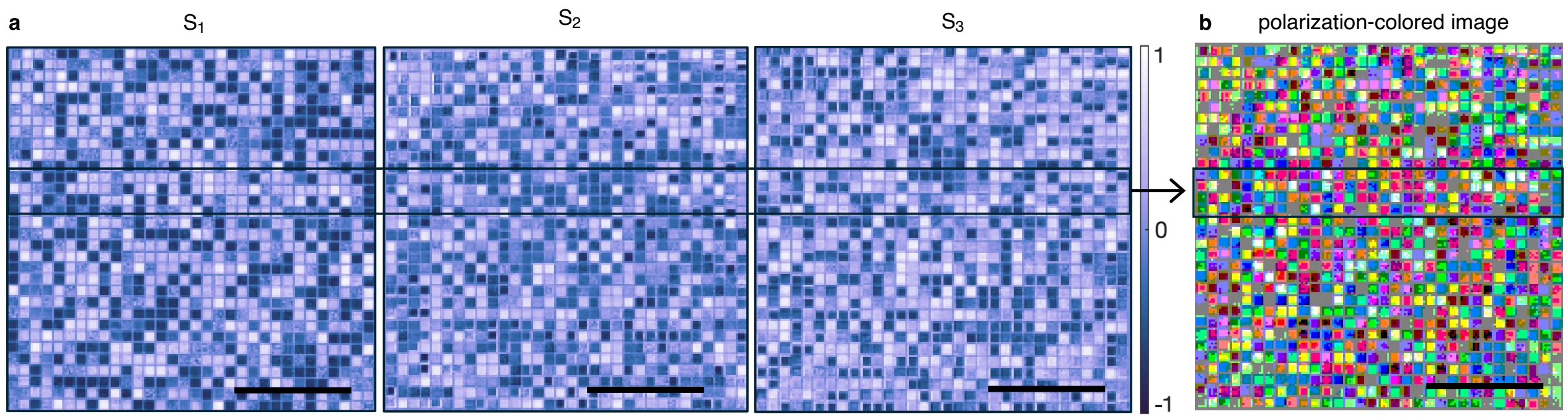

Supplementary Figure 9. Colors encoded in SOP and DOP. **a** Measured Stokes maps for a beam encoding a 32×32 4-bit RGB image with random colors. **b** Polarization-colored image as obtained by applying the inverse color mapping to the measured Stokes maps. Scale bars are 3 mm.

SUPPLEMENTARY NOTE 6. SPATIAL DOP AND SOP MODULATION IN THE FOURIER PLANE

Different applications may require the target beam at a designed location and macromodes of specific size. In cases where focused light is needed, such as for nonlinear optics applications, the DOP and SOP modulated beam can also be realized in the Fourier plane, always by engineering the statistics of the phase modulation on the SLM plane. In this case, the mixing of the micromodes occurs through the operation of a converging lens, which combines the spatial ensemble of SOPs into a focused spot of controlled DOP and SOP. The coherent sum over the micromodes performed by the lens takes the place of the spatial average performed by the finite-area detector on the far-field beam achieved by free-space propagation. In fact, when a single macromode is focused and measured by the polarimeter in the Fourier plane, we obtain a Gaussian modulation of ρ as a function of $\delta\phi$, analogous to the curve in Supplementary Fig. 2. The two realizations are conceptually equivalent, the Fourier-plane modulation can be preferable if the target application requires light to be focused. To demonstrate spatial DOP modulation in this configuration, we employ an array of microlenses. Each microlens operates precisely on a single macromode of the SLM, thus avoiding any possible crosstalk between neighboring macromodes. In the Fourier plane of the microlenses, the field distribution appears as an array of intensity spots with programmable DOP and SOP, where each spot corresponds to a macromode. The

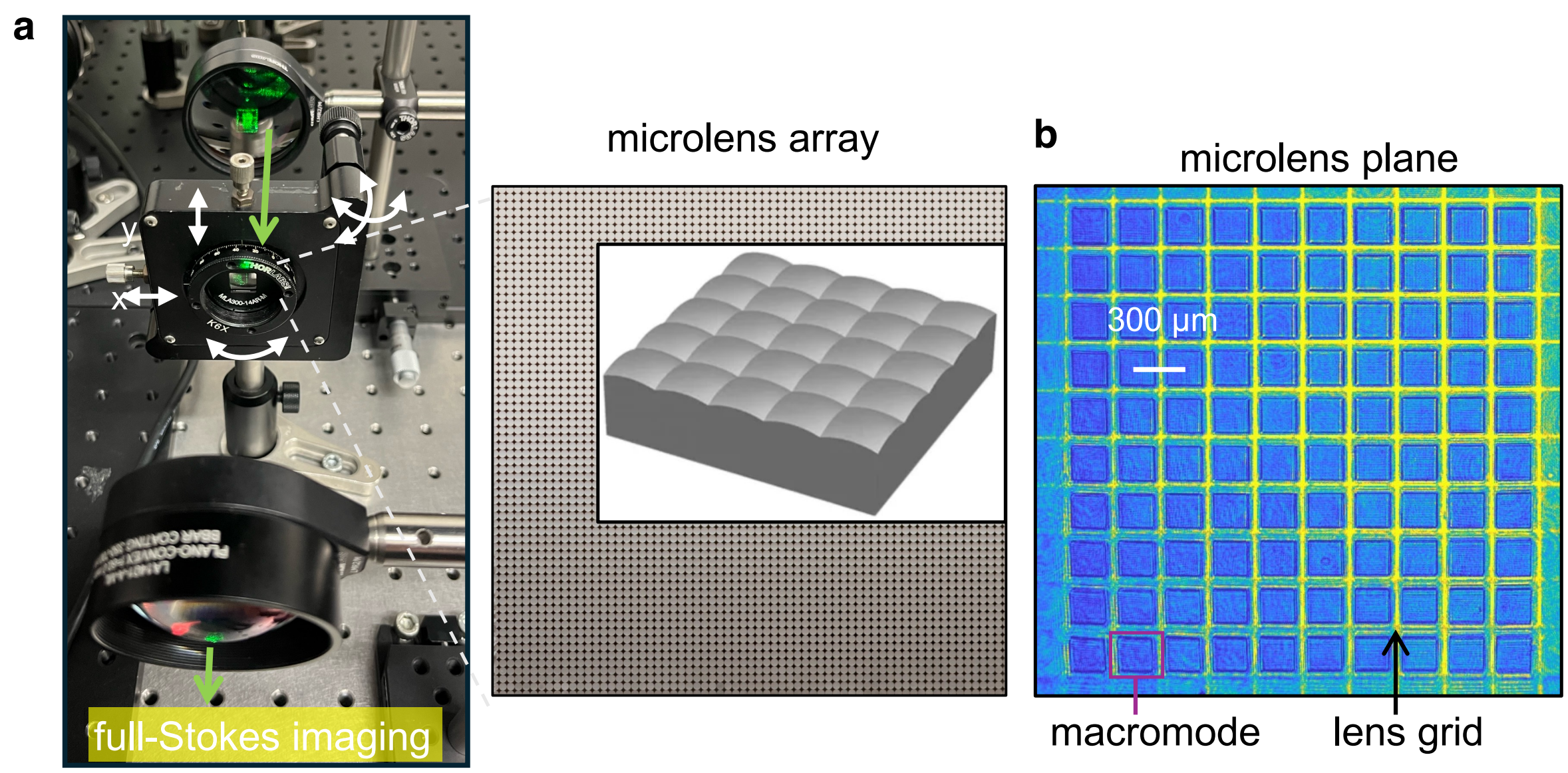

Supplementary Figure 10. Microlens array setup. **a** Photo of the experimental module showing the beam from the SLM relayed on the fused silica microlens array made of square lenses with 300 μm lenslet pitch (inset) hosted by a precision mounting. **b** Intensity image of the microlens plane showing the precise alignment of an array of 10×10 macromodes with the microlenses.

intensity pattern reminds spatial intensity modulation as obtained routinely with SLMs by using manufacturer-provided phase holograms, where the target intensity image forms in the Fourier plane as a set of distinct intensity spots.

The experimental implementation by the microlens array is illustrated in Supplementary Fig. 10a. We use a fused silica microlens array (Thorlabs MLA300-14AR-M) made of square plano-convex lenses of size 295 μm and focal length $f = 14.2$ mm that produces near diffraction-limited spots. The array is 9 mm $\times$ 9 mm sized with a lenslet pitch of 300 μm (30×30 microlenses, fill factor 96.7%). The size and spacing of the macromodes is now set by the lenslet pitch. The SLM plane is relayed by a $4f$ system on the microlens array plane with a magnification factor of 0.4 in order to fit the lenslet pitch. The array is positioned via a precision mounting that allows micrometric translation, rotation, pitch, and yaw. High-precision alignment of the macromode pattern on the lens array is shown in Supplementary Fig. 10b. This intensity image of the microlens plane reveals a calibration pattern of well-separated macromodes of uniform polarization that precisely enters in every lens. On the SLM, the macromodes have a size of $L = 50$ pixels and a spacing of 10 pixels. After the

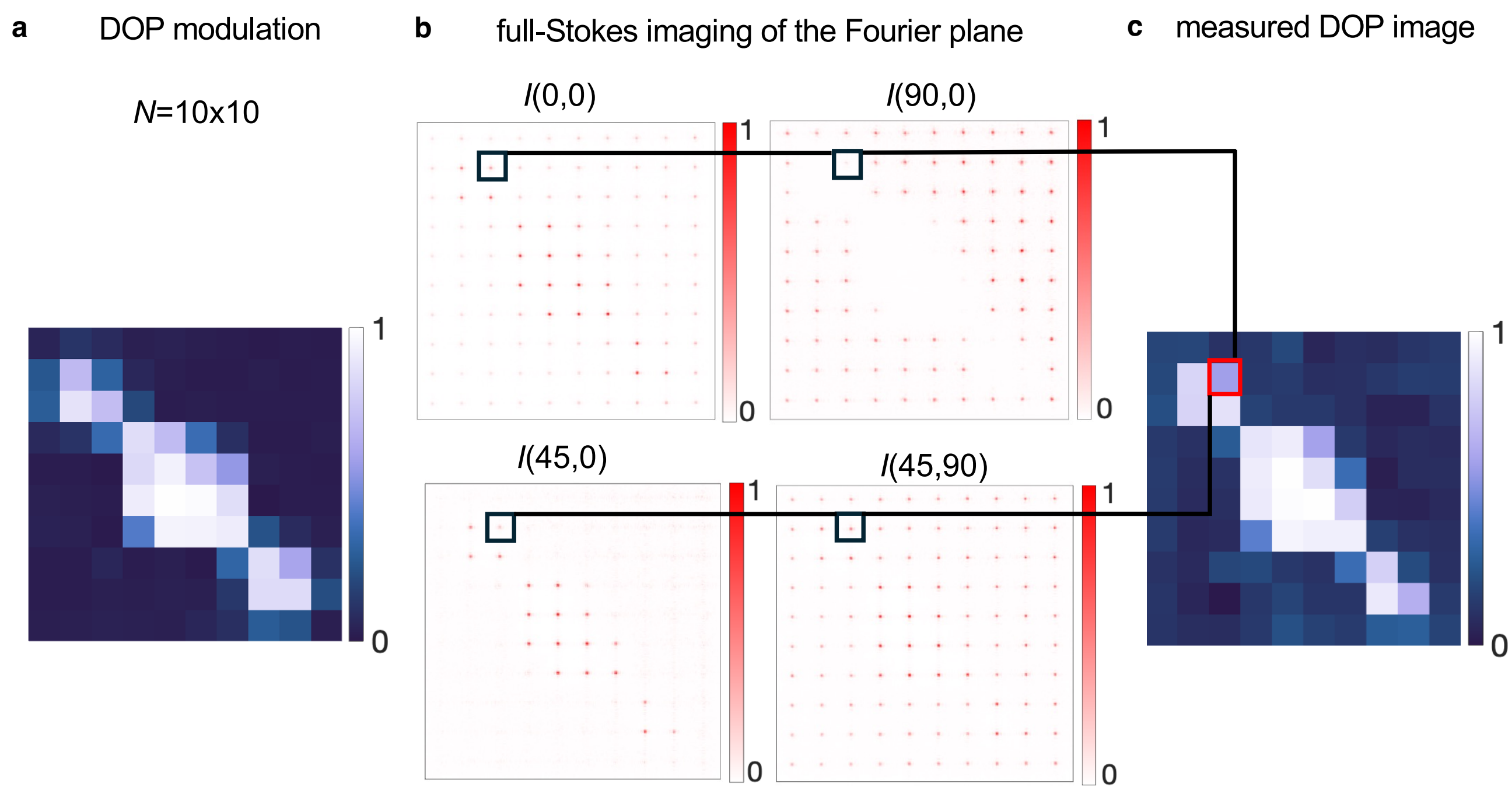

Supplementary Figure 11. Spatial DOP modulation in the Fourier plane. **a** The programmed modulation is a 8-bit grayscale image with 100 pixels resolution. **b** Measured intensity images $I(\theta_{\mathrm{P}}, \theta_{\mathrm{QWP}})$ of the microlens Fourier plane, where θ_{P} and θ_{QWP} indicate the angle of the polarizer and QWP of the full-Stokes analyzer. **c** DOP image obtained by measuring the Stokes parameters of each spot (boxed region) from the intensity projections in **b**.

alignment, we perform full-Stokes measurement of beams structured in DOP and SOP by imaging the Fourier plane.

Spatial DOP modulation via the microlens array is demonstrated in Supplementary Fig. 11. The grayscale image of a candy is programmed within the DOP of $N = 10 \times 10$ macromodes with constant SOP. The four intensity projections in Supplementary Fig. 11b show the array of modulated focal points. The different polarization components show intensity variations across the focal array that indicate the presence of the candy picture. We measure the Stokes parameters of each spot by averaging the intensities over a square region around each focus. We obtain 10×10 values of ρ, which form the map reported in Supplementary Fig. 11c. The measured DOP image reveals the details of the programmed DOP modulation with high accuracy. The result demonstrates how images with 8-bit precision can be encoded with high fidelity by the DOP pattern in the Fourier plane. The structure

made of focal points makes the encoded information readily accessible since every pixels of the target image now corresponds to a focal point.

The encoding of RGB images by spatial DOP and SOP modulation (Fig. 4) also applies in the microlens configuration by leveraging the same one-to-one mapping between the color vector and the Stokes parameters. In this case, each RGB pixel transfers into a polarization-colored focal point. Supplementary Fig. 12 demonstrates the encoding of a multicolor pattern in the Fourier plane. The observation of the encoded RGB data is performed by full-Stokes imaging of the focal array (Supplementary Fig. 12b). By discretizing the Stokes parameters measured for each focus, we obtain the colored image shown in Supplementary Fig. 12, which coincides with the encoded data with no error.

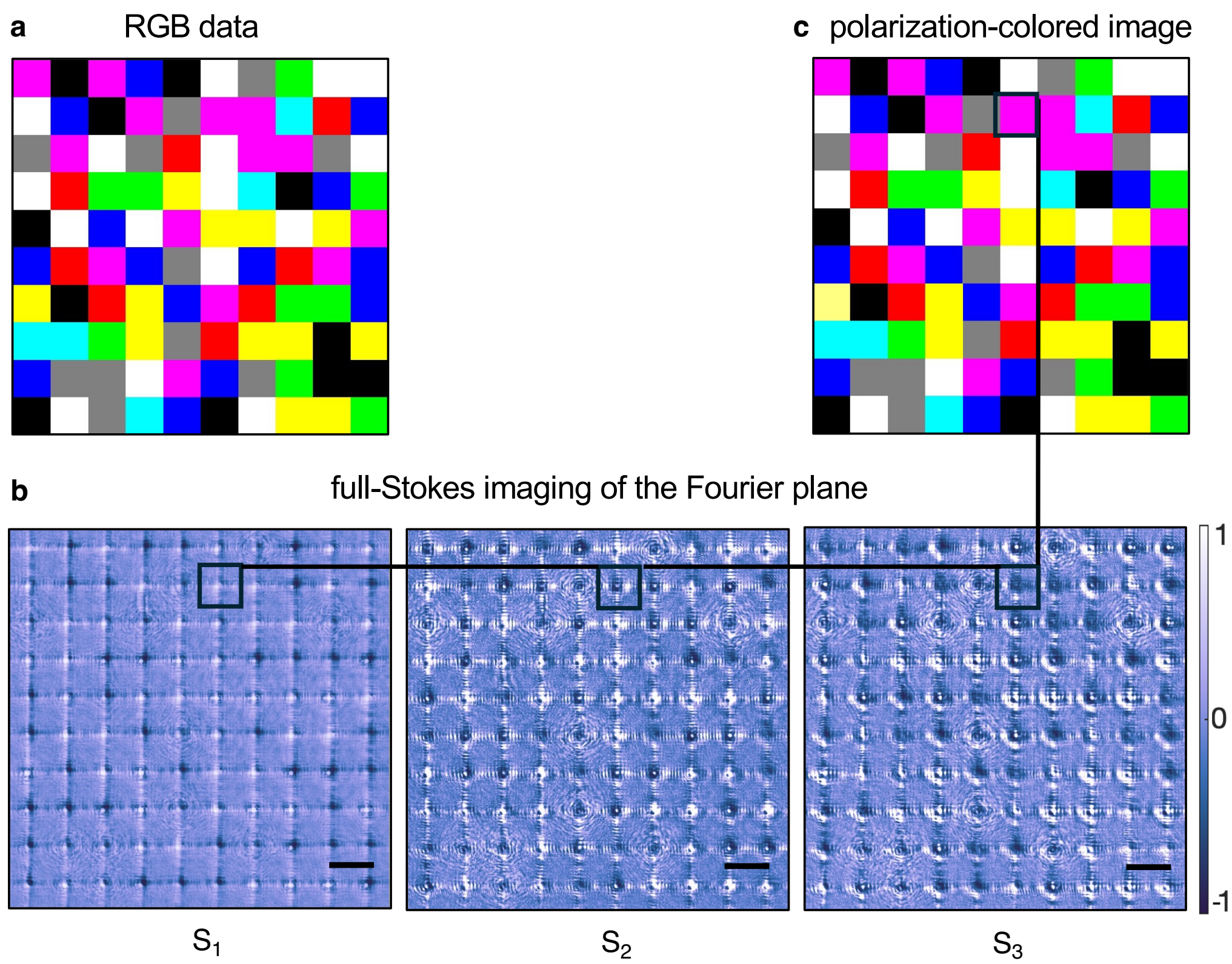

Supplementary Figure 12. Polarization-colored image by the focal spots. **a** Target RGB data (10×10 resolution) that is encoded in SOP and DOP according to the color mapping in Fig. 4a. **b** Measured Stokes maps of the Fourier plane. The Stokes parameters obtained by averaging over a region around each spot (boxed area) give the RGB image in **c**. Scale bars are 0.3 mm.

SUPPLEMENTARY NOTE 7. PHOTONIC COMPUTING WITH SOP AND DOP

In this section, we report the model of the high-dimensional photonic neural networks (PNN), along with additional experimental and numerical results on the photonic classification of RGB images in which the color information is encoded in a single high-dimensional channel by spatial modulation of the SOP and DOP.

A. Model and simulation of the high-dimensional PNN

We model the operation of the optical random layers on beams spatially modulated in SOP and DOP within the framework of the vector transmission matrix (VTM) [3]. The VTM describes the linear transformation $\mathbf{E}_{\text{out}} = \mathbf{T}\mathbf{E}_{\text{in}}$ performed by a scattering medium on the polarized vector field in terms of K input and G output modes. The size of $\mathbf{T}$ is $2G \times 2K$. We express the VTM in the horizontal (H) and vertical (V) polarization basis, corresponding to the x and y directions. Being the input field $\mathbf{E}_{\text{in}} = (\mathbf{E}_1, .., \mathbf{E}_k, .., \mathbf{E}_K)$ with $\mathbf{E}_k = (E_k^x, E_k^y) = (|E_k^x| \exp i\delta_k^x, |E_k^y| \exp i\delta_k^y)$ and phase $\delta_k = \delta_k^y - \delta_k^x$ in the kth mode, the output field $\mathbf{E}_{\text{out}} = (\mathbf{E}_1, .., \mathbf{E}_g, .., \mathbf{E}_G)$ in the gth mode $\mathbf{E}_g = (E_g^x, E_g^y)$ writes as

$$\begin{pmatrix} E_g^x \\ E_g^y \end{pmatrix} = \sum_k \begin{pmatrix} t_{gk}^{xx} & t_{gk}^{xy} \\ t_{gk}^{yx} & t_{gk}^{yy} \end{pmatrix} \begin{pmatrix} E_k^x \\ E_k^y \end{pmatrix}, \tag{18}$$

where the complex coefficient t_{gk}^{rs} connects the r-component of the kth input field (E_k^r) to the s-component of the gth output field (E_g^s). For a thick scattering medium and $G \gg 1$, the VTM follows random matrix theory and the elements t_{gk}^{rs} are well approximated by independent identically distributed Gaussian complex numbers [4].

We model the stack of scattering media as cascaded VTMs. Each scatterer thus performs an input-output linear transformation of the form of Eq. (18) on the polarized component of the field. We define as $\mathbf{T}_h$ the VTM of the hth scattering layer and model the field transmitted by the hth scatterer and incoming to the next one as composed by G_h spatial modes. The field through n cascaded layers thus reads as

$$\mathbf{E}_{\text{out}} = \mathbf{T}_n \dots \mathbf{T}_h \dots \mathbf{T}_1 \mathbf{E}_{\text{in}}, \tag{19}$$

where $\mathbf{T}_h$ has $2G_h \times 2G_{h-1}$ independent random complex coefficients $t_{gk}^{rs}(h)$, being $G_n = G$ and $G_0 = K$ the number of output and input modes.

The optical layers operate by mapping an input modulated field into a detected intensity vector. The field transfer function in Eq. (19) applies to a coherent field, i.e., to a polarization-structured beam made of fully polarized states. In this case, the input field is defined by the set of Stokes parameters $S_1^k = |E_{\rm in}^x|^2 - |E_{\rm in}^y|^2$, $S_2^k = 2|E_{\rm in}^x||E_{\rm in}^y|\cos\delta$, $S_3^k = 2|E_{\rm in}^x||E_{\rm in}^y|\sin\delta$, with $E_{\rm in}^x = E_k^x$, $E_{\rm in}^y = E_k^y$, and $\delta = \delta_k$ that indicate the kth macromode. The resulting intensity $\mathbf{I}_{\rm out} = (I_1, .., I_g, .., I_G)$ in the gth output mode is given by Eq. (19) as $I_g = |E_g^x|^2 + |E_g^y|^2$. The intensity I_g corresponds to the signal experimentally detected by the camera in the gth binned pixel.

To simulate the operation of cascaded VTMs on a beam spatially modulated in DOP and SOP, we express the input state of the kth mode of DOP ρ as the incoherent superposition

$$|E_\rho\rangle\langle E_\rho| = \frac{1+\rho}{2}|E_{\rm in}\rangle\langle E_{\rm in}| + \frac{1-\rho}{2}|E_{\rm in}^\perp\rangle\langle E_{\rm in}^\perp|, \tag{20}$$

where $|E_{\rm in}\rangle$ indicates the SOP of $\mathbf{E}_{\rm in} = (E_{\rm in}^x, E_{\rm in}^y)$ and $|E_{\rm in}^\perp\rangle$ is its orthogonal, $\mathbf{E}_{\rm in} \cdot \mathbf{E}_{\rm in}^\perp = 0$. In Eq. (20) the macromode index k has been omitted for clarity. We calculate the output intensity vector as $\mathbf{I}_\rho = \mathbf{I}_{\rm out} + \mathbf{I}_{\rm out}^\perp$ by applying Eq. (19) separately on $\mathbf{E}_{\rm in}$ and $\mathbf{E}_{\rm in}^\perp$. This approach to simulate the scattering of DOP-structured light is general and does not depend on the micromode structure that we employ to achieve the DOP modulation.

The transformation performed by the optical layers is specified by the product of the matrices $\mathbf{T}_h$ in Eq. (19) and depends on the properties of the submatrices $t^{rr}(h)$ and $t^{rs}(h)$ that model the hth scatterer. To simulate the ground-glass diffusers employed in our PNN, we generate Gaussian submatrices $t^{rr}(h)$ and $t^{rs}(h)$ that satisfy the property $|t^{rs}(h)| \ll |t^{rr}(h)|$, i.e., non-diagonal submatrices have random complex coefficients of amplitude much smaller than diagonal submatrices. This relation takes into account that multiple scattering in thin media causes small polarization variations [5]. We apply this condition by imposing $\langle|t^{rs}(h)|\rangle = \tau\langle|t^{rr}(h)|\rangle$, where $\langle\rangle$ is the average over the $G_h \times G_{h-1}$ submatrix entries. The parameter τ reflects the coupling between the field components induced by the medium. We find that the accuracy of the PNN depends on the value of τ. We fix $\tau = 0.02$, a typical value verified by the measured VTMs of our diffusers. The sizes G_h are tunable parameters of the cascaded VTM model but, for reducing computational efforts, we set $G_h = K$ for every intermediate layer as a coarse-grained approximation of the field between the diffusers.

The model is applied on the classification of RGB images from the CIFAR-10 dataset (https://www.kaggle.com/c/cifar-10/). We encode the RGB sample in the Stokes param-

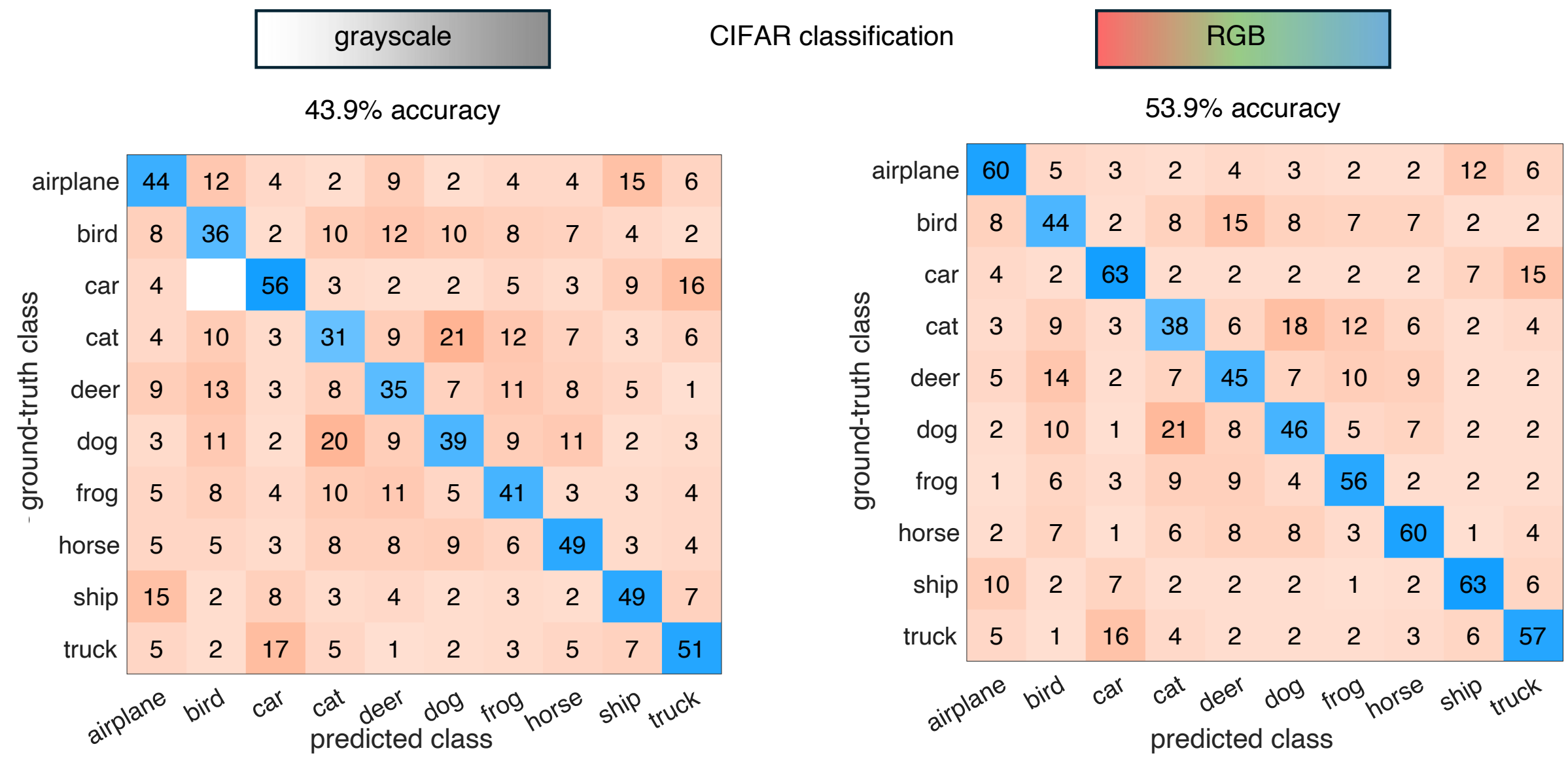

Supplementary Figure 13. Numerical results on the photonic classification of the CIFAR-10 dataset. Test confusion matrices for grayscale images encoded in phase and RGB images encoded in DOP and SOP. Results refers to a PNN with $n = 4$ random optical layers (diffusers).

eters according to the polarization-color map (see Methods) and we obtain the input field $\mathbf{E}_{\text{in}}$ on $K = N = 32 \times 32$ input modes. The transmitted intensity $\mathbf{I}_\rho$ is calculated on $G = 200 \times 200$ output modes. Then, we use the same digital backend (Methods) and training employed on the experimental intensity. Numerical simulations that agree with the experimental results in Fig. 5b are provided by the confusion matrices of the blind test reported in Supplementary Fig. 13. The simulated accuracy confirms the performance enhancement enabled by the polarization encoding and processing of the RGB data. The enhancement is even more significant than in experiments, amounting to 10%. Supplementary Fig. 14 reports the accuracy by decreasing the number of hidden nodes of the digital backend. A larger performance of the RGB encoding is maintained even for a PNN with less than ten digital nodes, a condition where the grayscale encoding performs poorly. This indicates that polarization encoding can be especially useful in the few-digital-node regime.

To understand the enhancement provided by the RGB encoding, we consider that phase-only (grayscale) encoding corresponds to a phase-modulated beam polarized along x. In this case, although the number of output (input) modes G (K) is the same as in the RGB case,

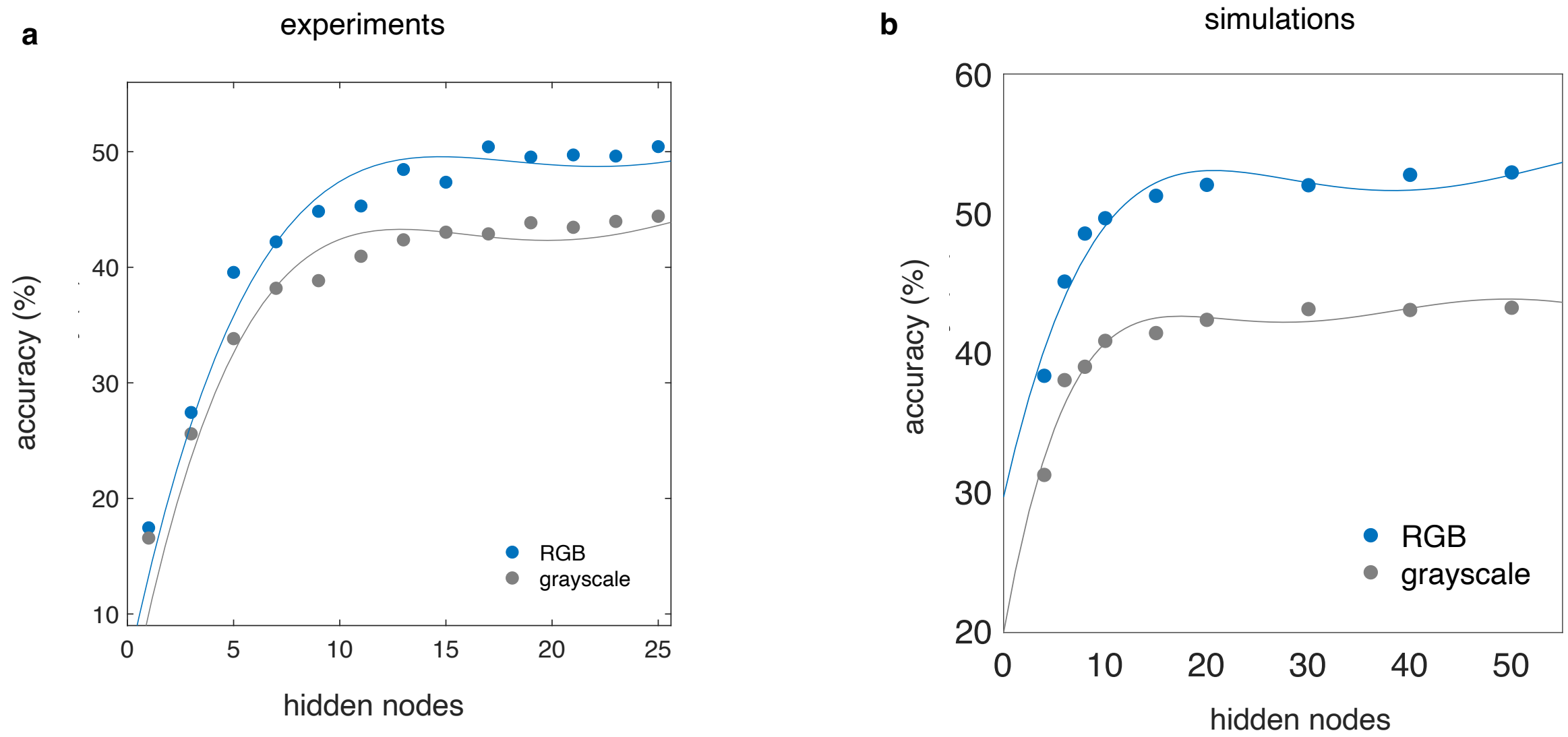

Supplementary Figure 14. Few-digital-node operation of the PNN on CIFAR-10 classification. Accuracy varying the number of hidden digital nodes for grayscale (phase-only) and RGB (DOP and SOP) encoding. **a** Experimental and **b** numerical results for $n = 4$ optical layers.

only the submatrices $t^{xx}(h)$ (scalar transmission matrices) intervene in the optical projection. By polarization-encoding, we pass from a layer expressed by $K \times G$ coefficients to a layer made by $2K \times 2G$ coefficients. In total, the optical part of the simulated high-dimensional PNN has $(n-1) \times (4K^2) + (2K \times 2G)$ complex random weights, while phase encoding gives a transformation specified only by $K \times G$ elements (product of random complex coefficients). The expressiveness is further augmented by the fact that every VTM acts on both the orthogonally-polarized fields composing the DOP-structured input according to Eq. (19). This larger expressiveness explains the superior performance of the high-dimensional PNN.

Simulations also reproduce the accuracy improvement observed as more diffusers are stacked in the high-dimensional PNN, as reported in Fig. 5c. An enhancement of nearly 4% is found by passing from $n = 1$ to $n = 4$ optical layers. For $n = 4$, the accuracy saturates to the value found for a thick scattering medium, i.e., for a VTM having diagonal and non-diagonal submatrices of equal amplitudes ($\tau = 1$). Therefore, the behavior by increasing n is strictly related to the relative amplitude of the submatrices $t^{rs}(h)$ that characterizes the scattering layers. These determine the expressiveness of the mapping between the input

polarization to the output intensity. Note that we do not observe an improvement with the number of diffusers n for grayscale encoding and scalar transmission. This is consistent with the linearity of the transformation since cascading two scalar transmission matrices results into an equivalent random matrix (although with different statistical properties).

B. High-dimensional photonic extreme learning machine

To show the applicability of the spatial DOP modulator to different optical computing architectures, we implement a variation of the random PNN that further reduces the digital computation cost. We integrate polarization-encoding into a photonic extreme learning machine (PELM), generalizing also this paradigm to a higher dimension. PELMs operate by a hidden optical system (reservoir) that project the input vector into a larger feature space where a linear digital classifier performs the classification. This results into a very simple training, which can be performed online and efficiently. The operation principle is detailed in Ref. [6]. In our high-dimensional implementation, the optical reservoir is still composed by the stack of diffusers. The optical transformation of the input field is performed in close analogy to the random PNN, but the digital backend is now made by a set of digital weights that multiplies the output intensity vector. The transmitted intensity is sampled on C spatial modes (camera pixels) taken randomly across the speckle pattern, which form the so-called hidden vector. The dimension C of the hidden vector is indicated as the number of features. A matrix of weights multiplies the hidden vector to give the output vector providing the classification outcome. These linear weights are the only trainable parameters. Details on the training by ridge regression can be found in Ref. [6].

Supplementary Fig. 15 reports test results on the CIFAR-10 classification, comparing the high-dimensional PELM that operates on polarization-colored images with the phase-only PELM on grayscale images, both in experiments and simulations. The accuracy is lower than the accuracy obtained with the random PNN, as expected considering the simpler training, but still remarkable. The large enhancement (6% on the experimental device) enabled by the RGB encoding is confirmed also in the PELM scheme. The enhancement remains almost constant as the number of intensity features C is reduced. These additional results suggest an advantage in computing provided by the high-dimensional encoding that can generalize to other free-space architectures such as SLM-based processors and diffractive NNs.

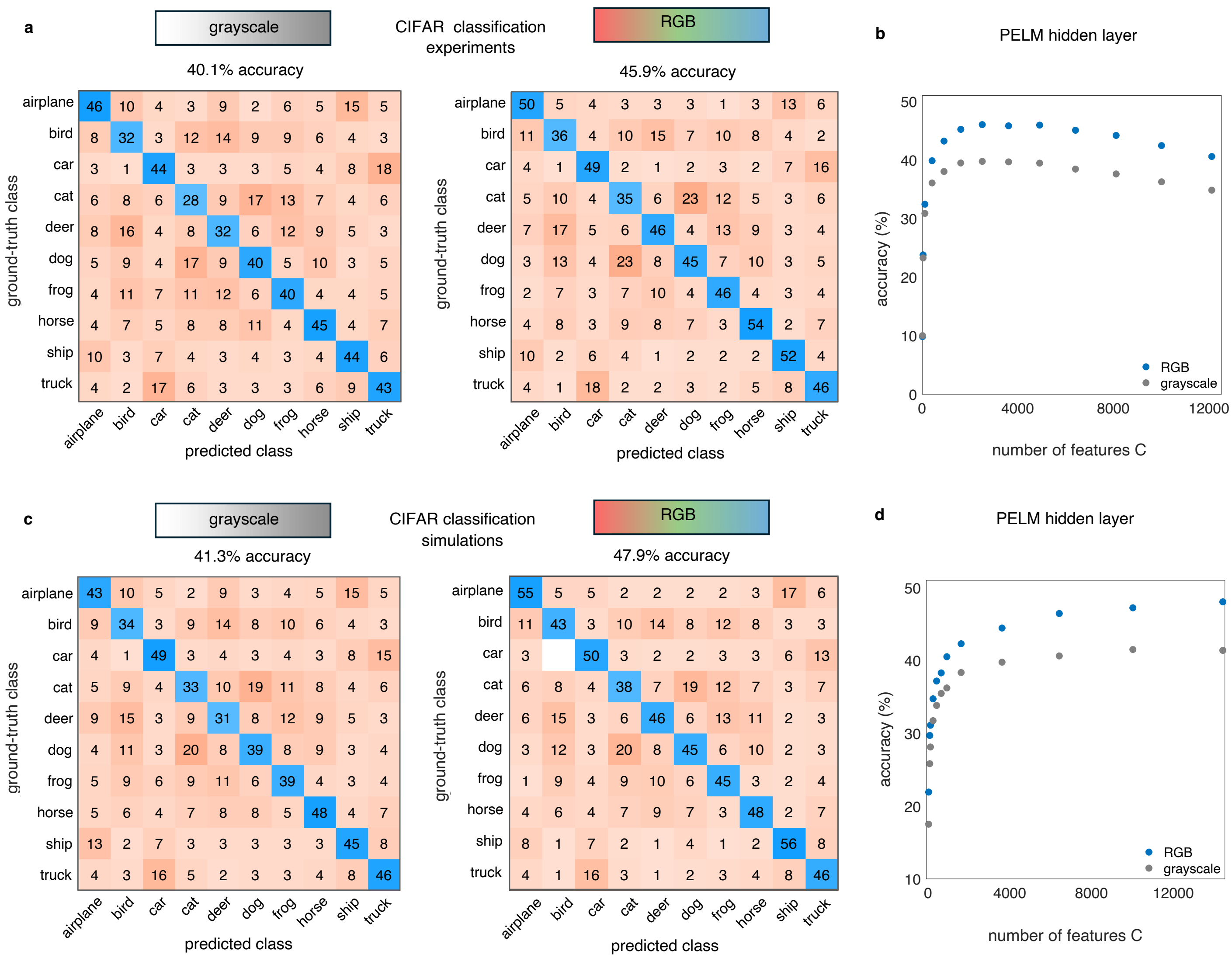

Supplementary Figure 15. High-dimensional photonic extreme learning machine (PELM). **a** Experimental and **c** numerical confusion matrices for the classification of the CIFAR-10 dataset using grayscale and RGB encoding. **b**, **d** Corresponding test accuracy varying the number of hidden features.

SUPPLEMENTARY NOTE 8. ROBUSTNESS AND SECURITY OF THE MULTIDIMENSIONAL ENCRYPTION

Speckle-based encryption benefits from the complexity of the physical encryption key and it has demonstrated high security for grayscale images. We have generalized the paradigm to RGB images by polarization encoding. In this section, we analyze the robustness of our multidimensional speckle-based encryption scheme on RGB images and complement the security analysis in Fig. 6 by including an additional level of authorization. We use the Pearson correlation coefficient (PCC) between the plaintext and decrypted image as an interpretable performance metric. The PCC is computed as the mean over the PCC of the three vectors of the RGB images. Similar results are obtained by using other figures of merit to quantify the similarity between images, such as the structural similarity (SSIM) index and the peak signal to noise ratio (PSNR).

We assess the robustness of the ciphertext with respect to various experimental factors. For this task, we consider 16×16 4-bit RGB images from the pixel-art dataset (www.kaggle.com/datasets/ebrahimelgazar/pixel-art). The decryption DNN is trained on 20000 of such images. In order to simulate a noisy environment, we add Gaussian noise at the single pixel level to the ciphertexts used for testing. The noise amplitude is varied with respect to the standard deviation σ of the ciphertext. The results in Supplementary Fig. 16 show that noise has a minor effect on the decryption accuracy until it reaches an amplitude around $\sigma = 0.001$. This high threshold indicates that the decryption scheme is robust with respect to the presence of noise at the receiver. Above this level, noise significantly alters the ciphertext and the PCC rapidly goes to zero. We also analyze the effect of the ciphertext size (i.e., the number of pixels of the received speckle pattern) on the decryption performance. A ciphertext of smaller size leads to a reduced decryption accuracy. However, we observe that the decryption remains effective also for a ciphertext of only 50×50 pixels.

The decryption DNN operates by reconstructing the input polarization information hidden within the intensity speckle pattern. Therefore, obtaining the correct plaintext requires also knowing the exact polarization encoding employed by the sender, i.e., the mapping between the RGB channels and the Poincaré sphere $[R, G, B] \leftrightarrow [S_1, S_2, S_3]$. This provides an additional security key that can be chosen arbitrarily in the encryption protocol. As demonstrated in Supplementary Fig. 17, only when using the correct security key the plain-

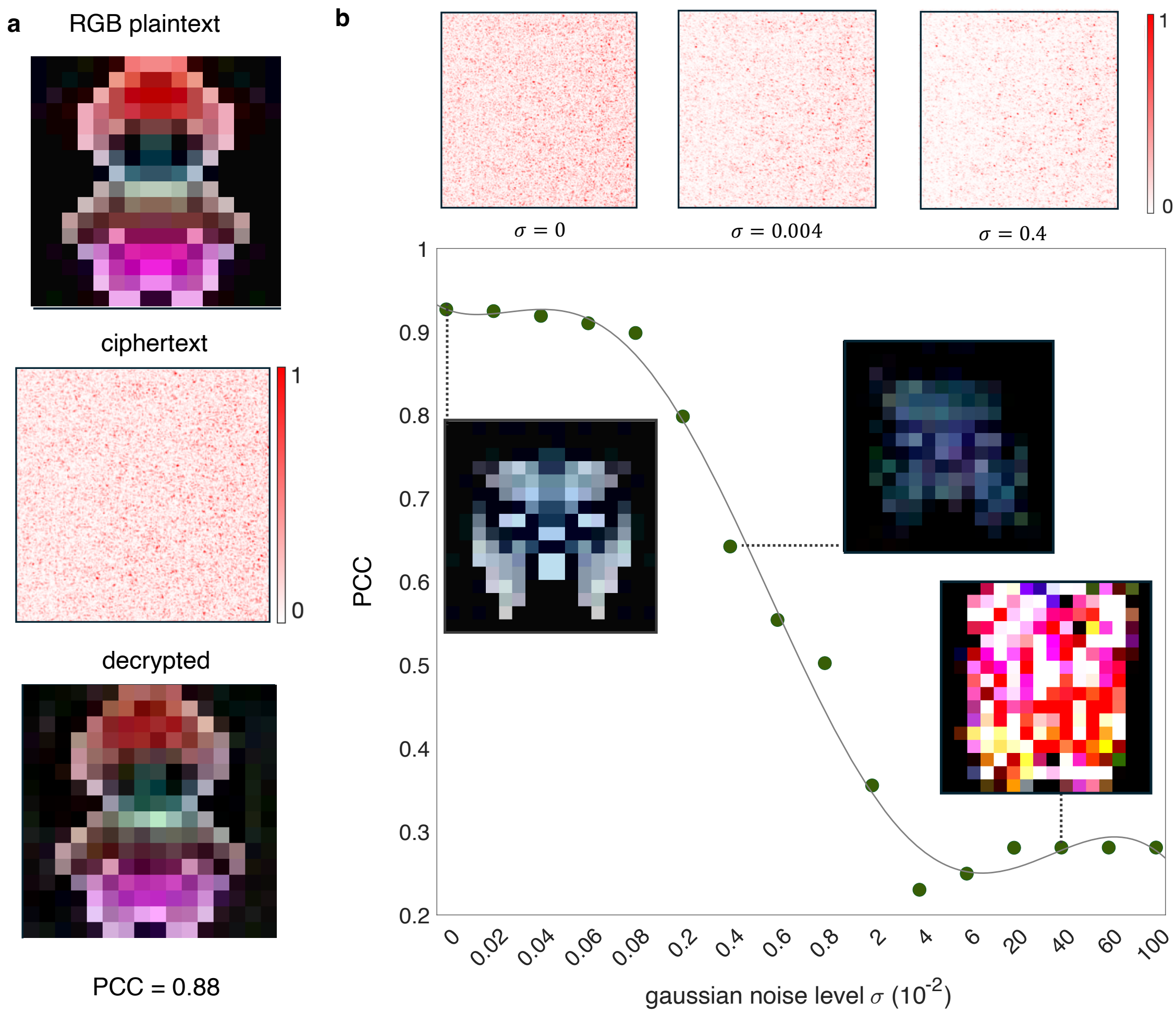

Supplementary Figure 16. **a** Encryption-decryption of a RGB image from the pixel-art dataset. **b** Impact of detection noise on the decryption quality. PCC versus the amplitude of Gaussian noise affecting the ciphertext (inset).

text is correctly deciphered. Any different security key leads to a decryption with wrong colors. Therefore, multidimensional encryption provides an additional security level through the polarization encoding, which further protects the cryptosystem against an attacker who has gained access to the decryption network.

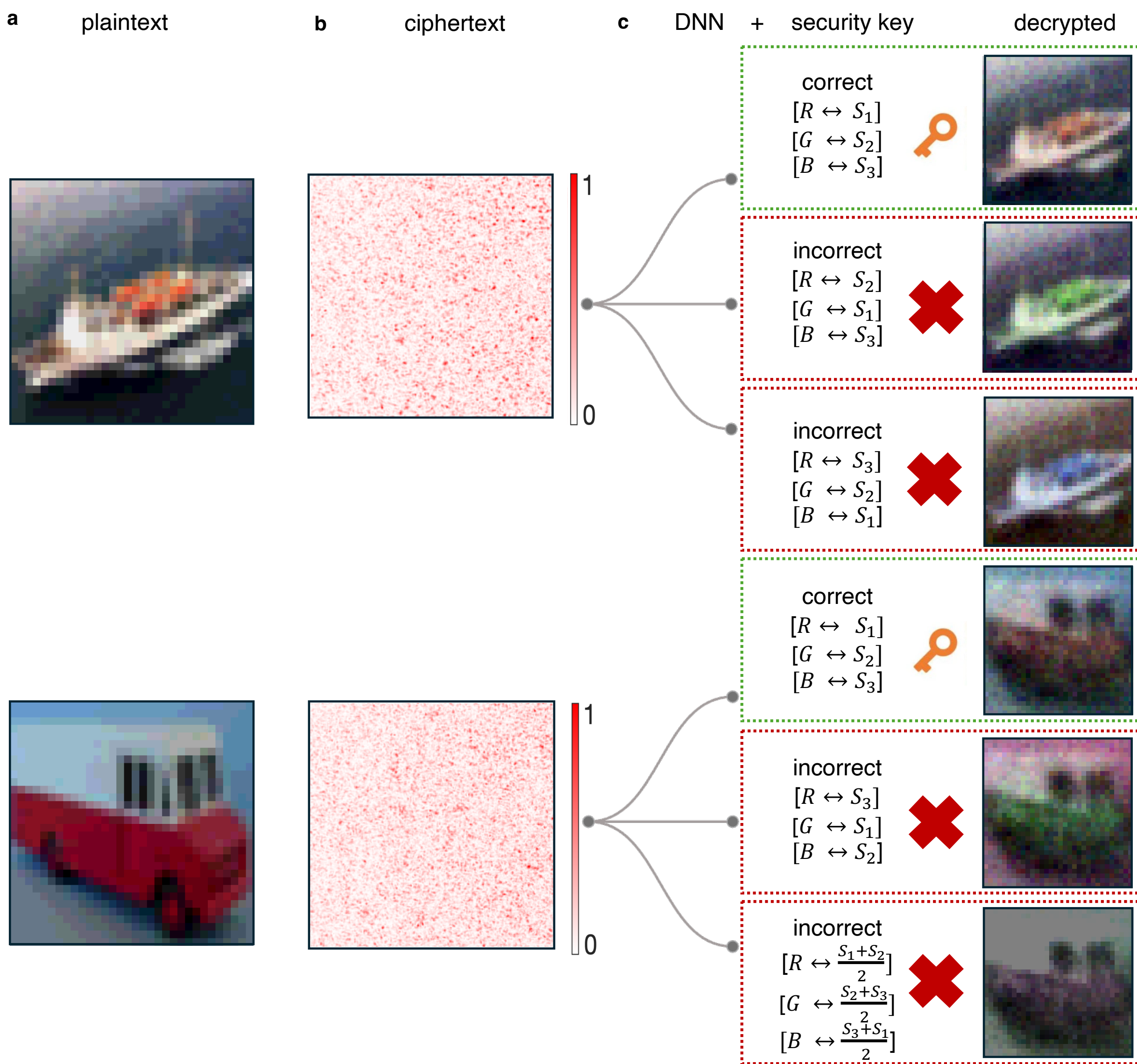

Supplementary Figure 17. Polarization encoding as an intrinsic security key. **a** RGB plaintexts and **b** corresponding ciphertexts. **c** Decryption with the same trained DNN but different mappings between the recovered polarization states and RGB channels. The correct colors of the plaintext are deciphered only via the correct security key used in the encryption, while any other mapping prevents correct decryption.

SUPPLEMENTARY NOTE 9. MACROMODE CROSSTALK ANALYSIS

Macromode crosstalk is avoided by employing d buffer pixels between adjacent macromodes. Compared to the macromode size, the buffer footprint is not negligible at maximum resolution. Supplementary Fig. 18a shows the effective fill factor for different macromode sizes. For $L = 25$ pixels and $d = 6$ buffer pixels per side, the fill factor is approximately 0.65. This value can be increased to 0.75 by reducing the buffer width to $d = 4$ pixels, without introducing significant crosstalk.

The effect of macromode crosstalk is experimentally quantified by programming two neighboring macromodes at a distance d within a uniformly polarized background. We vary the SOP and DOP of one macromode (the control macromode) and measure the deviation induced in the neighboring polarization state (the reference macromode) from its target value. The method is illustrated in Supplementary Fig. 18b,d through polarization images of the SLM plane showing the two neighboring macromodes in two different configurations. We first quantify the crosstalk effect on the SOP. In this case, the control and reference macromodes are fully polarized. They are programmed with different SOPs (e.g., H and V, as shown in Supplementary Fig. 18b). The deviation between the programmed and measured SOP of the reference is quantified by the relative error in the Stokes parameters. Supplementary Fig. 18c reports ΔS_1, averaged over several control and reference SOPs, while the buffer length d is varied. The error decreases with d and becomes negligible for $d = 6$ pixels. The measured value of approximately 0.015 is comparable to that obtained for an isolated macromode. The error remains below 0.02 even for d=4 pixels, indicating that the buffer width can be reduced to this value without compromising modulation accuracy.

To quantify crosstalk impact on the DOP modulation, we measure the DOP of the reference macromode while varying the DOP of the control one. In this case (Supplementary Fig. 18d), the reference is programmed with $\rho = 1$ so that the measurement allows us to isolate the contribution associated with the DOP leakage from the neighboring macromode. Supplementary Fig. 18e shows the relative error $\Delta\rho$ for $d = 5$ pixels. The deviation slightly increases as the control macromode depolarizes, but remains below 0.08. Approximately half of this value arises from the uncertainty of the setup in modulating the DOP of the single macromode. Therefore, residual crosstalk contributes at most an additional error of 0.04 to the DOP and remains well below this value throughout most of the DOP range.

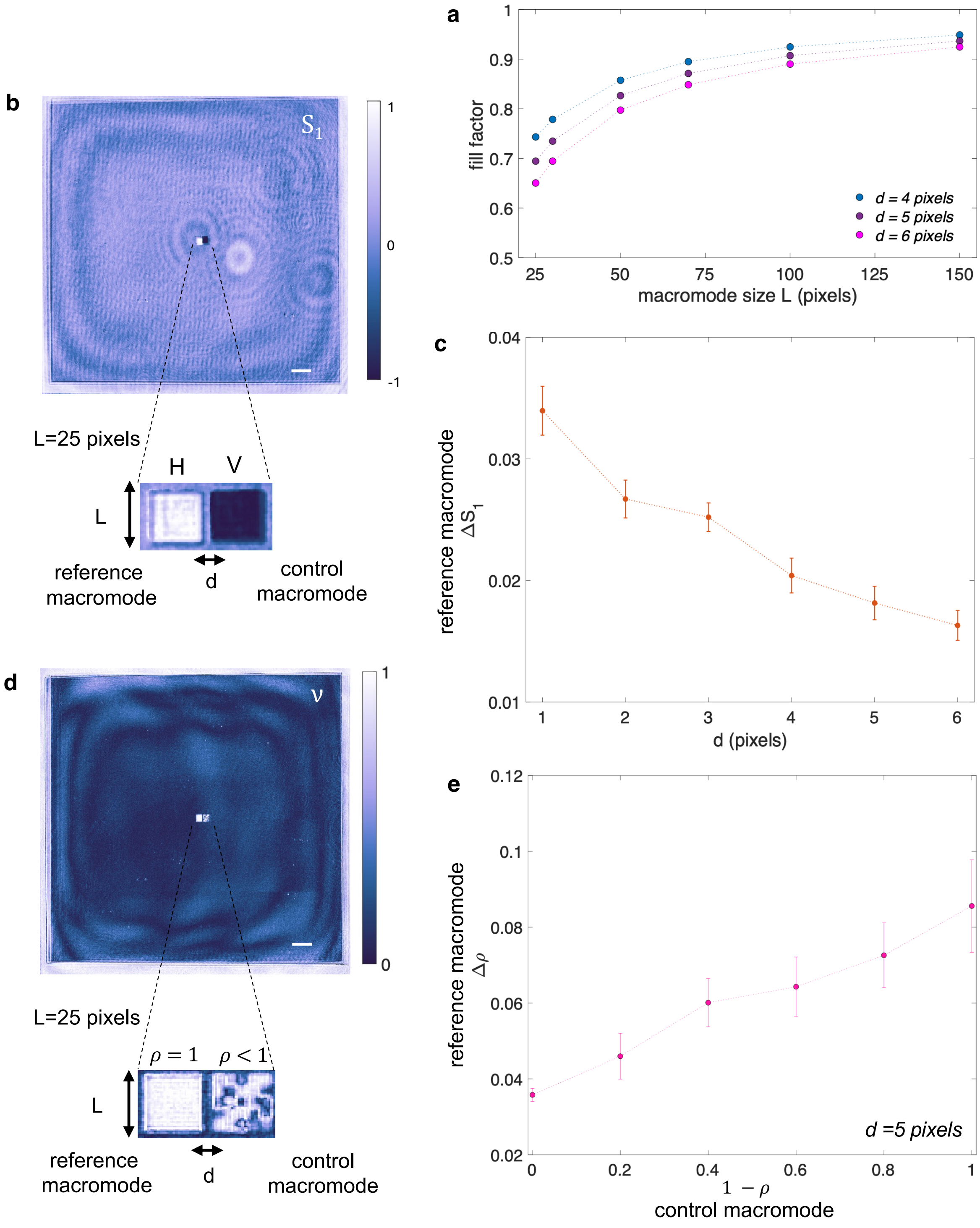

Supplementary Figure 18. Experimental analysis of the inaccuracy due to macromode crosstalk. **a** Effective fill factor for different macromode sizes. **b** Method for measuring the induced error on the SOP. The reference and control macromode on the SLM are shown via the S_1 image. **c** Error on S_1 as a function of the buffer length d. **d** Method for quantifying the DOP leakage. The two macromode with different DOP are shown by imaging the linear degree ν. **e** Error $\Delta\rho$ on the reference macromode as a fuction of the control DOP.

SUPPLEMENTARY NOTE 10. COMPARISON WITH SPATIAL ENCODERS

Ref.	dataset	RGB image resolution	optical encoding	number of SLM pixels per RGB pixel	classification accuracy (%)
[7]	CIFAR-10	32x32x3	phase	100	49.1 - 52.2
[8]	STL-10	96x96x3	phase	75	--
[9]	CIFAR-10	32x32x3	amplitude	108	45.0
this work	CIFAR-10	32x32x3	DOP and SOP	625	50.4

Supplementary Table 1. Comparison of spatial optical encoders for RGB images in PNNs. Highlighted is the number of SLM pixels required to encode a single RGB pixel of the input image.

SUPPLEMENTARY REFERENCES

[1] Schaefer, B., Collett, E., Smyth, R., Barrett, D., & Fraher, B. (2007). Measuring the Stokes polarization parameters. American Journal of Physics, 75(2), 163-168.

[2] E. Wolf, Introduction to the Theory of Coherence and Polarization of Light (Cambridge University Press, Cambridge, 2007).

[3] Tripathi, S., Paxman, R., Bifano, T., & Toussaint Jr, K. C. (2012). Vector transmission matrix for the polarization behavior of light propagation in highly scattering media. Optics express, 20(14), 16067-16076.

[4] Yu, H., Hillman, T. R., Choi, W., Lee, J. O., Feld, M. S., Dasari, R. R., & Park, Y. (2013). Measuring large optical transmission matrices of disordered media. Physical review letters, 111(15), 153902

[5] de Aguiar, H. B., Gigan, S., & Brasselet, S. (2017). Polarization recovery through scattering media. Science Advances, 3(9), e1600743.

[6] Pierangeli, D., Marcucci, G., & Conti, C. (2021). Photonic extreme learning machine by free-space optical propagation. Photonics Research, 9(8), 1446-1454.

[7] Wang, H., Hu, J., Morandi, A., Nardi, A., Xia, F., Li, X., ... & Gigan, S. (2024). Large-scale photonic computing with nonlinear disordered media. Nature Computational Science, 4(6), 429-439.

[8] Kilinc, F. N., & Tegin, U. (2026). Self-optimizing multichannel optical computing. arXiv preprint arXiv:2601.07574.

[9] Hu, Z., Li, S., Schwartz, R. L., Solyanik-Gorgone, M., Miscuglio, M., Gupta, P., & Sorger, V. J. (2022). High-throughput multichannel parallelized diffraction convolutional neural network accelerator. Laser & Photonics Reviews, 16(12), 2200213.
