# Supplementary material for: Degree-of-polarization modulation for high-dimensional optical computing
Source: Nature. 2026 Aug 12;657(8131):377–84. doi: 10.1038/s41586-026-10891-z (PMC13558076; doi:10.1038/s41586-026-10891-z)
Supplement: Supplementary file 2 [file 41586_2026_10891_MOESM2_ESM.pdf]

nature portfolio

Peer Review File

Degree-of-polarization modulation for high-dimensional optical computing

Corresponding Author: Professor Davide Pierangeli

Any redactions in this file are there to maintain patient confidentiality, the confidentiality of unpublished data, or to remove third-party material.

This file contains all reviewer reports in order by version, followed by all author rebuttals in order by version.

Version 0:

Reviewer comments:

Referee #1

(Remarks to the Author)
The authors demonstrate the first programmable spatial modulation of the degree of polarization (DOP) using a phase-only spatial light modulator (SLM) combined with waveplates. While conventional optical systems can independently control phase, amplitude, and the state of polarization (SOP), spatially varying DOP has generally remained inaccessible, limiting applications in high-dimensional photonics. By tailoring the statistical distribution of polarization states across microscopic regions of a beam, this approach enables independent control of both SOP and DOP over macroscopic spatial modes, potentially accessing the full three-dimensional Poincaré sphere. This capability facilitates structured light with enhanced information encoding, as illustrated in two key applications: fully parallel coherent classification of color images mapped from RGB space into a single-wavelength laser beam using a high-dimensional photonic neural network, and secure multidimensional optical encryption leveraging polarization scattering.

This work represents a first-of-its-kind experimental demonstration of programmable spatial DOP modulation, making it both conceptually innovative and practically significant. Prior to this study, optical modulation techniques could manipulate phase, amplitude, and SOP spatially, but no method existed for arbitrary spatial DOP control. Earlier approaches were limited to global or homogeneous DOP or relied on static metasurfaces without per-pixel programmability. The authors address this gap using a statistical approach, engineering the probability distribution of polarization states at a microscopic scale to achieve controlled partial polarization, rather than only deterministic SOP transformations. Notably, this method accesses the full Poincaré sphere, including partially polarized states, opening a new dimension for structured light. The resulting high-dimensional encoding can substantially increase the information capacity of optical fields, demonstrated through both a fully parallel photonic neural network for image classification and multidimensional optical encryption with enhanced security.

Overall, the manuscript is well written and the results clear. One point that might warrant further discussion is the potential limitation of using only a single tunable phase per SLM pixel in combination with waveplate angles. For a single macromode, the method appears capable of substantial control over both the DOP and SOP, by controlling the phase and phase diversity over the micromodes, and the angles of the waveplates. However, when multiple macromodes are considered, I assume that all of them rely on the same waveplate angles. In this configuration, the SLM introduces a controllable phase delay between the linear polarization components, while the quarter-wave and half-wave plates provide only a static rotation of the Poincaré sphere. Consequently, for a given coherent input polarization state, the achievable output states as the SLM phase varies may trace only a one-dimensional curve on the Poincaré sphere, rather than spanning the full two-dimensional space of all possible pure polarization states. If this is the case, arbitrary polarization control per pixel could be limited, potentially restricting the spatial diversity of polarization patterns across multiple macromodes. Spatial averaging over multiple phase settings can reduce the measured degree of polarization and simulate partial polarization, but it may not fully overcome the single-degree-of-freedom constraint inherent to the current SLM and waveplate setup.

In their experiments, the authors show that polarization-based RGB encoding can improve information throughput in an optical neural network. Using a subset of four CIFAR categories, the network achieves 79% classification accuracy when

processing polarization-encoded images through optical-only layers with a minimal digital readout. Remarkably, even with an extremely small digital backend of just two hidden nodes, the network reaches 72% accuracy, whereas phase-only grayscale encoding of the same images performs poorly (~51%). These results suggest that encoding RGB information via DOP and SOP allows more information to be carried in a single optical channel, enhancing classification performance in low-digital-complexity optical networks. It should be noted, however, that these benchmarks were obtained on a limited subset of CIFAR within a specialized experimental setup and are not directly comparable to full-scale digital deep neural networks.

The authors further extend speckle-based optical encryption to multidimensional data by encoding RGB images into the DOP and SOP of a single-wavelength beam. The polarization-encoded beam is encrypted through a scattering medium acting as a large, unclonable physical key, with decryption achieved via a small deep neural network that reconstructs the full polarization content from intensity-only measurements. This approach demonstrates high-fidelity reconstruction (SSIM > 0.9) and adds an additional layer of security through the secret RGB to Poincaré mapping. While the scheme significantly increases information dimensionality and physical-layer security compared to prior single-channel optical encryption methods, it remains a proof-of-principle system, sensitive to environmental stability and reliant on obscurity-based keys rather than mathematically guaranteed cryptography. Nevertheless, it represents a state-of-the-art demonstration of multidimensional optical encryption using polarization as an independent encoding degree of freedom.

Overall, the major advance of this work lies in the optical domain: the ability to programmably control the degree and state of polarization at a spatially resolved level opens a fundamentally new degree of freedom in structured light. This represents a conceptually and experimentally significant step forward, and I find it very exciting from the perspective of high-dimensional photonics and optical computing. At the same time, while the demonstrations are state-of-the-art, they remain proof-of-principle systems and may face practical challenges, including limited macromode diversity, SLM speed constraints, environmental sensitivity, and reliance on specialized experimental setups. Additionally, a more extensive comparison with existing literature could help clarify the relative advantages and limitations of this approach. Nonetheless, these results highlight a promising avenue for optical information processing that could inspire further developments in photonic neural networks, multidimensional encryption, and beyond.

Referee #2

(Remarks to the Author)
Summary of Research
In this manuscript, Petrini et al. propose an original idea to experimentally generate and leverage optical spatial modes with a programmable degree of polarization (DOP). While control of the state of polarization (SOP) is a mature technique, e.g., using wave retarders, the key innovation here is the introduction of DOP as a tunable degree of freedom. The authors achieve this by defining a “macromode” as an ensemble of “micromodes”, where the random phases are drawn from a Gaussian distribution with tunable mean and standard deviation parameters. Combined with polarization wave plates to control individual micromode SOPs, this setup generates a far-field macromode with tunable SOP and DOP. The authors further introduce the concept of “polarization color” and demonstrate its application in optical computing and image encryption.

The underlying physics is clear and interesting. The principle is well-explained, though the manuscript would benefit from a more rigorous analytical derivation of the tunable DOP. While most experimental results are solid, some claims feel overstated, and a few demonstrations are misleading. My detailed comments are provided below.

1. A significant concern is the potential for crosstalk between macromodes. Since the polarization-structured beam is generated in the far-field, each macromode is essentially a weighted summation of all the micromodes in the SLM plane. Doesn’t this mean that adjusting one macromode could inadvertently affect its neighbors, which complicates the independent control of DOP and SOP at a specific spatial location? This needs more explanation.

2. The core claim of enhanced high-dimensional encoding is, in my view, not well-justified. Let me elaborate. The method’s foundation is the grouping of multiple SLM pixels into a single macromode to define a (theta, chi, rho) tuple for “polarization color” encoding. This polarization color is used to encode a RGB pixel in natural images without using multi-wavelength sources. However, this grouping inherently sacrifices spatial parallelism. For instance, this work demonstrates encoding with N = 4, 25, and 64 macromodes, corresponding to 12, 75, and 192 encoded pieces of information using 3 channels. In contrast, a conventional method using the same SLM without such grouping can encode 4*3600, 25*3600, and 64*3600 pieces of information by treating each pixel as an independent channel (in a similar manner to spatial fan-out). Given that massive spatial parallelism already allows for encoding thousands of channels with a single-frequency laser, the claim that this approach is more “scalable” seems unfair and requires substantial re-evaluation.

3. What is the physical size of a formed macromode in the far field compared to the size of a micromode in the SLM plane? This information is important for understanding the spatial resolution of the system.

4. The application in optical computing leverages a complex medium, which connects to the field of physical reservoir computing. A key principle in reservoir computing is the use of a simple, linear digital backend for easy training. However, the authors employ a two-layer MLP as the digital backend. Though this may boost performance, it complicates the training process and undermines the core advantage of reservoir computing. In addition, the claim of “surpassing the performance of state-of-the-art deep optical neural networks [7]” feels disingenuous when a nontrivial digital network is doing much of the heavy lifting.

5. Figure 5a schematically illustrates optical encryption using a high-resolution van Gogh image, creating a strong impression. However, the actual experimental results in Fig. 5b are all low-resolution plain texts. This discrepancy is misleading and should be corrected. The schematic should accurately reflect the scale of the experimental validation. Furthermore, the authors should discuss a potential drawback of their method: compared to conventional speckle-based encryption using fully coherent light, the use of partially coherent light will smoothen the speckle contrast. This likely makes the ciphertexts for different plaintexts more similar, potentially degrading the decryption quality, which should be acknowledged.

6. Referring to the number of diffusers as “optical depth” is misleading, as this term is strongly associated with neural network layers. The diffusers are linear optical systems. The manuscript should use a more precise term. Besides, how to understand that the performance enhances with more diffusers since they are linear?

7. If information is encoded in the defined polarization color space (theta, chi, rho), is the mapping from this parameter space to the output vector field after the diffusers linear or nonlinear?

8. Supplementary Information, there is a typo in “divided into N square macromodes composed of N micromodes”.

Overall, while I find the core idea of generating tunable DOP original and compelling, the potential advantages of the proposed approach are, in my opinion, overexaggerated. This work represents a specialized advance in optics and photonics. And it does not seem to have the broad, interdisciplinary appeal required for Nature. It would be a better fit for a more specialized journal such as Nature Photonics, Nature Physics, Physical Review Letters, or Nature Communications.

Referee #3

(Remarks to the Author)
I co-reviewed this manuscript with one of the reviewers who provided the listed reports.

Referee #4

(Remarks to the Author)

The manuscript titled "Spatial degree-of-polarization modulation for high-dimensional optical computing and encryption," by A. Petrini, C. Conti, and D. Pierangeli introduces a method for programmable spatial modulation of the degree of polarization (DOP) using a phase-only spatial light modulator (SLM) and some polarization optics. By tailoring the statistical distribution of the phase across multiple micromodes, the authors achieve independent control of both DOP and state of polarization (SOP) across a smaller number of spatial regions of an optical beam, which they call macromodes. This enables the generation of structured light with arbitrary spatially varying polarization states that lie inside the Poincaré sphere. The authors demonstrate two applications: (i) encoding RGB images via “polarization colors” and processing them with a high-dimensional optical neural network (ONN), and (ii) secure optical encryption of multidimensional data using polarization-encoded speckle patterns.

The manuscript cites recent and relevant literature, including foundational works in optical computing, polarization optics, and optical encryption. The abstract and introduction are dense and somewhat repetitive. The abstract should more clearly distinguish the novelty from the applications. The introduction could be streamlined to focus on the technological gap and the contribution of this work. Figures are central to understanding the results but require clearer labeling and integration into the text. The conclusions are ambitious.

The core novelty lies in the programmable spatial control of DOP, which has not been previously demonstrated in a scalable and reconfigurable manner. The method is elegant and leverages existing SLM technology, making it broadly accessible. However, I believe that the claim that RGB encoding is not possible using SOP alone is overstated. There appears to be no reason why a direct mapping from the Stokes parameters (S1,S2,S3) to (R,G,B) is not feasible. In fact, polarization imaging is the same process put in a different direction, that is, mapping color to the SOP. The authors should either refute my reasoning, or revise this claim and clarify that DOP adds an additional degree of freedom, rather than being strictly necessary for RGB encoding. The integration of DOP modulation into ONNs and encryption systems is compelling, but the broader impact may be limited unless the authors can demonstrate scalability, robustness, and generalizability beyond the specific experimental setup.

The experimental methodology is sound and well-documented. The use of Gaussian phase distributions to control polarization statistics is well-justified, and the calibration of the modulator is thorough. The authors provide quantitative validation of their control over DOP and SOP, including polarimetry and full-Stokes imaging. The encryption and ONN experiments are supported by appropriate datasets and performance metrics. However, the manuscript would benefit from a more rigorous treatment of coherence. The authors describe the optical processing as “coherent,” yet DOP is directly linked to partial coherence. That is, the DOP sets a limit to the maximum attainable degree of coherence [J. Opt. Soc. Am. A 33, 2431-2442 (2016)], which contradicts the claim of coherent processing. This should be clarified, especially in the context of the ONN, where coherence may affect interference and propagation through scattering media.

The authors report classification accuracy, RMSE for polarization control, and SSIM for encryption fidelity. These metrics are

appropriate, but the statistical treatment could be strengthened. For example, confidence intervals or standard deviations across multiple trials would help assess robustness, although this is not a major concern. The encryption system's resistance to noise and adversarial attacks is briefly mentioned but not rigorously analyzed.

The conclusions are generally supported by the data, but some claims require attention. The assertion that DOP modulation enables RGB encoding seems to be an overstatement. The claim of coherent processing is not correct. The ONN performance is promising, and the improvement over grayscale encoding is well-demonstrated. However, the underlying reason for this improvement is not fully explored. Here, it seems that the inclusion of the DOP is a key factor, although there is no concrete data to support this idea. A deeper analysis of the improvement mechanism would strengthen the manuscript.

In it's present form the manuscript is not suitable for publication in Nature. However, it may be suitable after a revision where the following concerns are addressed:

1. Clarify the role of DOP vs SOP in RGB encoding and revise claims accordingly.
2. Reformulate the role of coherence.
3. Provide a deeper analysis of the ONN improvement.

Additionally, it would be desirable to explore scalability, e.g., higher-resolution images, faster modulation rates, or integration with metasurfaces.

Asst. Prof. Matias Koivurova
Center for Photonics Sciences
University of Eastern Finland

Version 1:

Reviewer comments:

Referee #1

(Remarks to the Author)
The authors have done substantial new work in response to my comments. The scaling demonstration to N = 1024 macromodes, the introduction of a two-SLM fully-programmable setup, the analytical derivation based on coherence theory, the VTM-based model of the PNN, and the move to full CIFAR-10 at native resolution all represent real progress. Several of my specific concerns are now addressed convincingly. However, some of my more fundamental objections, particularly regarding the scalability framing and the broader-impact claim, are responded to with clarification and reframing rather than with evidence that changes my view. Especially, on the question of suitability for Nature, my position is unchanged, though I recognize this is ultimately an editorial judgment rather than a technical one. The work is excellent and would in my view be very strong for Nature Photonics or Nature Communications.

Below I give a point-by-point response to the comments that I am not fully satisfied with.

Comment 2.1
I am mostly satisfied with the response of the authors but I do have one follow-up. The diffraction-length argument is sound and the buffer-pixel mitigation strategy is a reasonable engineering solution. The Fourier-plane microlens-array implementation is an elegant alternative that essentially eliminates the problem and I very much appreciated seeing it.
One follow-up, though: the buffer pixels are presented as a small overhead for larger macromodes, but at the minimum-size regime (L = 25 px, six blank pixels per side) the buffer footprint is not negligible. It would be interesting to add to the supplementary material the effective fill factor at different resolutions and quantify residual crosstalk experimentally e.g., by programming a single bright macromode against a uniformly dark background and reporting the measured DOP "leakage" into neighbours. In this way, readers can assess how negligible the crosstalk is in a quantitative way.

Comment 2.2
This was my central concern and remains a point where I still have the most reservation. I especially like that the 100× scaling to 1024 macromodes is a real demonstration, rather than just a promise. I feel the reframing by the authors is honest: the linear scaling with pixel count is the same as for conventional spatial encoders, and the advantage lies in the kind of information encoded per macromode rather than in raw channel count. The comparison with Ref. [45] is a fair quantitative benchmark, although the 625 vs 100 pixels argument is unclear in their response and would need some clarification. A direct table comparing pixels-per-encoded-RGB-pixel across the relevant prior work would be a great addition to the supplementary material to strengthen their point.

The wording in the revised text and abstract still uses "high-dimensional" in a way that can be interpreted as the distinction between (a) genuinely accessing a new physical degree of freedom (which is the case) and (b) increasing information density per encoded channel (which I understand now holds only modestly). I suggest the authors tighten the language or clarify exactly what is meant with "high-dimensional".

Comment 2.6
I am mostly satisfied with the response of the authors. Removing "optical depth" is appropriate. The VTM-based explanation, i.e. thin diffusers have small off-diagonal coupling, so cascading progressively populates the full 2N × 2N polarization-

mixing matrix, is physically reasonable and is now backed by Fig. 5c showing experimental and simulated agreement. Nevertheless I remain with a small concern: in the supplementary material the authors mention a saturation which should occur within a small number n=4. Does the experimental trend in Fig. 5c saturate within the n = 1–4 range shown, and is this consistent with the simulated VTM? Showing this saturation explicitly (or extending to n = 5–6) would be very convincing.

Comment 2.9
The authors have produced a genuinely improved manuscript. It shows that the demonstrator is more scalable. The results are now more rigorous theoretically and experimentally more complete. I now agree that the work goes meaningfully beyond proof-of-principle in several respects. However, my view on journal fit was primarily about about the breadth of the conceptual advance. Spatial DOP modulation is a real and elegant new capability, and the polarization-color framing is a genuinely nice idea. The applications shown (one-shot RGB classification on CIFAR-10 at 50.4%, multi-dimensional speckle encryption) are solid demonstrations within optics but do not establish the kind of cross-disciplinary impact that, in my judgment, distinguishes a Nature paper from an excellent specialized one. The authors' argument that "control of a new optical degree of freedom directly implicates a new degree of freedom for communication and computing" is reasonable in principle but is asserted rather than demonstrated. The CIFAR-10 accuracy is competitive with prior multi-channel PNNs rather than disruptive of any computing benchmark.

I therefore maintain that the natural home for this work is Nature Photonics, Nature Communications, or a similar venue, where the optics community will appreciate the genuine novelty.

Referee #2

(Remarks to the Author)
1.The authors have clearly explained that the encoding of the RGB image in the input field is nonlinear. I note that in the “Encoding colors in polarization” subsection of the Methods, the authors use the phrase “the linear map”. While this is technically correct when referring to the relationship between S1/S2/S3 and R/G/B, using the word “linear” in this context might cause confusion. I suggest simply deleting the word “linear” here or replacing it with another term.
2.Does the nonlinearity of the encoding scheme contribute to the enhanced performance of the high-dimensional PNN? I suggest adding a brief discussion to clarify this point.

Referee #3

(Remarks to the Author)
I co-reviewed this manuscript with one of the reviewers who provided the listed reports.

Referee #4

(Remarks to the Author)
I would like to commend the authors for the exceptionally thorough and serious revision of the manuscript. The revised version reflects a substantial amount of new experimental, theoretical, and numerical work, well beyond what is typically expected in a revision. In particular, the scaling of the spatial DOP modulator to 1024 independently programmable macromodes, the introduction of a fully programmable two-SLM implementation, and the addition of a rigorous coherence-theory and vector transmission matrix (VTM) framework significantly strengthen the manuscript. The revised manuscript is clearly more mature, more careful in its claims, and technically much more solid than the original submission.

The central conceptual contribution of the work remains the introduction of the degree of polarization as a genuinely new, programmable spatial degree of freedom for optical information encoding. The authors convincingly demonstrate that the DOP can be controlled independently of the SOP and that it can be spatially structured with high fidelity. From a physical standpoint, this is an important and underexplored resource. The extensive scaling analysis and the demonstration of high-resolution DOP and SOP patterns strongly support the claim that this is not merely a proof-of-principle effect, but a scalable and broadly accessible capability.

The treatment of coherence has markedly improved. The manuscript now correctly frames the optical processing as partially coherent, and the supplementary polarization-matrix analysis provides a solid theoretical foundation linking the programmed phase statistics to the resulting DOP. The clarification that the nonlinearity is not in the propagation but in the field representation and detection is an important point that is now handled carefully.

Regarding the polarization-based encoding of RGB information, the revised manuscript is now more clear and more internally consistent than before. The inclusion of the Newton wheel in the original submission was confusing, as that color space contains only fully saturated colors, which can be mapped directly onto the surface of the Poincaré sphere (which motivated my initial impression). The authors now explicitly and correctly frame the DOP as a requirement to achieve a one-to-one, volumetric mapping between the full RGB color space and polarization space, as opposed to mappings confined to the surface of the Poincaré sphere. While one may debate definitions of what constitutes a “genuine” color encoding, the revised presentation is mathematically coherent and much less ambiguous than in the original manuscript. The added experimental demonstrations of polarization-colored images at higher resolution further support this part of the work.

The optical encryption section has also been strengthened. The move to higher-resolution RGB plaintexts, the more careful discussion of reduced speckle contrast in partially polarized light, and the explicit analysis of robustness against cloning and misalignment attacks make this part of the manuscript more convincing and technically grounded. While the scheme remains a physical-layer encryption approach rather than a cryptographically rigorous one, it is now clearly presented as such, and its strengths and limitations are discussed more transparently.

I am more ambivalent about the optical neural network results. Compared to the original submission, the relative improvement in the classification accuracy has decreased: the original manuscript reported an improvement by roughly a factor of 1.30, whereas the revised manuscript shows a more modest improvement on the order of 1.16. At the same time, the experimental and conceptual complexity of the system has increased significantly. In their response, the authors imply that the reduced absolute classification accuracy is due to the more challenging and realistic task (the full CIFAR-10 dataset at native resolution). However, they do not mention the reduction in the relative performance improvement with respect to the original submission. It would be helpful if the authors could comment on this. As it stands, one may reasonably question whether the added complexity associated with DOP and SOP encoding is fully justified on the basis of the moderate classification accuracy gains.

That said, there is one aspect of the ONN results that I find genuinely interesting and somewhat undersold in the revised manuscript. The observation that the classification accuracy increases systematically with the number of cascaded diffusers is a conceptual advance in my opinion. The supplementary VTM analysis makes a compelling case that this effect arises from the vectorial nature of light and from polarization mixing accumulating across layers, combined with the nonlinear nature of the Stokes-parameter representation and intensity detection. In this sense, the field itself carries the nonlinearity, effectively relaxing the need for explicit nonlinear activation functions between optical layers. Although the absolute improvement is quite small, the fact that such a depth-dependent enhancement exists at all in a purely passive, linear optical system is, in my view, one of the most conceptually significant results of the revised manuscript. Highlighting this aspect more clearly, and framing it as a statement about where the nonlinearity can reside in a physical computing system would further strengthen the broader impact of the work.

Finally, the manuscript as a whole is now much better balanced and more careful in its claims. The introduction and discussion are more clear, the figures are more informative, and the connection between experiment, theory, and simulation is substantially tighter. While some of the application-level performance gains remain modest, the manuscript convincingly establishes spatial DOP modulation as a new and potentially powerful degree of freedom for optical information processing. Overall, this is a strong and thoughtful revision, and I again commend the authors for the depth and seriousness with which they have addressed the referees' concerns.

From my perspective, the manuscript's suitability for Nature rests primarily on the conceptual advance of programmable spatial DOP control rather than on the magnitude of the demonstrated ONN performance gains.

Asst. Prof. Matias Koivurova
Center for Photonics Sciences
University of Eastern Finland

Open Access This Peer Review File is licensed under a Creative Commons Attribution 4.0 International License, which

permits use, sharing, adaptation, distribution and reproduction in any medium or format, as long as you give appropriate credit to the original author(s) and the source, provide a link to the Creative Commons license, and indicate if changes were made.
In cases where reviewers are anonymous, credit should be given to 'Anonymous Referee' and the source.
The images or other third party material in this Peer Review File are included in the article's Creative Commons license, unless indicated otherwise in a credit line to the material. If material is not included in the article's Creative Commons license and your intended use is not permitted by statutory regulation or exceeds the permitted use, you will need to obtain permission directly from the copyright holder.
To view a copy of this license, visit https://creativecommons.org/licenses/by/4.0/

Letter to the Referees

We sincerely thank the Referees for their positive and constructive evaluation of our manuscript. Following their comments, we have performed considerable experimental and theoretical work that elevates the paper to a new level. The revised version addresses the key points raised and includes the following major advances:

- Scaling of the modulator to over **1,024 macromodes** modulated in DOP and SOP, representing a two-orders-of-magnitude increase and demonstrating the scalability of the method.

- Implementation of a **new experimental setup with two SLMs,** enabling arbitrary control of the SOP and DOP of each macromode across the beam profile, thereby achieving full programmability of the modulator.

- **Analytical derivation of the DOP modulation** based on coherence theory, strengthening both the foundation and generality of the method.

- **Higher-resolution polarization-colored images** (32 × 32 px) and their encryption.

- Extension of the photonic computation to the **entire CIFAR-10** dataset at its native resolution.

- Development of a **vector model of the PNN** that elucidates the enhancement provided by the high-dimensional encoding.

These points are discussed in detail in the point-by-point response below. Revisions are numbered and indicated in the highlighted version of the manuscript.

Response to Report of Referee #1

Report #1: The authors demonstrate the first programmable spatial modulation of the degree of polarization (DOP) using a phase-only spatial light modulator (SLM) combined with waveplates. While conventional optical systems can independently control phase, amplitude, and the state of polarization (SOP), spatially varying DOP has generally remained inaccessible, limiting applications in high-dimensional photonics. By tailoring the statistical distribution of polarization states across microscopic regions of a beam, this approach enables independent control of both SOP and DOP over macroscopic spatial modes, potentially accessing the full three-dimensional Poincaré sphere. This capability facilitates structured light with enhanced information encoding, as illustrated in two key applications: fully parallel coherent classification of color images mapped from RGB space into a single-wavelength laser beam using a high-dimensional photonic neural network, and secure multidimensional optical encryption leveraging polarization scattering.

This work represents a first-of-its-kind experimental demonstration of programmable spatial DOP modulation, making it both conceptually innovative and practically significant. Prior to this study, optical modulation techniques could manipulate phase, amplitude, and SOP spatially, but no method existed for arbitrary spatial DOP control. Earlier approaches were limited to global or homogeneous DOP or relied on static metasurfaces without per-pixel programmability. The authors address this gap using a statistical approach, engineering the probability distribution of polarization states at a microscopic scale to achieve controlled partial polarization, rather than only deterministic SOP transformations. Notably, this method accesses the full Poincaré sphere, including partially polarized states, opening a new dimension for structured light. The resulting high-dimensional encoding can substantially increase the information capacity of optical fields, demonstrated through both a fully parallel photonic neural network for image classification and multidimensional optical encryption with enhanced security.

Response: We sincerely thank the Referee for underlining the novelty and importance of our work so clearly. In the revised paper, the claims are further supported by new experimental results that, given their importance, have taken their place in the main figures as discussed below.

Comment 1.1: Overall, the manuscript is well written and the results clear. One point that might warrant further discussion is the potential limitation of using only a single tunable phase per SLM pixel in combination with waveplate angles. For a single macromode, the method appears capable of substantial control over both the DOP and SOP, by controlling the phase and phase diversity over the micromodes, and the angles of the waveplates. However, when multiple macromodes are considered, I assume that all of them rely on the same waveplate angles. In this configuration, the SLM introduces a controllable phase delay between the linear polarization components, while

the quarter-wave and half-wave plates provide only a static rotation of the Poincaré sphere. Consequently, for a given coherent input polarization state, the achievable output states as the SLM phase varies may trace only a one-dimensional curve on the Poincaré sphere, rather than spanning the full two-dimensional space of all possible pure polarization states. If this is the case, arbitrary polarization control per pixel could be limited, potentially restricting the spatial diversity of polarization patterns across multiple macromodes. Spatial averaging over multiple phase settings can reduce the measured degree of polarization and simulate partial polarization, but it may not fully overcome the single-degree-of-freedom constraint inherent to the current SLM and waveplate setup.

Response: The Referee's understanding is correct. In the setup made by a single SLM and waveplates, the possible SOPs are constrained over an ellipse on the Poincaré sphere, which can be varied by the waveplate angles (see supplementary Eq. (11)). This limits the possible SOPs achievable across multiple macromodes within a single structured beam at a fixed waveplate configuration, as pointed out by the Referee. To overcome this important limitation, we have implemented our concept by building from scratch a **new experimental setup** that uses two precisely aligned SLMs and no rotating waveplates. The second SLM replaces the waveplates by imparting a tunable phase delay on each pixel. Therefore, in this scheme, control of the DOP applies in the same manner, i.e., by tuning the phase PDF on the first SLM. This is confirmed experimentally and theoretically by the polarization-matrix derivation of the DOP applied to this setup configuration (see supplementary Eq. (14)-(17)). The new setup allows us to **program arbitrarily the SOP and DOP in every macromode**, a remarkable capability that we have exploited for improving our encoding of RGB images in optical computing and encryption (note the new, experimentally measured, polarization-colored images added in Fig. 5a and Fig. 6b). Moreover, the two-SLM setup also allows us to achieve enhanced modulation precision (new Supplementary Fig. 6), being not affected by the error associated with the moving waveplates.

Revision:

- A new section of the Supplementary Information (SUPPLEMENTARY NOTE 4. TWO-SLM IMPLEMENTATION) provides all the details of the additional experimental setup and its theoretical model. (change 1)
- An explanatory photo of the new setup is shown in Supplementary Fig. 4, along with the experimental procedure to align pixel-by-pixel the macromodes of the two SLMs. (change 2)
- Modulation of the DOP and SOP of a single macromode with the two-SLM setup is reported in Supplementary Fig. 5. (change 3)
- Arbitrary control of the SOP per pixel is reported in Supplementary Fig. 6. (change 4)
- The main text has been revised to present the fully-programmable implementation of the modulator. (change 5)

Comment 1.2: In their experiments, the authors show that polarization-based RGB encoding can improve information throughput in an optical neural network. Using a subset of four CIFAR categories, the network achieves 79% classification accuracy when processing polarization-encoded images through optical-only layers with a minimal digital readout. Remarkably, even with an extremely small digital backend of just two hidden nodes, the network reaches 72% accuracy, whereas phase-only grayscale encoding of the same images performs poorly (~51%). These results suggest that encoding RGB information via DOP and SOP allows more information to be carried in a single optical channel, enhancing classification performance in low-digital-complexity optical networks. It should be noted, however, that these benchmarks were obtained on a limited subset of CIFAR within a specialized experimental setup and are not directly comparable to full-scale digital deep neural networks.

Response: The Referee completely grasps the key point: applying the polarization-color encoding in a photonic neural network provides a general approach for enhancing the optical computation by using a new degree of freedom. To further support this key finding, we have taken a significant step towards benchmarking our high-dimensional scheme on the entire CIFAR as implemented in digital neural networks. In addition, we show that our **RGB encoding is not limited to a specialized setup** but applies to other photonic neural networks.

Our experimental results on the complete CIFAR-10 dataset (10 classes) at its native resolution of 32 x 32 RGB px extend significantly those reported in the first manuscript and the state-of-the-art, since **none of the existing optical neural networks process RGB information using parallel multi-dimensional channels**; in addition, they are validated by numerical simulations unveiling the computational advantage provided by the RGB encoding. Specifically, we have developed a model of the high-dimensional optical neural network based on the vector transmission matrices of the optical layers. The model points out the larger expressiveness of the optical transformation in the case of polarization encoding, which is the mechanism behind the improved classification accuracy, and it is in quantitative agreement with the new and complete experimental results.

To demonstrate that results are not restricted to a specialized setup but extend to other neural network architectures, in the revised version, we also report a new experimental implementation based on a different neuromorphic computing paradigm, the so-called extreme learning machine. By applying our polarization encoding in this context, we realize a high-dimensional photonic extreme learning machine (PELM), in which the digital computation and training are even simpler. Note that the PELM scheme can operate with any optical setup that acts as a reservoir, including linear propagation in free space (Ref. [10]) and nonlinear optical systems (Ref. [61]). We observe an improvement in accuracy enabled by the RGB encoding, also for this paradigm. This indicates

that the performance enhancement is not setup dependent. The evidence suggests that our high-dimensional encoding provides an advantage that can be extended to many other PNNs and PELMs, such as those based on metasurfaces, optical fibers, etc.

Revision:

- The previous results on high-dimensional optical computing have been replaced with the new results on the complete CIFAR-10 benchmark, as reported in Fig. 5. (change 6)
- The new section SUPPLEMENTARY NOTE 7.A presents the vector model of the high-dimensional PNN and its simulation with DOP structured light. (change 7)
- Numerical results validating the experimental photonic classification have been added in Supplementary Fig. 13 and Fig. 14. (change 8)
- The new section SUPPLEMENTARY NOTE 7.B discusses the high-dimensional PELM, with experimental and numerical results reported in Supplementary Fig. 15. (change 9)
- We have stressed that, unlike previously reported photonic neural networks, we can process color images fully in parallel because we exploit different optical degrees of freedom, including a statistical quantity alongside deterministic properties. (change 26)

Comment 1.3: The authors further extend speckle-based optical encryption to multidimensional data by encoding RGB images into the DOP and SOP of a single-wavelength beam. The polarization-encoded beam is encrypted through a scattering medium acting as a large, unclonable physical key, with decryption achieved via a small deep neural network that reconstructs the full polarization content from intensity-only measurements. This approach demonstrates high-fidelity reconstruction (SSIM > 0.9) and adds an additional layer of security through the secret RGB to Poincaré mapping. While the scheme significantly increases information dimensionality and physical-layer security compared to prior single-channel optical encryption methods, it remains a proof-of-principle system, sensitive to environmental stability and reliant on obscurity-based keys rather than mathematically guaranteed cryptography. Nevertheless, it represents a state-of-the-art demonstration of multidimensional optical encryption using polarization as an independent encoding degree of freedom.

Response: In the revision, we put a significant effort to go much beyond a proof-of-principle demonstration also for the encryption system; specifically, we moved from the simple images in the first manuscript to 8-bit RGB pictures of real-world standard datasets. In line with the rest of the revised paper, the results on multi-dimensional encryption have been improved by encrypting RGB images at a higher resolution (32 x 32 RGB px). Furthermore, to reinforce the claim about the high security of the scheme, we have conducted an in-depth analysis of the resistance to attacks that achieve a perfect clone of the decryption network or ciphertext.

Revision:

- The previous results on multi-dimensional encryption have been replaced with new results at a benchmark-level resolution of 1024 px, as reported now in Fig. 6. (change 10)
- We have added in Fig. 6c,d the results of the security analysis. (change 11)
- The supplementary section on encryption, now SUPPLEMENTARY NOTE 8, has been consistently updated, including Supplementary Fig. 16 and Fig. 17. (change 12)

Comment 1.4: Overall, the major advance of this work lies in the optical domain: the ability to programmably control the degree and state of polarization at a spatially resolved level opens a fundamentally new degree of freedom in structured light. This represents a conceptually and experimentally significant step forward, and I find it very exciting from the perspective of high-dimensional photonics and optical computing.

Response: We thank the Referee for underlining the impact of our work across photonics. At the same time, we are convinced that exploiting the DOP opens a new frontier at a more general level, as it provides access to a new physical dimension for processing information. As highlighted by the current boom of photonics applications in information and computer science, photonics is standing out as a leading candidate for next-generation information technology. Control over a new degree of freedom in optics directly implicates a new degree of freedom for communication and computing, both classical and quantum. We provide a simple, broadly accessible, high-dimensional computing platform where new computing concepts can be implemented; we believe this impacts the entire research on high-dimensional computing and establishes an exciting link between different scientific communities. With this vision in mind, we have paid special attention to the constructive comments of the Referee and largely revised the manuscript to provide clear evidence that our spatial DOP modulator is beyond the proof-of-principle stage. To make evident how this novel device is of interest to the broad scientific community and can open many new possibilities following what SLMs did in science and industry over the last decade, the revised paper demonstrates the generality of the concept and its immediate impact on different applications (computing, security, imaging) and various setups (two-SLM setup, microlens array setup, different photonic neural networks).

Comment 1.5: At the same time, while the demonstrations are state-of-the-art, they remain proof-of-principle systems and may face practical challenges, including limited macromode diversity, SLM speed constraints, environmental sensitivity, and reliance on specialized experimental setups. Additionally, a more extensive comparison with existing literature could help clarify the relative advantages and limitations of this approach. Nonetheless, these results highlight a promising avenue for optical information processing that could inspire further developments in photonic neural networks, multidimensional encryption, and beyond.

Response: The revised manuscript overcomes many of the practical challenges pointed out, presenting **a device beyond a proof-of-principle** in terms of its scalability, programmability, and computing capability. The theory of DOP modulation that we have added, together with the new demonstration with two SLMs, indicates how the method can be implemented with any spatial phase modulation device, including the most advanced SLM systems (new Ref. [30]), off-the-shelf MEMS SLMs (new Ref. [54]) and emerging SLM platforms (new Ref. [57]) that allow for mitigating the speed constraints of current LCOS SLMs. We have now discussed more carefully these and other aspects, expanding the comparison with the literature as suggested. The additional **new experimental implementation in the Fourier plane** achieved by a microlens array (see SUPPLEMENTARY NOTE 6) demonstrates another promising setup configuration that enables modulating the DOP and SOP in applications that require focusing light, such as nonlinear optics and spectroscopy. Finally, we now provide a general theoretical framework corroborating the advantage of high-dimensional optical computing. As underlined by the Referee, this result is crucial for further developments in photonic computing and beyond.

Revision:

- The introduction and discussion sections have been revised, adding relevant literature:
  [30] He, C., Chen, B., Song, Z., Zhao, Z., Ma, Y., He, H., ... & Forbes, A. (2025). A reconfigurable arbitrary retarder array as complex structured matter. Nature Communications, 16 (1), 4902.
  [54] Rocha, J.C., Wright, T., Butaite, U.G., Carpenter, J., Gordon, G.S., & Phillips, D.B. (2024). Fast and light-efficient wavefront shaping with a MEMS phase-only light modulator. Optics Express, 32 (24), 43300-43314.
  [57] Trajtenberg-Mills, S., El Kabbash, M., Brabec, C.J., Panuski, C.L., Christen, I., & Englund, D. (2024). LNOs: Lithium niobate on silicon spatial light modulator. arXiv:2402.14608.
  (change 13)

Response to Report of Referee #2

Report 2: In this manuscript, Petrini et al. propose an original idea to experimentally generate and leverage optical spatial modes with a programmable degree of polarization (DOP). While control of the state of polarization (SOP) is a mature technique, e.g., using wave retarders, the key innovation here is the introduction of DOP as a tunable degree of freedom. The authors achieve this by defining a "macromode" as an ensemble of "micromodes", where the random phases are drawn from a Gaussian distribution with tunable mean and standard deviation parameters. Combined with polarization wave plates to control individual micromode SOPs, this setup generates a far-field macromode with tunable SOP and DOP. The authors further introduce the concept of "polarization color" and demonstrate its application in optical computing and image encryption.

The underlying physics is clear and interesting. The principle is well-explained, though the manuscript would benefit from a more rigorous analytical derivation of the tunable DOP. While most experimental results are solid, some claims feel overstated, and a few demonstrations are misleading. My detailed comments are provided below.

Response: We sincerely thank the Referee for recognizing the originality of the work and for raising some very constructive issues, which have motivated us to perform new experiments and analyses that have led to remarkable results. Considering this new evidence, we believe that the claims we now make are fully supported, as discussed in detail below. Regarding the Referee's suggestion, we have carried out a rigorous analytical derivation of the DOP resulting from the phase statistics. Starting from the polarization matrix describing the partially coherent beam, we find an expression for ρ (Supplementary Eq. (9)) that fully agrees with the experimental results.

Revision:

- We report in SUPPLEMENTARY NOTE 2. POLARIZATION MATRIX MODEL the analytical derivation of the tunable DOP. The main text has been revised accordingly. (change 14)

Comment 2.1: A significant concern is the potential for crosstalk between macromodes. Since the polarization-structured beam is generated in the far-field, each macromode is essentially a weighted summation of all the micromodes in the SLM plane. Doesn't this mean that adjusting one macromode could inadvertently affect its neighbors, which complicates the independent control of DOP and SOP at a specific spatial location? This needs more explanation.

Response: We agree that we do not sufficiently explain this important point in the original version of the paper. Avoiding any crosstalk between macromodes is crucial for an accurate control of

the spatial modulation of the DOP and SOP, and we have carefully avoided it. To this purpose, first, it is important to select an appropriate propagation distance in the far-field at which the DOP modulation is achieved. This working distance z' depends on the specific sizes of the micromode (*l*) and macromode (*L*) on the SLM plane. To operate correctly, the method requires that several micromodes within a macromode mix by propagation, but without significant spatial overlap with micromodes of adjacent macromodes. Considering that the interaction between two close micromodes and between two close macromodes occurs at a distance of the order of their diffraction length $z_l = \pi l^2/\lambda$ and $z_L = \pi L^2/\lambda$, respectively, the far-field distance must satisfy $z_l \ll z' \ll z_L$. This condition is achieved easily for macromodes of large size, i.e., for $l \ll L$. For example, for L = 2.4 mm and l = 150 µm as in Fig. 3d-f, we obtain approximately 0.1 m < z' < 10 m. In this case, at a distance z' of tens of centimeters, only a minor fraction of micromodes (those on the macromode edge) affects the adjacent macromode, so that crosstalk between macromodes has a negligible effect on the accuracy of the programmed DOP and SOP. Differently, crosstalk becomes relevant when *L* and *l* are closer in value, as occurs when reducing *L* and the number of micromodes *M* to improve the resolution of the modulator. In this case, to avoid macromode crosstalk, **we employ a few blank pixels to spatially separate adjacent macromodes**. The length of this buffer area is chosen appropriately depending on *l* and z', so that micromodes at the edge of two adjacent macromodes have no overlap upon propagation. In the new Fig. 3i, where *l* = 5 px = 60 µm and z' = 5 cm, we use six blank pixels, which is sufficient to avoid any crosstalk. This buffer area is visible in the measured DOP image as a background grid.

An alternative approach to avoid macromode crosstalk without relying on controlling the propagation distance consists of performing the **DOP and SOP modulation in the Fourier plane**. In the revised manuscript, we also explore this configuration in which the far-field plane coincides with the Fourier plane of the SLM**.** The method to control the DOP and SOP remains the same, but, in this experimental implementation, we use an array of microlenses, where each microlens acts precisely on each macromode. By matching the macromode size and spacing with the lenslet pitch, macromode crosstalk is avoided by design, also for macromodes of small size. The polarization-structured beam is now composed of an array of foci of programmable DOP and SOP (see Supplementary Fig. 11 and Fig. 12). Every focal point is the result of a weighted summation of the sole micromodes of the corresponding macromode. This configuration can be preferable in applications that require light to be focused, for example, upon a sample. Considering that an advanced optical component of fixed design (the microlens array) is required for this realization, we focus in the main text on the simpler and more versatile implementation by free-space propagation, where **crosstalk is avoided by blank pixels**.

Revision:

- We have added a section in Methods that explains the effect of a potential crosstalk between macromodes and how we avoid it. (change 15)
- We report the new experimental implementation with the microlens array in SUPPLEMENTARY NOTE 6. SPATIAL DOP AND SOP MODULATION IN THE FOURIER PLANE, which includes the new Supplementary Fig. 10, Fig. 11, and Fig. 12. The main text discusses this additional optical configuration to achieve a polarization-structured beam by the same statistical method. (change 16)

Comment 2.2: The core claim of enhanced high-dimensional encoding is, in my view, not well-justified. Let me elaborate. The method's foundation is the grouping of multiple SLM pixels into a single macromode to define a (theta, chi, rho) tuple for "polarization color" encoding. This polarization color is used to encode a RGB pixel in natural images without using multi-wavelength sources. However, this grouping inherently sacrifices spatial parallelism. For instance, this work demonstrates encoding with N = 4, 25, and 64 macromodes, corresponding to 12, 75, and 192 encoded pieces of information using 3 channels. In contrast, a conventional method using the same SLM without such grouping can encode 4*3600, 25*3600, and 64*3600 pieces of information by treating each pixel as an independent channel (in a similar manner to spatial fan-out). Given that massive spatial parallelism already allows for encoding thousands of channels with a single-frequency laser, the claim that this approach is more "scalable" seems unfair and requires substantial reevaluation.

Response: We thank the Referee for this key analysis, which has motivated us to directly demonstrate the scalability of our approach. We achieve $N = 1024$ macromodes, an improvement of two orders of magnitude with the same setup, which indicates the potential of reaching an even larger scale by high-resolution SLMs. As discussed below, our approach shows that grouping SLM pixels into high-dimensional channels represents an efficient way to encode information, the opposite of what was concluded by the Referee. In fact, a typical limitation of SLM-based optical processors is the need to group pixels to improve the signal-to-noise ratio and the accuracy of operation; as a result, one cannot reach the full scale of the SLM. Our approach turns the need to group pixels into an advantage: by exploiting a statistical property of light, we exploit grouping for adding an extra dimension.

First, we remark that we did not intend to claim that our method for RGB encoding is more scalable than parallel encoding via multiple spatial channels. **The scalability is the same: linear with the number of available pixels** $N = n_{px} / \xi$. The constant factor ξ depends on the grouping of pixels and varies for different encoders. Therefore, it is important to clarify how the grouping of pixels impacts the spatial parallelism of the encoding. We agree that, in general, grouping

sacrifices spatial parallelism. However, the argument sustained by the Referee does not consider a fundamental point: **grouping of pixels is practically unavoidable in any spatial encoding scheme.** Treating the single SLM pixel as an independent channel is challenging even by using optics of large numerical aperture, since it results in a poor signal-to-noise ratio at the detection, and, consequently, in a low accuracy of the encoding. This is the reason why even large-scale spatial encoders exploit macromodes made by several pixels. Our approach provides a method that enables the exploitation of the grouping for adding a dimension. Using macromodes with statistical information instead of a constant phase is a solution that makes the unavoidable grouping procedure useful. The physical space sacrificed by grouping is partially retrieved for encoding information by creating a multidimensional channel. In addition to not losing the linear scalability of spatial parallelism, our approach allows exploiting those pixels lost by grouping by using a statistical property; the grouped pixels are used to tailor a further degree of freedom. In other words, while the two approaches have the same scalability, the advantage of using the polarization lies in the exploitation of the DOP, which is not present when using a simple phase modulation. This makes the encoder high-dimensional and augments the optical processing.

To make the discussion quantitative, we consider Ref. [45], a state-of-the-art paper that addresses the encoding of RGB images (CIFAR at native resolution) by using multiple phase channels on a single SLM. In that work, 5 x 5 pixels are employed for the single piece of information. In our case, at the minimum macromode size ensuring high-accuracy modulation (see new Fig. 3h), a micromode is composed of the same 5 x 5 pixels. Therefore, the total number of SLM pixels for encoding each RGB pixel is comparable in the two cases, only six times larger in our case (625 versus 100).

Considering the significantly scaled-up version of the modulator and the above discussion, we have carefully balanced and made precise our claim on the scalability of the high-dimensional encoding: the scalability is the same as using multiple spatial channels. The revised manuscript discusses the scaling with the number of SLM pixels and simply states that **the approach is scalable**, which is fair and well justified by the data.

Revision:

- We have scaled up the modulator, reaching $N = 1024$ macromodes that operate with high accuracy. The large-scale implantation is shown in the new Fig. 1e. The scaling analysis by varying the size and number of micromodes is reported in the new Fig. 3h. (change 17)
- Experimental measurements that demonstrate the polarization-color encoding of 32 x 32 RGB images with high fidelity are reported in the revised Figs. 4,5, and 6 of the main text and supplementary material. (change 18)
- We have discussed the scalability with respect to the number of SLM pixels. (change 19)

Comment 2.3: What is the physical size of a formed macromode in the far field compared to the size of a micromode in the SLM plane? This information is important for understanding the spatial resolution of the system.

Response: Following the response to Comment 2.1, in the revised version, we have presented two different setups (free-space propagation and Fourier plane) that differ in the location of the target modulation and are engineered for different applications. The size of the macromode can be tailored in both designs. In the free-space configuration, the working distance is much smaller than the diffraction length of the macromode. Therefore, the physical size of the macromode in the far field is approximately the same as on the SLM plane, namely L. At the maximum modulation resolution of 32 x 32 macromodes, we have L = 25 px = 300 μm, while the size of the micromode on the SLM plane is l = 60 μm. Scale bars indicate the spatial resolution in all the experimental images. In the Fourier-plane configuration, the size of a formed macromode is determined by the specifications of the microlens array. In our case, we have a lenslet pitch of 300 μm and focal length f = 14.2 mm, which gives macromodes (focused) with a size of approximately 0.05 mm in the Fourier plane (see Supplementary Fig. 11).

Revision:

- Besides scale bars in figures, we specify in Methods the size of the formed macromode. (change 20)

Comment 2.4: The application in optical computing leverages a complex medium, which connects to the field of physical reservoir computing. A key principle in reservoir computing is the use of a simple, linear digital backend for easy training. However, the authors employ a two-layer MLP as the digital backend. Though this may boost performance, it complicates the training process and undermines the core advantage of reservoir computing. In addition, the claim of “surpassing the performance of state-of-the-art deep optical neural networks [7]” feels disingenuous when a nontrivial digital network is doing much of the heavy lifting.

Response: We agree with the Referee. Following the comment, we have performed new experiments that make use of a simpler linear digital backend. This scheme realizes a high-dimensional photonic extreme learning machine with a random reservoir. It shows that polarization encoding directly applies to optical reservoir computing and potentially other optical neural networks. Results on the CIFAR-10, corroborated by numerical simulations, confirm the enhancement observed for the RGB encoding, further indicating that this enhancement is a property of the optical processing.

We also agree on the claim about the performance comparison with Ref. [7], which has been properly amended.

Revision:

- The photonic computation by using only a linear digital layer has been added in SUPPLEMENTARY NOTE 7. B. HIGH-DIMENSIONAL PELM, with experimental and numerical results reported in Supplementary Fig. 15. (change 9)
- The PELM implementation is discussed in the main text. (change 21)
- The imprecise claim on the PNN performance has been corrected. (change 22)

Comment 2.5: Figure 5a schematically illustrates optical encryption using a high-resolution van Gogh image, creating a strong impression. However, the actual experimental results in Fig. 5b are all low-resolution plain texts. This discrepancy is misleading and should be corrected. The schematic should accurately reflect the scale of the experimental validation. Furthermore, the authors should discuss a potential drawback of their method: compared to conventional speckle-based encryption using fully coherent light, the use of partially coherent light will smoothen the speckle contrast. This likely makes the ciphertexts for different plaintexts more similar, potentially degrading the decryption quality, which should be acknowledged.

Response: Although Fig. 5a was only a sketch intended to illustrate the encryption principle, we understand that it may disorient the reader, and we have corrected it. The illustration now reports one of the 32 x 32 RGB images used as a plaintext in the new experiments. The encryption of these higher-resolution images significantly improves the encryption experiments of the first version (see also Supplementary Note 8 for other new results).

We agree that our encoding via partially-polarized light results in speckle patterns with reduced contrast. This is a relevant effect that we have experimentally verified, finding that a single beam with $\rho < 1$ gives a measured speckle contrast $C \propto \sqrt{\rho}$ as expected from a partially-polarized beam (see, for example, Chapter 4 of J.W. Goodman, Speckle phenomena in optics, II Ed). As noted by the Referee, the effect makes the ciphertexts for different plaintexts more similar, making the decryption by the neural network more demanding. However, a quantitative comparison of the decryption accuracy against fully coherent speckle-based encryption is not possible since RGB images can be encoded only with partially polarized light. Note that a smoothed speckle contrast also makes the encryption more secure. The higher level of precision required during training protects against imitation learning attacks, i.e., the training of an equivalent network based on a stolen set of plaintext-ciphertext couples. The security against cloning (almost completely) of the decryption network is now analyzed in Fig. 6c.

Revision:

- Fig. 6a has been corrected. Previous results on multi-dimensional encryption have been replaced with new results at a higher resolution in Fig. 6b. (change 10)
- We have added in Fig. 6c,d the results of the security analysis. (change 11)
- The supplementary section on encryption, now SUPPLEMENTARY NOTE 8, has been consistently updated, including Supplementary Figs. 16, 17. (change 12)
- We have noted the effect of a reduced speckle contrast on the decryption. (change 23)

Comment 2.6: Referring to the number of diffusers as "optical depth" is misleading, as this term is strongly associated with neural network layers. The diffusers are linear optical systems. The manuscript should use a more precise term. Besides, how to understand that the performance enhances with more diffusers since they are linear?

Response: As suggested, we have now avoided referring to the number of diffusers as optical depth, since these represent linear optical layers. The accuracy enhancement observed by cascading more diffusers is an interesting and counterintuitive effect considering the linearity of the optical transformation performed by each scatterer. We have identified its origin in the polarization response of the thin diffusers that are cascaded, as detailed below. Therefore, this **enhancement is a specific feature that emerges due to the polarization processing and has no scalar counterpart.**

To understand the effect, we have developed a model of the high-dimensional PNN in which each diffuser is represented by a vector transmission matrix (VTM). A VTM with random complex coefficients is known to model the optical transformation performed by a scattering medium on an input field with arbitrary polarization in each input mode. We have realized the propagation of a beam structured in DOP and SOP in this framework by a proper decomposition of the field into its orthogonal coherent components, as detailed in SUPPLEMENTARY NOTE 7.A. Numerical simulations of the model allow us to investigate how the structure of the VTM describing the polarization response of the optical layer affects the computation. We find that the amplitude of non-diagonal submatrices of the VTM directly impacts the performance. These sub-matrices determine the expressiveness of the mapping between the input polarizations and the output intensities. To understand this fact, consider that an ideal complex medium whose scattering does not alter the polarization is described by null sub-matrices and has only $K \times G$ complex random coefficients, being K and G the number of input and output modes. Thin scatterers, such as the ground-glass diffusers employed, are characterized by small non-diagonal coefficients compared to the diagonal ones, which means the scattering process causes small polarization rotations. When cascading many of these diffusers, the effect of the scattering on the polarization accumulates. The overall transformation approaches the one provided by the VTM of a thick

medium, which comprises $2K \times 2G$ random elements with diagonal and non-diagonal coefficients of comparable mean amplitude. Therefore, by using more diffusers, we are progressively increasing the rank of the optical transformation. This effect is a specific feature of the polarization operation. Consistently, we do not observe it for phase encoding and scalar propagation. Considering that the effect represents an original aspect of the high-dimensional computation, we have now reported in Fig. 5c its observation on the CIFAR-10 classification, comparing experimental and simulation results. Revisions on the optical computing part are listed below.

Revision:

- The term "optical depth" has been removed. Fig. 5c has been added to demonstrate the enhancement with the number of diffusers. The origin of the effect has been discussed in both the main text and the supplementary. (change 24)
- The previous results on high-dimensional optical computing have been replaced with the new results on the complete CIFAR-10 benchmark. (change 6)
- The new section SUPPLEMENTARY NOTE 7.A presents the VTM model of the high-dimensional PNN and its simulation. (change 7)
- Numerical results on the PNN have been added in Supplementary Figs. 13, 14. (change 8)

Comment 2.7: If information is encoded in the defined polarization color space (theta, chi, rho), is the mapping from this parameter space to the output vector field after the diffusers linear or nonlinear?

Response: The mapping is nonlinear. In particular, it is the encoding of the RGB information in the input field that is nonlinear. Note that, in response to the comments of the other Referees, the results presented in Fig. 5 of the revised paper are obtained by using an equivalent RGB to polarization mapping in which the color is defined within the Poincaré sphere by the vector (S_1, S_2, S_3). In this case, the encoding is nonlinear as the Stokes parameters are quadratic functions of the field components.

Revision: We have added a comment to clarify this point. (change 25)

Comment 2.8: Supplementary Information, there is a typo in "divided into N square macromodes composed of N micromodes".

Response and revision: Typos have been corrected. The whole text has been carefully reviewed.

Comment 2.9: Overall, while I find the core idea of generating tunable DOP original and compelling, the potential advantages of the proposed approach are, in my opinion, overexaggerated. This work represents a specialized advance in optics and photonics. And it does not seem to have the broad, interdisciplinary appeal required for Nature. It would be a better fit for a more specialized journal such as Nature Photonics, Nature Physics, Physical Review Letters, or Nature Communications.

Response: We are pleased that the Referee finds our idea original and compelling. The revised manuscript concretizes the concept at a higher level of maturity with remarkable new results, conveying more effectively the advantages enabled by the spatial modulation of the DOP in optical information processing.

Since **the approach enables control of a new degree of freedom of light**, we are convinced that the advance is significant beyond optics and photonics. In fact, control over a new degree of freedom in optics directly implicates a new degree of freedom for communication and computing, both classical and quantum. This fact is highlighted by the current boom of photonics applications in information and computer science, which makes optical systems a leading candidate for next-generation information technology. We provide a simple, broadly accessible, high-dimensional optical platform where new computing and information concepts can be readily implemented. Therefore, the impact of such a platform is transversal.

Having in mind the progress that SLMs have enabled in science and industry over the last decade, we believe the spatial DOP modulator can open new possibilities also beyond the applications that we have demonstrated, which makes the advance reported of broad appeal. Our device is the first-of-its-kind, enabling control over an unexplored degree of freedom, and, therefore, its impact on other disciplines can go beyond what we can foresee. Moreover, the new concept of the polarization color impacts common knowledge, and this makes it of general interest.

Response to Report of Referee #3

Report 3: I co-reviewed this manuscript with one of the reviewers who provided the listed reports.

Response: We thank the Referee for the careful review of our manuscript. The Referee can find the relevant discussions and changes in response to the corresponding report.

Response to Report of Referee #4

Report 4: The manuscript titled "Spatial degree-of-polarization modulation for high-dimensional optical computing and encryption," by A. Petrini, C. Conti, and D. Pierangeli introduces a method for programmable spatial modulation of the degree of polarization (DOP) using a phase-only spatial light modulator (SLM) and some polarization optics. By tailoring the statistical distribution of the phase across multiple micromodes, the authors achieve independent control of both DOP and state of polarization (SOP) across a smaller number of spatial regions of an optical beam, which they call macromodes. This enables the generation of structured light with arbitrary spatially varying polarization states that lie inside the Poincaré sphere. The authors demonstrate two applications: (i) encoding RGB images via "polarization colors" and processing them with a high-dimensional optical neural network (ONN), and (ii) secure optical encryption of multidimensional data using polarization-encoded speckle patterns.

The manuscript cites recent and relevant literature, including foundational works in optical computing, polarization optics, and optical encryption. The abstract and introduction are dense and somewhat repetitive. The abstract should more clearly distinguish the novelty from the applications. The introduction could be streamlined to focus on the technological gap and the contribution of this work. Figures are central to understanding the results but require clearer labeling and integration into the text. The conclusions are ambitious.

Response: We sincerely thank the Referee for the valuable report that has prompted us to make further progress in applying our method and achieve new remarkable results. This additional work has led to a considerably improved manuscript. In this revised version, following the Referee's suggestions, the abstract and introduction have also been carefully revised to convey more clearly the key points. The abstract now clearly distinguishes the novel concept from its applications, while the introduction avoids repetitions and underlines the scientific gap filled by the work. All the figures have been improved, both in terms of the result presented and the quality and clarity of their presentation.

Revision:

- The abstract and introduction have been revised. (change 26)
- Figures have been revised with particular attention to labels and captions. (change 27)

Comment 4.1: The core novelty lies in the programmable spatial control of DOP, which has not been previously demonstrated in a scalable and reconfigurable manner. The method is elegant and leverages existing SLM technology, making it broadly accessible. However, I believe that the claim that RGB encoding is not possible using SOP alone is overstated. There appears to be no

reason why a direct mapping from the Stokes parameters (S1,S2,S3) to (R,G,B) is not feasible. In fact, polarization imaging is the same process put in a different direction, that is, mapping color to the SOP. The authors should either refute my reasoning, or revise this claim and clarify that DOP adds an additional degree of freedom, rather than being strictly necessary for RGB encoding.

Response: We thank the Referee for raising this crucial point that was not clear enough. To our understanding, **the DOP is strictly necessary to create a one-to-one mapping** between RGB colors and polarization states. In fact, the encoding of arbitrary RGB colors requires three independent channels. It is the complete independence of these channels the key point that allows for achieving a genuine color. In the RGB model, any color is expressed as a linear combination of the three primary colors. The SOP alone cannot provide this essential property because the Stokes parameters of fully-polarized light are not independent but constrained on the surface of the Poincaré sphere. Suppose you create a set of colors by association with different SOPs. Although this appears workable, these are not genuine colors because you cannot properly combine them to get another color. For example, whatever you define the red, green, and blue on the Poincaré sphere surface, the white [1,1,1], expressed as a linear combination of the three, would be unavoidably outside the sphere. This argument can be put in more general terms by stating that a volume cannot be made equivalent to a surface, i.e., it is mathematically not possible to perform a continuous transformation from 3D to 2D that is one-to-one.

To make this important point clear, we have now adopted a mapping that is easier to visualize and understand. Each point (R,G,B) is linearly mapped to a point of Stokes coordinates (S1, S2, S3) so that the RGB cube is inscribed inside the Poincaré sphere. The new Figure 4a illustrates and exemplifies this mapping. It is evident that, in order to properly map the volume (cube) defining the color space, a volume in Stokes space is required, i.e., the degree of polarization is necessary.

Revision:

- We have adopted a mapping that makes clear why the DOP is necessary for encoding the color in polarization. The new results presented in the revised paper are obtained through this mapping. We have added a new figure, Figure 4, that illustrates the mapping between the RGB to Stokes space and shows a measured polarization-colored image. (change 28)
- We have discussed polarization colors in a dedicated section, also explaining the reason why the SOP is insufficient for a one-to-one mapping of the RGB space. (change 29)

Comment 4.2: The integration of DOP modulation into ONNs and encryption systems is compelling, but the broader impact may be limited unless the authors can demonstrate scalability, robustness, and generalizability beyond the specific experimental setup.

Response: To comply with the reviewer criticisms, in the revised manuscript, we put a significant effort in performing additional experiments, including a new experimental setup, which clearly show scalability, robustness, and generalizability. We have achieved a scaled-up version of the modulator with over 1024 macromodes. The results on both optical computing and encryption now reflect this major improvement. The scale and accuracy of our high-dimensional photonic neural network now pair with state-of-the-art realizations. However, we stress that existing photonic neural networks require multiple channels to process multi-dimensional data, with the major drawback of performing serial operations; on the contrary, our method is intrinsically parallel in treating three-dimensional input data. Importantly, the DOP modulator can be integrated into many other types of ONN. To provide an example, the revised paper also reports the application to a photonic extreme learning machine (PELM), a paradigm in reservoir computing. This broad applicability ensures the impact on multiple communities, opening the route to optical computing and encryption in a higher dimension. Crucial for this broad applicability is the easy accessibility of our method, which uses only commercially available SLMs, as underlined by the Referee.

In the revised paper, major progress has also been made as regards the robustness and **generalizability to other platforms**. The fully programmable experimental setup made by two SLMs (see SUPPLEMENTARY NOTE 4. TWO-SLM IMPLEMENTATION) and the Fourier-plane implementation with a microlens array (SUPPLEMENTARY NOTE 6. SPATIAL DOP AND SOP MODULATION IN THE FOURIER PLANE) shows that the concept can be engineered at will. The robustness of the method has been assessed by analyzing and controlling the crosstalk between the macromodes. In encryption, we now demonstrate the robustness to adversarial attacks by showing resilience up to 99% of hacking of the decryption network, as detailed in response to Comment 4.4. We list below these key revisions to the manuscript, made also in response to the other Referees, which pertain to the realization of the scaled-up version of the modulator and its integration into optical computing and encryption systems.

To summarize, we have demonstrated: (i) generalizability by applying the concept to different setups (free-space and Fourier plane) for complementary applications, and different neuromorphic architectures (deep as the PNN, and shallow as the PELM); (ii) scalability by enlarging the modulator resolution by two orders of magnitude; (iii) robustness in the target modulation, by controlling macromode crosstalk, and robustness in the encryption, by showing high security to strong attacks.

Revision:

- We have scaled up the modulator to 1024 macromodes. The scaling analysis by varying the size and number of micromodes is reported in the new Fig. 3h. (change 17)

- Results that demonstrate the polarization-color encoding of 32 x 32 RGB images are reported in the revised Figs. 4,5, and 6 of the main text and in the supplementary material. (change 18)
- The supplementary material reports the experimental implementation (Supplementary Fig. 4) of the fully-programmable DOP and SOP modulator, along with its characterization. (Change 1) (Change 2) (Change 3) (Change 4)
- The previous results on high-dimensional optical computing have been replaced with new results on the complete CIFAR-10 (32 x 32 RGB images), as reported in Fig. 5. (change 6)
- The new section SUPPLEMENTARY NOTE 7.B discusses the high-dimensional PELM, with experimental and numerical results reported in Supplementary Fig. 15. (change 9)
- The previous results on multi-dimensional encryption have been replaced with the new results at a higher resolution, as reported in Fig. 6. (change 10)
- We report the experimental implementation with the microlens array in SUPPLEMENTARY NOTE 6. SPATIAL DOP AND SOP MODULATION IN THE FOURIER PLANE. The main text discusses this alternative optical configuration to achieve a polarization-structured beam by the same statistical method. (change 16)

Comment 4.3: The experimental methodology is sound and well-documented. The use of Gaussian phase distributions to control polarization statistics is well-justified, and the calibration of the modulator is thorough. The authors provide quantitative validation of their control over DOP and SOP, including polarimetry and full-Stokes imaging. The encryption and ONN experiments are supported by appropriate datasets and performance metrics. However, the manuscript would benefit from a more rigorous treatment of coherence. The authors describe the optical processing as "coherent," yet DOP is directly linked to partial coherence. That is, the DOP sets a limit to the maximum attainable degree of coherence [J. Opt. Soc. Am. A 33, 2431-2442 (2016)], which contradicts the claim of coherent processing. This should be clarified, especially in the context of the ONN, where coherence may affect interference and propagation through scattering media.

Response: We thank the Referee for noting this important point. The comment has motivated us to perform a theoretical study of our method based on coherence theory. In Supplementary Note 2, we report a rigorous derivation of the DOP as resulting from the phase statistics. Starting from the polarization matrix and averaging over the ensemble of micromodes, we arrive at the expression for ρ (Supplementary Eq. (9)) that we have found experimentally. Notably, the derivation is entirely analytical.

As regards the coherence of the optical processing, we agree with the Referee. As noted, the optical processing is partially coherent. We have now treated this property explicitly to simulate

the high-dimensional PNN and the propagation of DOP-modulated light through the scattering media (see the response to Comment 4.6). In simulations, we have decomposed the partially coherent field of each macromode into orthogonally polarized coherent components. Overall, the revised manuscript contains a clear and rigorous assessment of the theoretical aspects, showing quantitative agreement with experiments.

Revision:

- The statement on coherence has been corrected, clarifying that the optical processing is partially coherent. We consider the suggested reference to be important for this aspect, and we have added it to the bibliography. (change 30)
- We report in SUPPLEMENTARY NOTE 2. POLARIZATION MATRIX MODEL the theoretical treatment of our method. The main text has been revised accordingly. (change 14)

Comment 4.4: The authors report classification accuracy, RMSE for polarization control, and SSIM for encryption fidelity. These metrics are appropriate, but the statistical treatment could be strengthened. For example, confidence intervals or standard deviations across multiple trials would help assess robustness, although this is not a major concern. The encryption system's resistance to noise and adversarial attacks is briefly mentioned but not rigorously analyzed.

Response: We have now put particular attention to these aspects. Confidence intervals are reported in all cases where they are relevant. In cases where multiple trials are performed but errors are not reported (e.g. Supplementary Figure 2), it means they are within the symbols.

The security of the encryption has been analyzed in more detail to further reinforce this claim. The new results in Fig. 6c and 6d, which refer to encryption-decryption of high-resolution RGB images, show the resistance to two different high-level adversarial attacks that achieve an almost perfect clone of the cryptosystem. In the first, the attacker achieves a perfect clone of the ciphertext but lacks a tiny amount of information about the decryption network. In the second, the network is cloned perfectly, but the ciphertext presents a minor systematic error (a few-pixel misalignment). In both cases, the tiny amount of lacking information is sufficient to prevent unauthorized decryption. To quantify the decryption accuracy, in the revised manuscript, we choose to use the Pearson correlation coefficient (PCC), as this metric is more interpretable and can be applied beyond images. The resistance to noise is also addressed more clearly in the new Supplementary Fig. 16.

Revision:

- We have added new results on the cryptosystem security in Fig. 6c, d. (change 11)

- The supplementary section on encryption, now SUPPLEMENTARY NOTE 8, has been consistently updated, including Supplementary Fig. 16 and 17. (change 12)

Comment 4.5: The conclusions are generally supported by the data, but some claims require attention. The assertion that DOP modulation enables RGB encoding seems to be an overstatement. The claim of coherent processing is not correct.

Response: As discussed, the claim about polarization colors has a solid basis. It is a crucial aspect of the paper. The statement on the coherent processing, which did not alter the conclusions of the work, has been properly corrected thanks to the Referee's note.

Comment 4.6: The ONN performance is promising, and the improvement over grayscale encoding is well-demonstrated. However, the underlying reason for this improvement is not fully explored. Here, it seems that the inclusion of the DOP is a key factor, although there is no concrete data to support this idea. A deeper analysis of the improvement mechanism would strengthen the manuscript.

Response: We have now investigated in depth the improvement observed with the RGB encoding by developing a model of the high-dimensional PNN and performing numerical simulations of the experiment. Numerical results (Supplementary Fig. 13) further support the result and have allowed us to identify the underlying reason for the enhancement provided by the encoding in DOP and SOP. This performance enhancement comes from two factors. The first is general: colors provide additional information about the scene, helping to identify the class by their correlation with the object (e.g., the sky is often blue). The second mechanism is more interesting. It is a **mechanism that arises from the vector nature of the field** through the high-dimensional transformation performed by the optical layers. Specifically, the polarization-encoded input is processed by a complex matrix that has a larger rank than its scalar counterpart, since it accounts for the polarization response of the medium. This is evident in the framework of the vector transmission matrix (VTM), which is used to model the cascaded diffusers that form our optical network. By the polarization encoding, the single optical layer passes from being expressed by $K \times G$ to $2K \times 2G$ complex coefficients, where K and G refer to the input and output spatial modes. This larger expressiveness explains the superior performance of the high-dimensional PNN. The model is detailed in SUPPLEMENTARY NOTE 7. Interestingly, numerical simulations also reproduce the accuracy improvement observed as more diffusers are stacked (new Fig. 5c). As detailed in SUPPLEMENTARY NOTE 7, this enhancement also originates in the VTM of the diffusers. It is another specific feature that emerges due to the polarization processing and has no scalar counterpart.

Revision:

- The underlying reason for the computing enhancement has been identified and discussed in both the main text and supplementary information. (change 31)
- The new section SUPPLEMENTARY NOTE 7.A presents the VTM model of the high-dimensional PNN and the numerical simulations. Numerical results have been added in Supplementary Fig. 13 and 14. (change 7) (change 8)

Comment 4.7: In it's present form the manuscript is not suitable for publication in Nature. However, it may be suitable after a revision where the following concerns are addressed:
1. Clarify the role of DOP vs SOP in RGB encoding and revise claims accordingly.
2. Reformulate the role of coherence.
3. Provide a deeper analysis of the ONN improvement.
Additionally, it would be desirable to explore scalability, e.g., higher-resolution images, faster modulation rates, or integration with metasurfaces.

Response: We thank again the Referee for their constructive position. All three concerns have been addressed concretely with new results. To summarize:

1. The necessity of the DOP for a genuine encoding of RGB colors in polarization has been clarified. A mapping that exemplifies this crucial point has been used in the revised paper. The figure and text on this aspect have been carefully revised by adding new results.

2. The role of coherence has been properly reformulated, also adding a theoretical derivation of our method based on the theory of coherence and polarization.

3. The advantage of high-dimensional computing is now analyzed by developing a model of the optical neural network, and it is strongly supported by numerical simulations. The origin of the effect is associated with the optical information processing based on SOP and DOP.

The revised paper also convincingly demonstrates scalability, high-resolution polarization-colored images, and other experimental setups, also in response to the other Referees.

Response to the second report of Referee #1

Report #1: The authors have done substantial new work in response to my comments. The scaling demonstration to N = 1024 macromodes, the introduction of a two-SLM fully-programmable setup, the analytical derivation based on coherence theory, the VTM-based model of the PNN, and the move to full CIFAR-10 at native resolution all represent real progress. Several of my specific concerns are now addressed convincingly. However, some of my more fundamental objections, particularly regarding the scalability framing and the broader-impact claim, are responded to with clarification and reframing rather than with evidence that changes my view. Especially, on the question of suitability for Nature, my position is unchanged, though I recognize this is ultimately an editorial judgment rather than a technical one. The work is excellent and would in my view be very strong for Nature Photonics or Nature Communications.
Below I give a point-by-point response to the comments that I am not fully satisfied with.

Response: We thank the Referee for recognizing the significant progress achieved in the revised manuscript and for providing valuable comments that motivated many of these improvements. Following the Referee's suggestions, we have further improved the manuscript by addressing the points raised here.

Comment 2.1: I am mostly satisfied with the response of the authors but I do have one follow-up. The diffraction-length argument is sound and the buffer-pixel mitigation strategy is a reasonable engineering solution. The Fourier-plane microlens-array implementation is an elegant alternative that essentially eliminates the problem and I very much appreciated seeing it.
One follow-up, though: the buffer pixels are presented as a small overhead for larger macromodes, but at the minimum-size regime (L= 25 px, six blank pixels per side) the buffer footprint is not negligible. It would be interesting to add to the supplementary material the effective fill factor at different resolutions and quantify residual crosstalk experimentally e.g., by programming a single bright macromode against a uniformly dark background and reporting the measured DOP "leakage" into neighbours. In this way, readers can assess how negligible the crosstalk is in a quantitative way.

Response: We agree that the buffer footprint is not negligible at maximum resolution. Supplementary Fig. 18a shows the fill factor for different macromode sizes. For L=25 px and six buffer pixels per side (d=6 px), the effective fill factor is approximately 0.65. This value can be increased to 0.75 by reducing the buffer width to d=4 px, without introducing significant crosstalk. As suggested, we have experimentally quantified the residual crosstalk (Supplementary Note 9). To isolate the effect, we program two neighboring macromodes within a uniformly polarized background. We vary the SOP and DOP of one macromode (the control macromode) and measure

the deviation induced in the neighboring polarization state (the reference macromode) from its target value. The method is illustrated in Supplementary Fig. 18b,d through polarization images of the SLM plane.

We first quantify the crosstalk effect on the SOP. The results in Supplementary Fig. 18c show that a macromode separated by d=6 px induces a negligible error in the Stokes parameters of its neighbor. The measured relative error of 0.015 (averaged over different SOPs) is comparable to that obtained for an isolated macromode. The error remains small even for d=4 px, indicating that the buffer width can be reduced to this value without significantly compromising modulation accuracy. DOP leakage is quantified in Supplementary Fig. 18e for d=5 px. In this analysis, we measure the DOP of the reference macromode while varying the DOP of the neighboring one. The relative error on ρ remains below 0.08. We note that approximately half of this value arises from the intrinsic uncertainty of the setup in modulating the DOP of an individual macromode (calibration in Supplementary Fig. 5c). Therefore, DOP leakage contributes at most an additional error of 0.04 and remains well below this value throughout most of the DOP range.

Comment 2.2: This was my central concern and remains a point where I still have the most reservation. I especially like that the 100x scaling to 1024 macromodes is a real demonstration, rather than just a promise. I feel the reframing by the authors is honest: the linear scaling with pixel count is the same as for conventional spatial encoders, and the advantage lies in the kind of information encoded per macromode rather than in raw channel count. The comparison with Ref. [45] is a fair quantitative benchmark, although the 625 vs 100 pixels argument is unclear in their response and would need some clarification. A direct table comparing pixels-per-encoded-RGB pixel across the relevant prior work would be a great addition to the supplementary material to strengthen their point.

Response: We are pleased that the Referee agrees with our assessment of the technical aspects of this point. Regarding the discussion on scalability, we emphasize that our objective in the context of optical computing is not to demonstrate an advantage in raw channel count, as the number of pixels is a readily available resource in SLM technologies. Rather, the main advance enabled by our approach is conceptual, consisting of a different paradigm for optical information encoding and processing. Nevertheless, we agree that scalability is an essential requirement, and we have provided a robust demonstration of the system's scalability. In this respect, the linear scaling with the number of pixels indicates that progressively higher resolutions can be achieved without introducing fundamental scaling limitations, requiring only further engineering of the experimental setup. This scaling law is analogous to that of conventional spatial encoders, and our quantitative argument in the previous response (625 vs 100) showed that the required pixel count is also of the same order of magnitude. This comparison with Ref. [45] concerns the total number of SLM pixels required to encode by spatial multiplexing a single RGB pixel of a CIFAR

image. Following the Referee's suggestion, we have clarified this comparison by adding a dedicated table in Supplementary Note 10, which also includes other previous works that encode comparable RGB information using SLMs. In these works, the entire SLM is typically used to encode images of resolution comparable to CIFAR images, further supporting our argument for an analogous pixel count.

Comment 2.2: The wording in the revised text and abstract still uses "high-dimensional" in a way that can be interpreted as the distinction between (a) genuinely accessing a new physical degree of freedom (which is the case) and (b) increasing information density per encoded channel (which I understand now holds only modestly). I suggest the authors tighten the language or clarify exactly what is meant with "high-dimensional".

Response: We thank the Referee for highlighting this point, which is important for ensuring clarity for a broad audience. We have refined the language throughout the manuscript, paying particular attention to avoiding any confusion between the system dimensionality and information density per channel. The term "high-dimensional" is used in its proper physical sense, namely to indicate the inclusion of an additional degree of freedom that provides access to a high-dimensional space ($D > 2$). This definition is now stated explicitly in the introduction with relation to the three-dimensional Poincaré sphere.

Comment 2.6: I am mostly satisfied with the response of the authors. Removing "optical depth" is appropriate. The VTM-based explanation, i.e. thin diffusers have small off-diagonal coupling, so cascading progressively populates the full 2N x 2N polarization-mixing matrix, is physically reasonable and is now backed by Fig. 5c showing experimental and simulated agreement.
Nevertheless I remain with a small concern: in the supplementary material the authors mention a saturation which should occur within a small number n=4. Does the experimental trend in Fig. 5c saturate within the n = 1–4 range shown, and is this consistent with the simulated VTM? Showing this saturation explicitly (or extending to n = 5–6) would be very convincing.

Response: The experimental behavior in Fig. 5c saturates at n=4. The accuracies for n=4 and n=3 are indistinguishable within the statistical uncertainties. We have revised Fig. 5c (now Fig. 4e) to make the saturation effect more evident as suggested. This is consistent with the simulations, although numerical results still show a slight improvement at n=4, and the saturation occurs at n=5. This minor discrepancy arises from the fact that diffusers used in the experiments have different grit polishes, a property that can be represented only approximately in the VTM model.

Comment 2.6: The authors have produced a genuinely improved manuscript. It shows that the demonstrator is more scalable. The results are now more rigorous theoretically and

experimentally more complete. I now agree that the work goes meaningfully beyond proof-of-principle in several respects. However, my view on journal fit was primarily about the breadth of the conceptual advance. Spatial DOP modulation is a real and elegant new capability, and the polarization-color framing is a genuinely nice idea. The applications shown (one-shot RGB classification on CIFAR-10 at 50.4%, multi-dimensional speckle encryption) are solid demonstrations within optics but do not establish the kind of cross-disciplinary impact that, in my judgment, distinguishes a Nature paper from an excellent specialized one. The authors' argument that "control of a new optical degree of freedom directly implicates a new degree of freedom for communication and computing" is reasonable in principle but is asserted rather than demonstrated. The CIFAR-10 accuracy is competitive with prior multi-channel PNNs rather than disruptive of any computing benchmark.

I therefore maintain that the natural home for this work is Nature Photonics, Nature Communications, or a similar venue, where the optics community will appreciate the genuine novelty.

Response: We thank the Referee for the positive assessment of the revised manuscript. We respect the Referee’s view regarding the cross-disciplinary impact and journal fit. While views on these aspects may naturally differ, we are grateful for the thoughtful and constructive feedback provided throughout the review, which has helped strengthen the work considerably.

Response to the second report of Referee #2

Comment 2.1: The authors have clearly explained that the encoding of the RGB image in the input field is nonlinear. I note that in the "Encoding colors in polarization" subsection of the Methods, the authors use the phrase "the linear map". While this is technically correct when referring to the relationship between S1/S2/S3 and R/G/B, using the word "linear" in this context might cause confusion. I suggest simply deleting the word "linear" here or replacing it with another term.

Response: We thank the Referee for the very appropriate comment. We have removed the word "linear" when referring to the polarization-color mapping, as suggested.

Comment 2.2: Does the nonlinearity of the encoding scheme contribute to the enhanced performance of the high-dimensional PNN? I suggest adding a brief discussion to clarify this point.

Response: This is an interesting aspect. To our understanding, while the nonlinearity of the encoding contributes to the overall performance, it does not play a key role in providing the accuracy enhancement with respect to the corresponding network with phase encoding. The reason is that data encoding is nonlinear also in phase-based schemes, and different nonlinear encoding strategies are known to lead to comparable accuracies (Li et al., Nonlinear encoding in diffractive information processing using linear optical materials, Light: Science & Applications 13, 173, 2024). We have added a sentence in Methods to clarify this point.

Response to the second report of Referee #4

Report 4: I would like to commend the authors for the exceptionally thorough and serious revision of the manuscript. The revised version reflects a substantial amount of new experimental, theoretical, and numerical work, well beyond what is typically expected in a revision. In particular, the scaling of the spatial DOP modulator to 1024 independently programmable macromodes, the introduction of a fully programmable two-SLM implementation, and the addition of a rigorous coherence-theory and vector transmission matrix (VTM) framework significantly strengthen the manuscript. The revised manuscript is clearly more mature, more careful in its claims, and technically much more solid than the original submission.

The central conceptual contribution of the work remains the introduction of the degree of polarization as a genuinely new, programmable spatial degree of freedom for optical information encoding. The authors convincingly demonstrate that the DOP can be controlled independently of the SOP and that it can be spatially structured with high fidelity. From a physical standpoint, this is an important and underexplored resource. The extensive scaling analysis and the demonstration of high-resolution DOP and SOP patterns strongly support the claim that this is not merely a proof-of-principle effect, but a scalable and broadly accessible capability.

Response: We are sincerely grateful to the Referee for these generous remarks and the careful consideration given to our manuscript. The report reflects a deep understanding of both the conceptual and technical aspects of the work. We particularly appreciate the recognition of the extensive efforts undertaken in the revision, many of which were motivated by the Referee's thoughtful and constructive comments.

Comment 4.1: The treatment of coherence has markedly improved. The manuscript now correctly frames the optical processing as partially coherent, and the supplementary polarization-matrix analysis provides a solid theoretical foundation linking the programmed phase statistics to the resulting DOP. The clarification that the nonlinearity is not in the propagation but in the field representation and detection is an important point that is now handled carefully.

Regarding the polarization-based encoding of RGB information, the revised manuscript is now more clear and more internally consistent than before. The inclusion of the Newton wheel in the original submission was confusing, as that color space contains only fully saturated colors, which can be mapped directly onto the surface of the Poincaré sphere (which motivated my initial impression). The authors now explicitly and correctly frame the DOP as a requirement to achieve a one-to-one, volumetric mapping between the full RGB color space and polarization space, as opposed to mappings confined to the surface of the Poincaré sphere. While one may debate definitions of what constitutes a "genuine" color encoding, the revised presentation is mathematically coherent and much less ambiguous than in the original manuscript. The added

experimental demonstrations of polarization-colored images at higher resolution further support this part of the work.
The optical encryption section has also been strengthened. The move to higher-resolution RGB plaintexts, the more careful discussion of reduced speckle contrast in partially polarized light, and the explicit analysis of robustness against cloning and misalignment attacks make this part of the manuscript more convincing and technically grounded. While the scheme remains a physical-layer encryption approach rather than a cryptographically rigorous one, it is now clearly presented as such, and its strengths and limitations are discussed more transparently.

Response: We are pleased that the Referee now finds the treatment of coherence and the polarization-color mapping clear, mathematically coherent, and strongly supported by the experimental polarization-colored images. We thank the Referee again for having identified the original ambiguity, which helped us improve this aspect of the work.

Comment 4.2: I am more ambivalent about the optical neural network results. Compared to the original submission, the relative improvement in the classification accuracy has decreased: the original manuscript reported an improvement by roughly a factor of 1.30, whereas the revised manuscript shows a more modest improvement on the order of 1.16. At the same time, the experimental and conceptual complexity of the system has increased significantly. In their response, the authors imply that the reduced absolute classification accuracy is due to the more challenging and realistic task (the full CIFAR-10 dataset at native resolution). However, they do not mention the reduction in the relative performance improvement with respect to the original submission. It would be helpful if the authors could comment on this. As it stands, one may reasonably question whether the added complexity associated with DOP and SOP encoding is fully justified on the basis on the moderate classification accuracy gains.

Response: As correctly noted by the Referee, the relative improvement factor is smaller than that reported in the original submission. This is a natural consequence of the significantly more challenging classification task implemented in the revised manuscript. The original gain was obtained on selected CIFAR subclasses whose categories were particularly distinguishable through color information. This suggests that the advantage provided by polarization encoding is naturally more pronounced in tasks where additional data dimensions carry highly discriminative information. For this reason, we did not emphasize the improvement on the original dataset, which was task-specific. In contrast, the current implementation relies on a standard and widely used benchmark, making the achieved performance directly comparable with the literature. Within this context, the observed improvement remains substantial (for example, improvements obtained through nonlinear speckle processing on the same task amount to nearly 3%). Nevertheless, the Referee’s question is entirely reasonable. We view the classification gain as a

first and readily quantifiable advantage of polarization encoding, which is also broadly applicable across different PNN architectures. At the same time, the main significance of the approach lies more fundamentally in the introduction of a different optical computing mechanism, whose implications extend beyond the observed accuracy improvement, as discussed below.

Comment 4.3: That said, there is one aspect of the ONN results that I find genuinely interesting and somewhat undersold in the revised manuscript. The observation that the classification accuracy increases systematically with the number of cascaded diffusers is a conceptual advance in my opinion. The supplementary VTM analysis makes a compelling case that this effect arises from the vectorial nature of light and from polarization mixing accumulating across layers, combined with the nonlinear nature of the Stokes-parameter representation and intensity detection. In this sense, the field itself carries the nonlinearity, effectively relaxing the need for explicit nonlinear activation functions between optical layers. Although the absolute improvement is quite small, the fact that such a depth-dependent enhancement exists at all in a purely passive, linear optical system is, in my view, one of the most conceptually significant results of the revised manuscript. Highlighting this aspect more clearly, and framing it as a statement about where the nonlinearity can reside in a physical computing system would further strengthen the broader impact of the work.

Response: We appreciate the Referee's perspective on this aspect and agree on the conceptual significance of the result. In the revised paper, we chose not to emphasize it in order to avoid distracting from the main contribution of the work, namely the spatial control of the DOP. However, we agree that the effect is noteworthy, particularly because its quantitative explanation in terms of polarization mixing accumulation points to a general physical mechanism rather than a specific observation. We believe this observation opens interesting perspectives for realizing nonlinear optical processing through polarization effects.

Following the Referee's suggestion, we have now highlighted this aspect more clearly in the introduction, emphasizing that nonlinear behavior can arise in a linear optical system through polarization-dependent interactions. Given the considerable effort currently devoted to implementing nonlinear operations in optical neural networks, we agree that this perspective may be of broad interest.

Comment 4.4: Finally, the manuscript as a whole is now much better balanced and more careful in its claims. The introduction and discussion are more clear, the figures are more informative, and the connection between experiment, theory, and simulation is substantially tighter.

While some of the application-level performance gains remain modest, the manuscript convincingly establishes spatial DOP modulation as a new and potentially powerful degree of freedom for optical information processing. Overall, this is a strong and thoughtful revision, and

I again commend the authors for the depth and seriousness with which they have addressed the referees' concerns.
From my perspective, the manuscript's suitability for Nature rests primarily on the conceptual advance of programmable spatial DOP control rather than on the magnitude of the demonstrated ONN performance gains.

Response: We are pleased that the substantial efforts devoted to the revision have resulted in a more mature manuscript and in a clearer, more balanced, and more rigorous presentation of the work. We thank the Referee once again for the important role that their observations have played in shaping the final version of the manuscript.
